# Position paper of the German Society of Oto-Rhino-Laryngology, Head and Neck Surgery and the German Society of Phoniatrics and Pediatric Audiology – Current state of clinical and endoscopic diagnostics, evaluation, and therapy of swallowing disorders in children

**DOI:** 10.3205/cto000117

**Published:** 2015-12-22

**Authors:** Christoph Arens, Ingo F. Herrmann, Saskia Rohrbach, Cornelia Schwemmle, Tadeus Nawka

**Affiliations:** 1 Department of Otorhinolaryngology, Head and Neck Surgery, University Hospitals Magdeburg, Otto-von-Guericke-University Magdeburg, Germany; 2 Reflux Center of Düsseldorf, Germany; 3 Department of Audiology and Phoniatrics, Charité – University Medicine of Berlin, Germany

**Keywords:** swallowing, disorders/etiology/diagnostics, aspiration, oropharynx/pathophysiology, laryngoscopy

## Abstract

Swallowing disorders are frequent. The main concern is mortality due to aspiration-induced pneumonia and malnutrition. In addition, quality of life is severely affected. The demographic trend indicates an increase of dysphagia in the future. Neurodegenerative diseases, tumors of the digestive tract, and sequelae of tumor treatment in the head and neck region are the main pathologic entities.

Predominantly ENT physicians and phoniatricians are asked for diagnostics and therapy, and will coordinate the interdisciplinary treatment according to the endoscopic findings.

A differentiated approach in history, diagnostics, and symptom-oriented treatment is necessary for these mostly complex disorders. Integration of non-medical staff such as speech therapists, physiotherapists, and occupational therapists in planning and executing an effective therapy expands and completes the patient-oriented care. Conservative treatment by these therapists is an important pillar in the treatment. Parts of the specific diagnostics can be taken over in close cooperation.

In particular, an interdisciplinary cooperation with the staff of intensive care medicine is essential.

The diagnostic procedures of specific endoscopy as described in this position paper are part of the primary and fundamental tasks of ENT specialists and phoniatrists.

Endoscopy is a medical service that is basically not delegable. Consequently, substitution of the physician is excluded.

## 1 Introduction

The term dysphagia encompasses all painless limitations of nutrition and of the regular transportation of food. It is more of a description of symptoms than a diagnosis. Swallowing as a central function includes transportation of saliva, secretion, liquid, and food from the mouth via the pharynx and the esophagus into the stomach. Beside neurological and muscular diseases, swallowing disorders may be triggered or enhanced by tumor- or surgery-related changes in the head and neck area, the esophagus, and the stomach [1].

In this context, the location, type of treatment, healing, and prognosis of the basic disease play a major role for the severity of dysphagia and its successful treatment.

This position paper will describe anamnesis, clinical examination, endoscopy, diagnostic evaluation of the findings, and decision-making regarding therapeutic possibilities and their consequences, i.e. all procedures that physicians should undertake to treat patients suffering from dysphagia. Endoscopy of the upper aerodigestive tract plays a key role in the diagnostics of dysphagia. Phoniatricians or ENT specialists/head and neck surgeons not only assess the functional swallowing process during endoscopy but also possible structural variations of the upper aerodigestive tract. Flexible endoscopic swallowing examination is the basis of medical activity in this position paper issued by the German Society of Oto-Rhino-Laryngology, Head & Neck Surgery and the German Society of Phoniatrics and Pediatric Audiology. It does not correspond to fiberoptic endoscopic evaluation of swallowing (FEES), which is a term for a standardized examination procedure with systematic evaluation of the findings [2].

Flexible endoscopy requires a safe handling of the endoscope as well as profound knowledge of the anatomy, physiology, and pathophysiology of the upper aerodigestive tract. Possible complications during preparation and performance of flexible endoscopy make it essential to keep emergency equipment at hand. All participating persons need to be familiar with it. Because the symptoms of dysphagia may be multi-dimensional, each one has to be considered carefully.

From a medico-legal point of view, the following aspects are important. Before flexible endoscopy can be applied, the patient must be informed and must consent to the examination. Only physicians are allowed to perform flexible endoscopy. Flexible endoscopy of the upper aerodigestive tract belongs to the discipline of oto-rhino-laryngology or phoniatrics and pediatric audiology [3].

## 2 Anatomical bases and physiology of swallowing

### 2.1 Pre- and postnatal swallowing

Between the first swallowing observed in the 11^th^ to 12^th^ week of pregnancy and a nearly completed swallowing competence at the age of about four years, a very complex structural and functional development takes place [4].

In the embryonal stage, i.e. 1^st^ to 8^th^ week of pregnancy, the following steps can be observed. In week 3, a primitive mouth develops; in week 4 pharyngeal arches and pouches become obvious, from which the face, nose, mouth, larynx, and pharynx later develop.

During the fetal period, i.e. from 9^th^ week of pregnancy to birth, the following steps are observed. In week 10, the glottis is open; in week 13–16, pharyngeal swallowing starts with separate movements of the lips, mouth, pharynx, and esophagus. In week 17–20, suction starts. In week 26–29, primitive reflexes occur such as phasic biting or retching. The fetus stimulates its mouth, reacts with mimics on bitter substances in the amniotic fluid, and swallows up to 400 ml amniotic fluid per day. As of week 32, the fetus is able to suck and to swallow, so that premature babies at this age can be breast- or bottle fed.

After birth, neonatal reflexes are observed which are related to food intake and swallowing. These are, among others, coughing, retching, phasic biting, the transversal tongue reflex/protrusion, the rooting reflex, the palmar grasp reflex (holding food), the Babkin reflex (coordinated movement of the hand to the mouth), and the palmomental reflex (coordinated movement of the hand to the mouth). These primitive reflexes are suppressed during the first year of life as the brain successively matures.

In general, 50 pairs of muscles and fife brain nerves are involved in swallowing. During this action, breathing stops (so-called deglutition apnea) and the larynx closes. In this way, the deep airways are protected.

### 2.2 Phases of swallowing

For liquids and solid or semisolid food, the phases of swallowing are different. Liquids are swallowed from the oral cavity into the esophagus without interruption. The phases of swallowing of solid or semisolid food are classified according to the location of the nutrition bolus. They merge into one another. The different stages are:

1. Preoral or anticipatory phase
2. Oral preparatory phase
3. Oral phase
4. Pharyngeal phase
5. Esophageal phase

#### 2.2.1 Preoral and anticipatory phase

Food and beverages are already checked prior to opening the mouth (odor, temperature, appearance). Touching the food with the lips completes this check.

#### 2.2.2 Oral preparatory phase

In the oral preparatory phase the food is again checked for odor, taste, temperature, and volume. In case of disapproval, the bolus can be spit out, i.e. this phase can be influenced voluntarily, but nonetheless it is automatized. A positive feedback has a stimulating effect on triggering the swallowing reflex. In the oral preparatory phase, the bolus is formed and positioned. First the food intake occurs, then the food is masticated, if needed, and moistened by saliva.

The mimic muscles cause a sufficient lip closure, also by the tonus of the orbicularis oris muscle. The buccinator muscle regulates the buccal tonus. Food parts that happen to get into the lateral vestibule are pushed back between the teeth. The VII. brain nerve, the facial nerve, innervates the mimic muscles. The temporomandibular joint is formed by the mobile head of the mandible (caput mandibulae) and its mobile cartilage disc (discus articularis) and the bony mandibular fossa (fossa mandibularis). For the chewing movement, the lateral movement of the mandible is particularly important. The 32 teeth of adults are firmly anchored with their roots in the maxilla and the mandible; their position is in occlusion. Mastication is a cyclic movement with perfectly matched movements of the mandible, cheeks, tongue, and hyoid. Sensomotor control avoids bite wounds. The chewing muscles consist of 4 muscles innervated by the branches of the mandibular nerve. The temporalis muscle, the masseter muscle, and the medial pterygoid muscle, which also causes a movement forward, close the mandible. The lateral pterygoid muscle opens the mouth with bilateral contraction.

The bony frame for the floor of the mouth is built by the mandible. At the inner inferior side, the geniohyoid muscle runs alongside and the mylohyoid muscle runs across.

The tongue shapes the bolus and pushes it between the teeth (tip and edges of the tongue press against the alveoli). With the saliva, the bolus is mixed to a more homogenous mass. Elevation of the postero-dorsal part of the tongue and lowering of the velum prevents an early passage of the food into the pharynx.

At the end of the preparatory phase, the gap between the postero-dorsal part of the tongue and the velum opens and introduces the transition from the oral to the pharyngeal phase. 

While chewing, small pieces of the food slide via the V-shaped trough (median lingual sulcus) into the valleculae. 

In this phase, the bolus in the oropharynx does not cause any risk of aspiration because of the protective function of the epiglottis [5]. In cases of semifluid consistency, the bolus overflows earlier. If part of the bolus slides over the important trigger zone of the plicae pharyngoepiglotticae, the swallowing reflex is triggered.

Small quantities of liquid can run between the base of tongue and velum into the valleculae without triggering the swallowing reflex. During expiration, scent molecules of the bolus reach the olfactory region via the retronasal pathways. In this way, sensory information is passed via the receptors to the swallowing center and swallowing is avoided or promoted. 

The duration of the individual oral preparatory phase may vary significantly.

#### 2.2.3 Oral phase

During the oral phase, the bolus is propelled from the mouth into the oropharynx. It can be controlled voluntarily and starts with the movement of the tip of the tongue and ends when the oral cavity is empty. The tongue works like a piston against the hard palate. It is supported by the lateral excursions of the jaw resulting in rotatory movements. Because the tongue moves up and down, it helps to press the food between the teeth. If the food is crushed, the tip of the tongue and the anterior lingual edges are in close contact with the alveoli.

The lingual muscles insert in the floor of the mouth and are innervated by the hypoglossal nerve. A distinction is made between the extrinsic muscles (genioglossus muscle, hypoglossus muscle, styloglossus muscle) and the intrinsic muscles (longitudinal muscle, vertical muscle, transverse muscle). During contraction, the extrinsic lingual muscles draw the whole lingual body with the bolus in an anterior-posterior direction. The intrinsic lingual muscles transport the bolus in a wavelike movement along the palate in a posterior direction. Closing the mouth supports this action. Between the soft palate and the tongue, the “door” can be opened in a differentiated way in order to let some drops or a whole bolus pass.

The oral phase can be impaired or even missing when saliva and/or remaining food is to be swallowed.

#### 2.2.4 Pharyngeal phase

The pharynx is a muscular tube which is about 12–15 cm long and extends from the skull base to the esophageal entrance. In this space, the trachea and the esophagus meet. Pharynx and esophagus are elastically fixed between the skull base and the diaphragm. At the same time, the trachea is elastically connected with the hyoid and the larynx between the skull base and the mandible on one side and the superior thoracic aperture on the other via muscles and ligaments. These facts make it obvious that the function of the whole system may be influenced, for example, by hiatus hernia.

The pharynx is subdivided into three levels. The nasopharynx extends from the pharyngeal roof to the velum and is connected via the choanae with the nasal cavity and via the auditory tube with the middle ear. The oropharynx extends from the velum in caudal direction to the plica pharyngoepiglottica and opens in anterior direction to the oral cavity. The hypopharynx extends anteriorly from the epiglottis, and laterally via the aryepiglottic fold to the interarytenoid incisure. The piriform recess is located lateral of the aryepiglottic fold and reaches to the superior end of the esophagus. The hypopharynx is open to the larynx and the esophagus.

The pharyngeal tube is formed by the 3 pharyngeal constrictor muscles and the pharyngeal levator muscles. The pharyngeal constrictor muscles consist of the superior pharyngeal constrictor muscle (innervated by the glossopharyngeal nerve, N. IX), which approaches the velum as an annular bulge closing the nasopharynx. A circular muscle before the nasal meatus, called the rhino-sphincter by M. Strahl, is present in about 9% of outpatient ENT patients [6]. It closes before velum closure and opens for a short time after relaxation. It is assumed to have a protective function for the nasopharynx and thus for the nose, paranasal sinuses, auditory tube, and middle ear.

Further muscles such as the middle pharyngeal constrictor muscle (pharyngeal plexus, glossopharyngeal and vagus nerves, N. IX and N. X) and the inferior pharyngeal constrictor muscle (N. X) support propulsion of the food bolus.

In the pharyngeal phase, the bolus is pressed into the hypopharynx due to peristalsis of the tongue. During relaxed endoscopy, it becomes obvious how the bolus slopes into the vallecula through the slightly concave trough of the tongue below the mobile uvula. The lingual bone is elevated and moves in a ventral direction. Supported by the thyrohyoid muscle, the larynx is lifted anteriorly, resulting in a suction effect through the opening of the hypopharynx (enlarged space). At the same time, closing mechanisms are induced. The velum moves to the posterior wall of the pharynx, the epiglottis closes the larynx, the supraglottic space narrows, the vocal folds and the false vocal folds close, and the arytenoids incline into the lumen and lay over the vocal folds. The epiglottis is lifted through contraction of the thyrohyoid muscle and at the same time it lowers the upper edge against the posterior wall of the pharynx in order to receive the bolus. Hereby, the position of the hyoid bone remains relatively stable.

The swallowing reflex is triggered according to the quality of the stimulus at the anterior palatal arches, the plicae pharyngoepiglotticae, or the postcricoid mucosa [6]. It is inter- and intra-individually variable, depending on the bolus and the person’s age. It cannot be influenced voluntarily. Triggering occurs exclusively through tactile stimuli (thermal stimuli do not play a physiological role). 

Aqueous substances are swallowed continuously in a split second. Viscous and solid food are collected in the vallecula and swallowed in cascades. This is why it is possible to speak with a vallecula filled with food, whereas this is excluded in the case of liquids.

In infants, the hard palate without teeth is flat and the positions of the cricoid cartilage and the larynx are more cranial than in adults. The epiglottis moves behind the soft palate, the larynx opens directly into the nasopharynx. The airway is separated from the esophagus by the elevated larynx, which prevents aspiration. When sucking, infants can thus breathe without aspirating, although swallowing is also associated with deglutition apnea, as in adults.

Due to the relevant reverse movement of the base of tongue at the posterior wall of the pharynx, which also contracts, the tongue works as a stamp when transporting the bolus. Simultaneously, the hyoid bone and the larynx move in an anterior and superior direction. The geniohyoid and the mylohyoid muscles move the hyoid bone, which enlarges the pharynx and shortens it by about one third.

To protect the deep airways, the larynx closes in four levels: closure of the vocal folds; close contact of the false vocal folds; approaching of the arytenoid cartilage to the petiolus; and dorsal movement of the epiglottis. During swallowing, reflex apnea occurs.

At the end of the pharyngeal phase, the upper esophageal sphincter opens, as first described by Killian. According to manometric, electro-myographic, and radiological observations, the closure zone of the upper esophageal sphincter is not limited to the cricopharyngeal muscle. Caudal parts of the thyropharyngeal part of the inferior pharyngeal constrictor muscle and the cranial parts of the cervical sphincter of the esophagus are also functionally included [7]. A submucous layer of the postcricoid region supports closure of the upper esophageal sphincter as a cavernous pad.

Opening of the upper esophageal sphincter occurs in several steps:

- Relaxation
- Opening
- Enlargement of the opening
- Collapse
- Closure

The upper esophageal sphincter relaxes about 0.1 second before elevation of the larynx. Due to contraction of the thyrohyoid muscle with slight tilting and elevation of the epiglottis followed by forward movement of the larynx in an anterior-superior direction, the muscle between larynx and posterior pharyngeal wall opens passively before the bolus reaches this area. The contractions of the constrictor muscles press the free edge of the epiglottis downwards with the lingual stamp. Because of this mechanism, the bolus slopes into the upper esophagus like a roof avalanche. The pressure of the bolus influences the width of the opening of the upper esophageal sphincter. As soon as the bolus has reached the esophagus and the larynx together with the hyoid bone are once more in a relaxed position, the upper esophageal sphincter closes [8]. While the velum is already relaxed, the epiglottis is kept closed by the persisting caudal contraction over the laryngeal entrance. As soon as the epiglottis is in its relaxed position, the larynx re-opens for breathing and the pharyngeal phase is passed.

#### 2.2.5 Esophageal phase

The esophagus starts at about the level of the 6^th^ cervical vertebra and forms an elastic tube measuring about 25 cm. The whole distance from the incisors to the stomach entrance amounts to about 40 cm. The esophagus is innervated by the vagus nerve via the esophageal plexus and the sympathetic trunk. Peristalsis is promoted by the vagus nerve and inhibited by the sympathetic nervous system.

The esophagus merges into the stomach in an acute-angled way (cardiac incisura, angle of His).

Because of the anatomy, the esophagus is constricted in three places: first, where the pharynx joins the esophagus behind the cricoid cartilage, which is the narrowest passage of the esophagus (about 15 mm in diameter); second, where the esophagus is crossed in the front by the aortic arch; and third, where it passes through the diaphragm. Changes of surrounding structures in the area of these narrow segments can lead to swallowing disorders.

The superior segment is closed by the upper esophageal sphincter. In the upper quarter, the esophagus consists of striated muscles; in the second quarter additionally of smooth muscles; and in the inferior parts only of smooth muscles. Exteriorly, the muscle fibers run helically (exterior longitudinal muscles) and, on the inside, they are diagonal or circular (interior sphincters), forming a functional unit [9].

Regarding peristalsis, a primary and a secondary wave are distinguished. Transportation of the bolus with the reflex-induced primary wave takes between 4 and 40 seconds until the stomach is reached. With opening of the upper esophageal sphincter and insufficient lower esophageal sphincter, liquids can inject into the stomach in only one second due to the stamp force of the tongue, floor of the mouth, and Killian muscle. If a hiatus hernia is present, the esophagus loses its tension and collapses. In an upright position, transportation of the food bolus is supported by gravity.

Mechanical stimulation (by remaining food) at the wall triggers the secondary wave. It works as a type of cleaning. The esophagus has a high longitudinal tension, even allowing swallowing in a headstand.

### 2.3 Central control of swallowing

The complex control of the swallowing procedure takes place in different cerebral structures. There are connections in both directions between the cerebral cortex, the cortico-bulbar ways, the brain stem, and the peripheral swallowing muscles.

Structures are found in the superior medulla oblongata called “Central Pattern Generators for Swallowing” (CGPs) [10]. A distinction is made between a dorso-medial (“Dorsal Swallowing Group”, DSG) and a ventro-lateral (“Ventral Swallowing Group”, VSG) part. In the dorsal part, the spatial and temporal interplay of swallowing muscles is coordinated. The nucleus of the solitary tract is part of the posterior swallowing group and processes sensitive stimuli from the oropharyngeal area (temperature, contact, etc.). From the ventral group, this information is transmitted to the nuclei of the cerebral nerves relevant for swallowing (V, VII, IX, X, and XII). 

The relevant areas of the cortex are, among others, the fronto-parietal operculum and the anterior insular cortex, which is active in particular in the voluntary swallowing act [11].

Independent of handedness, there is an interindividual difference in swallowing dominance, which means that one hemisphere of the cerebrum has a bigger area of the cortex responsible for swallowing than the other [12].

According to numerous investigations (fMRI, PET, studies of specific lesions etc.), it is assumed that the left hemisphere is probably more responsible for the oral preparatory phase and the oral phase, while the right hemisphere dominates in the pharyngeal phase [13], [14]. It is typical for unilateral lesions of the dominant swallowing cortex that triggering of the swallowing reflex is delayed [15].

The course of the esophageal phase is mostly independent, but it is also subject to central influences, especially from the medulla oblongata [16].

A summary of the different swallowing phases is given in Table 1 (Tab. 1).

## 3 Clinical aspects of dysphagia

### 3.1 Pathophysiology of swallowing

#### 3.1.1 Classification of dysphagia according to the location

According to the location of the lesion, different symptoms occur. Table 2 (Tab. 2) shows a summary of this observation. Dysphagia may become apparent in every stage of life. Pathophysiological aspects in pediatric patients are discussed in the chapter on diagnostics of pediatric dysphagia.

Disorders of the preoral phase may lead to a missing sensation of hunger or thirst, insufficient visual assessment of food, or manual insufficiencies regarding the bite-sized preparation of the food [17]. In this paper, these problems will not be described in detail.

#### 3.1.2 Structural changes

**Figure 1:**
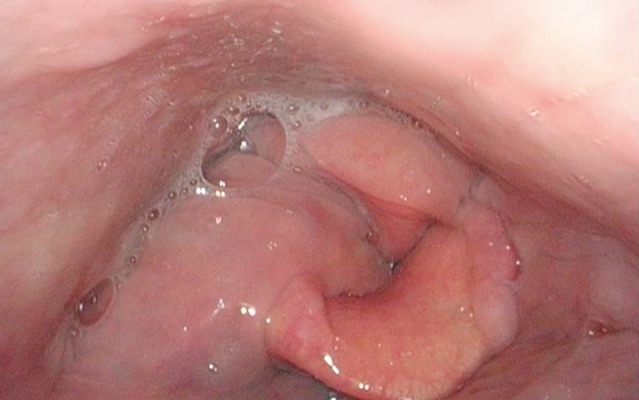
Transnasal endoscopy performed 8 days after cricohyoid epiglottopexy, postoperative swelling without detectable lumen, aspiration, and salivary retention

The quality and appearance of the mucosa, symmetry of the structures, defects, scars, edema, and protrusions of the posterior pharyngeal wall are assessed in a stage of calm respiration. In patients who have undergone head and neck tumor therapy, changes of the mucosal surface may impair the ability to swallow. Mucosal changes are, for example, reddening, swelling, ulceration, later also atrophy, scarring, and dryness (Figure 1 (Fig. 1)). Clumsiness of the epiglottis impairs the regular dorsal inclination during swallowing and thus represents a risk of aspiration [18].

Reflux is particularly dangerous for patients with dysphagia because gastric juices may be aspirated. Signs of reflux are edemas of the laryngeal structures, pseudo-sulcus of the vocal folds due to infraglottic edema of the anterior commissure to the posterior wall of the larynx, thickened mucosa of the interarytenoid region, increased vascular drawing, ulceration and granulations of the arytenoids and vocal folds as well as viscous mucus in the hypopharynx and larynx [19], [20].

#### 3.1.3 Pareses

**Figure 2:**
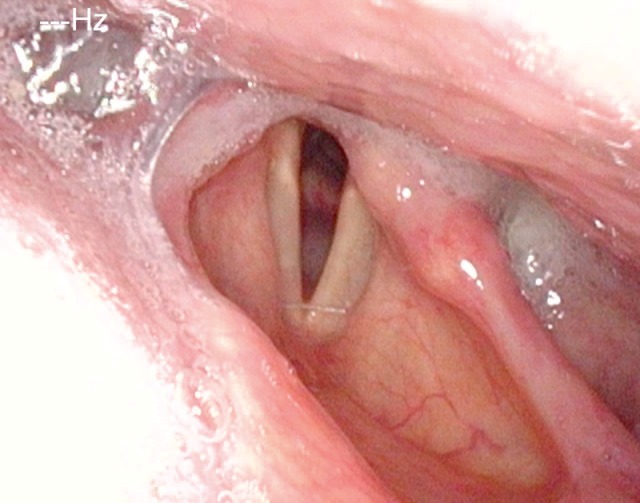
72-year-old female patient with lingual goiter and left-sided paresis of the vocal folds. Visible salivary retention in the piriform recess, penetration, silent aspiration.

**Figure 3:**
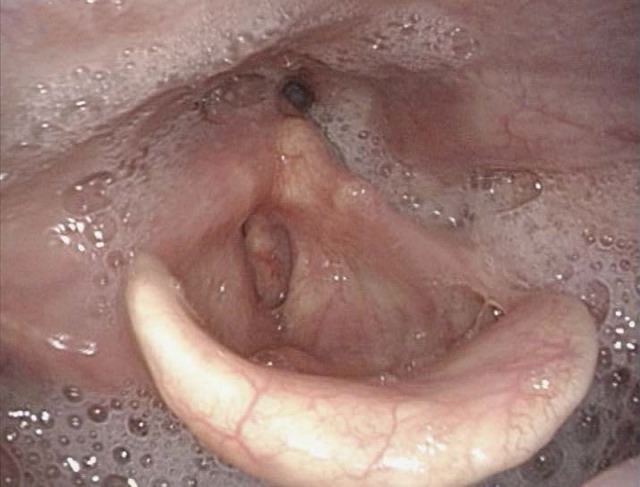
70-year-old female patient with bilateral paresis of the vocal folds after total thyroidectomy, neck dissection, radiation. Chronic radio-dermatitis. The larynx is clearly displaced in a ventral direction due to scars. Impaired supraglottic sensitivity. The upper esophageal sphincter is open, pooling of foamy saliva. Penetration.

Central and peripheral pareses may lead to insufficient glottis closure during swallowing. In a study of 2,650 patients with dysphagia, 4.5% had pareses of the vocal folds. The aspiration rate amounted to 37% for right-sided paresis, 42% for left-sided paresis, and 50% for bilateral paresis. The risk of aspiration, especially of liquid, was 2.5-fold higher than for patients without paresis [21]. Because of the motor and sensitive functions going beyond the mobility of the vocal folds, the risk of aspiration increases significantly in cases of paresis of the glossopharyngeal nerve, the vagus nerve, and the hypoglottic nerve (Figure 2 (Fig. 2), Figure 3 (Fig. 3)).

#### 3.1.4 Hyperkinesia

Involuntary rhythmic movements of the velum of the posterior pharyngeal wall and the larynx with frequencies of 1–3/s with quicker adduction and slow abduction are very frequent in patients with a lesion of the cerebellum and/or brain stem, at rest (even when sleeping) and in the case of voluntary and reflex movements. Sometimes they are already visible from the exterior as nystagmus-like movements of the larynx and/or the floor of the mouth or the cervical muscles. Since glottic closure is possible, although not synchronized with the swallowing phases, functionally insufficient glottic closure may lead to aspiration [22].

#### 3.1.5 Sensitivity disorders

Symptoms indicating sensitivity disorders of the larynx are the transition of saliva or chime into the glottis with and without coughing reflex, irritation in the glottis and subglottis like reddening and vascular injection as well as gargling and rough voice quality (Table 3 (Tab. 3)).

Saliva and accumulations of secretion at the hypopharyngeal walls, in the valleculae, the piriform recesses, and in the post-cricoid region are always a sign of a swallowing disorder. They may also be observed in the entrance of the larynx, in the glottis, and in the subglottis up to the trachea. If they do not trigger the coughing reflex, this phenomenon is called silent aspiration, which may be life-threatening in the long-term, especially if the patient is not even able to cough voluntarily. The identification of accumulations of saliva and secretion is important in order to assess the risk of a patient with dysphagia [23], [24]. In this context, endoscopic examinations represent a clear advantage because these substances cannot be visualized by radiology [25].

In case of missing sensitivity, an early uncontrolled passage of substances into the pharynx occurs before triggering of the pharyngeal reflex phase, substances accumulate in the hypopharynx, and pre-deglutitive penetration/aspiration may result. Table 3 (Tab. 3) summarizes the terms regarding the description of swallowing disorders.

A delayed triggering of the swallowing reflex is characterized by delayed movement in an anterior and superior direction of the arytenoids [26], [27], and by delayed elevation and lowering of the base of tongue. When the bolus passes into the valleculae or the piriform recesses, the elevation of the larynx is delayed and the epiglottis does not incline in a dorsal direction. The dorsal flexion of the epiglottis during the pharyngeal phase is insufficient and reduced, which is, in the context of fiberoptic examinations of swallowing, indicated by the shorter duration of “white out” (superimposition of the camera caused by contact with the mucosa during laryngeal closure) [27], [28].

**Figure 4:**
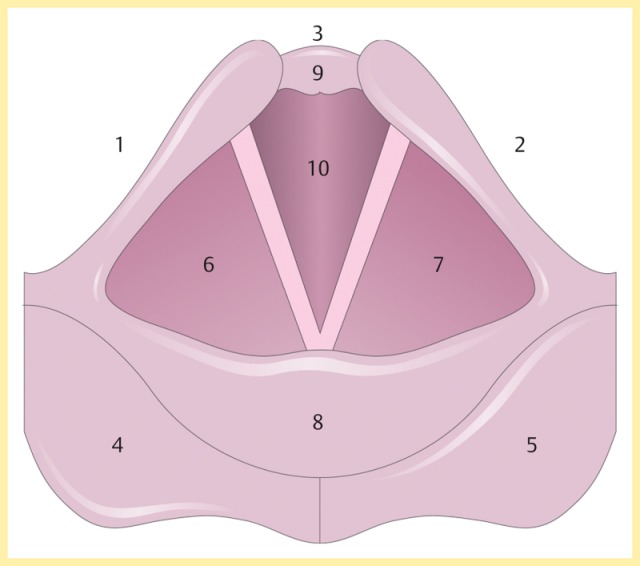
Laryngeal scheme with location of residues (1–5), penetration (6–9), aspiration (10) [29]

After the end of the pharyngeal phase, the classic signs for dysphagia can be observed transnasally or transorally [29] (Figure 4 (Fig. 4)).

1. Residues of substances at the pharyngeal walls, in the valleculae and the piriform recesses, in the post-cricoid region with/without attempted clearing of the pharynx
2. Penetration of substances into the laryngeal entrance, at the laryngeal surface of the epiglottis, via the aryepiglottic folds, via the interarytenoid region with/without triggering the coughing reflex
3. Aspiration of substances into the glottis, into the subglottic region with/without triggering the coughing reflex

#### 3.1.6 Functional cricopharyngeal disorders

Functional cricopharyngeal disorders caused by sphincter hypertrophy with functional stenosis are, secondarily, mostly a sequel of an impaired excursion of the hyoid bone and the larynx. Primarily, they are the result of disturbed coordination between pharyngeal propulsion and missing esophageal relaxation, e.g. after brain stem lesions, in the context of idiopathic Parkinson’s disease or myositis. Functional cricopharyngeal disorders lead to retention of food in the cricopharyngeal passage with the risk of post-deglutitive, potentially life-threatening aspiration especially in pediatric and old patients. After laryngectomy, functional cricopharyngeal disorders are even increased by adjuvant radiation and not only impede swallowing but also phonation via the voice prosthesis due to a reduced air passage with consecutively reduced swinging of the pharyngo-esophageal segment [30].

#### 3.1.7 Presbydysphagia

Age-related physiological changes of the swallowing procedure are defined as presbyphagia [17], [31], [32]. The anatomical and physiological processes of a regular swallowing procedure, i.e. accordingly coordinated swallowing and senso-motor integration of different anatomical units, muscles, nerves, and cerebral centers, change during the aging process, especially in the “Fourth Age”, dementia. Reduced gustatory and olfactory senses, cerebral degenerative processes, and changes of the quality and quantity of neuro-muscular coordination processes have a mostly negative influence on the swallowing procedure, which may affect all three phases of swallowing. The effectiveness is reduced and adaptation of food intake becomes necessary [17]. The esophageal phase is prolonged with increasing age. These presbyphagic changes are usually compensated [33]. Only when such compensation strategies are no longer sufficient does dysphagia result, which is then called presbydysphagia.

When the upper esophagus is pathologically enlarged, the food is pushed back into the pharynx when the pressure is reduced at the end of the pharyngeal phase. In this way, the time of bolus passage of 0.7 s doubles to 1.4 seconds without elevation of the epiglottis [34]. The patient notices the problem of swallowing when the duration of bolus passage is more than four times longer [35]. Furthermore, patients of increased ages frequently have concomitant diseases that add to the swallowing disorders and may also cause dysphagia. A distinction should be made between these disorders and presbydysphagia, although it might be difficult to find an exact delineation [36]. Table 4 (Tab. 4) shows which age-related changes of the single swallowing phases may lead to presbydysphagia.

### 3.2 Diseases of the head and neck with dysphagia

Swallowing disorders are always life-threatening. Since the consequences do not manifest acutely, chronic complaints in the context of dysphagia are mostly underestimated.

Diseases of the upper aerodigestive tract with cerebral, neurological, or traumatological origins as well as consequences of tumor treatment due to structural defects, scars, damage of brain nerves and irradiation [37] may cause swallowing disorders.

Cerebral damage or diseases are often associated with loss of consciousness and disturbed breathing and circulation in the acute stage. Maintenance of vital functions through long-term intubation and probe nutrition may lead to subsequent lesions in the area of the pharynx and larynx in addition to any damage caused by the original disease. Furthermore, patients with, for example, severe cranio-cerebral trauma often have other lesions such as peripheral nerve lesions, fractures of the mandible, and injuries of the soft parts besides their cerebral injuries. The consequences of global neuropsychological disorders, such as reduced attention and motivation, may also impair the swallowing process in the long-term.

#### 3.2.1 Oral and pharyngeal diseases

##### 3.2.1.1 Disorders of the preoral phase

The preoral phase, i.e. the olfactory and gustatory anticipation of food and the initiation of the regulatory swallowing (reflex) cycle with potential “blank” swallowing or spontaneous salivation, may already be disturbed due to innate or acquired olfactory disorders. 

##### 3.2.1.2 Disorders of the oral phase

**Figure 5:**
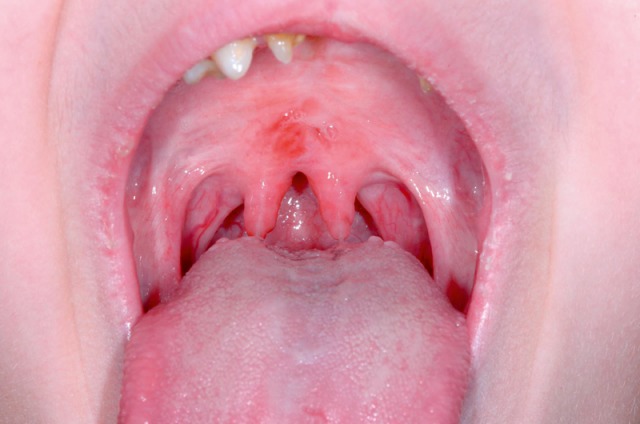
16-year-old patient suffering from Johanson-Blizzard syndrome. Incompletely treated cleft lip and palate and velopharyngeal insufficiency with passage of food and liquids into the nose.

Among the congenital diseases that might impair predominantly the oral phase, innate malformations should be mentioned such as isolated or combined cleft lip and palate (CLP), as well as congenital pareses of the facial muscles and morphological malformations, e.g. special genetic syndromes (Figure 5 (Fig. 5)).

**Figure 6:**
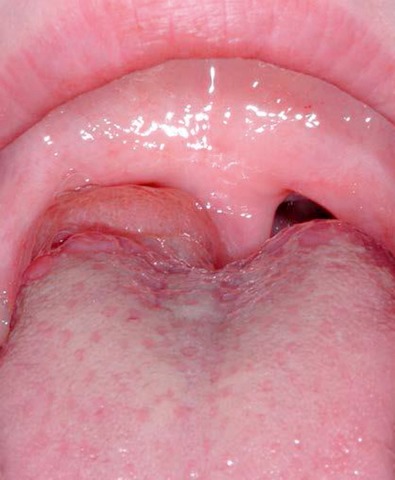
Lymphangioma of the right base of tongue. Anamnestically increasing, disturbed oropharyngeal phase.

**Figure 7:**
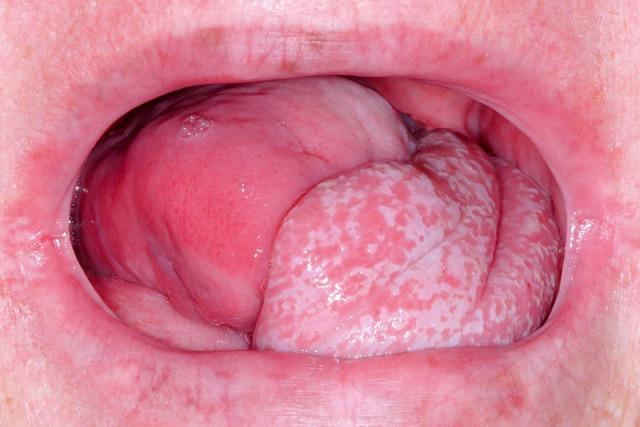
53-year-old female patient with left-sided reconstruction of the tongue after hemiglossectomy. Combined radial flap with neurovascular, infrahyoid, myofascial flap according to Remmert. Partially disturbed bolus transportation in the oral phase.

**Figure 8:**
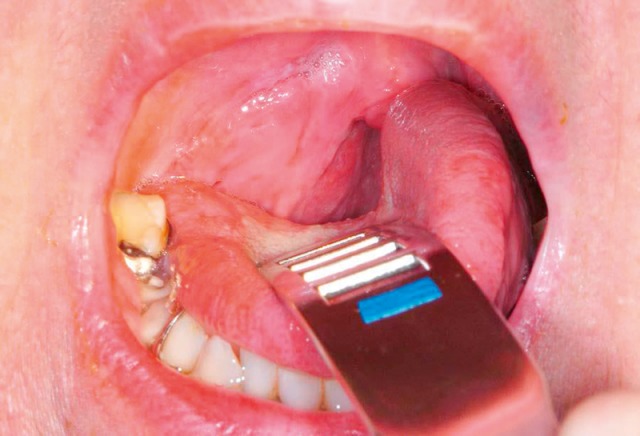
69-year-old female patient with right-sided parapharyngeal neurofibromas and increasing dysphagia. Uvula displacement to the left side. Impaired swallowing in the oropharyngeal phase.

Jaw opening for food intake and masticatory movement of the mandible may be impaired pre- and postoperatively in cases of tumor diseases but also due to radiation-associated tissue atrophy (so-called trismus). Especially tumors of the oral cavity, and here in particular of the tongue (Figure 6 (Fig. 6)), lead to severe problems in the context of bolus formation that may even increase with partial resection of the tongue and the floor of the mouth (Figure 7 (Fig. 7)). Parapharyngeal tumors may also be an origin of dysphagia (Figure 8 (Fig. 8)). Furthermore, leaking may cause disturbed bolus transportation and pre-deglutitive aspiration.

**Figure 9:**
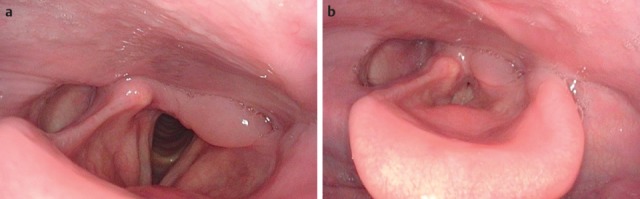
46-year-old patient with persisting dysphagia after gunshot to the left side of the neck with perforation of the pharynx. Emergency treatment 3 months ago. No opening of the piriform recess. Persisting swelling of the left arytenoid region and the aryepiglottic folds, retention of saliva. a – respiration, b – phonation.

Further acquired disorders are scarring after surgery, fractures, and different kinds of damage due to exogenous trauma such as impalement, burn injuries, or cuts (Figure 9 (Fig. 9)). In the case of partial paresis of the buccofacial or lingual muscles, myofunctional disorders, or postoperative scarring at the tongue, food intake, bolus formation (because of impaired lateral movement, trough formation, stamping pressure of the tongue) and bolus transportation may be disturbed. Even cranio-mandibular dysfunctions impede the oral phase, especially jaw opening and mastication of the mandible.

Moistening of the food with saliva requires undisturbed salivation. Xerostomia may occur due to medication or there may be, for example, a disorder based on rheumatic disease (Sjögren’s syndrome). The main cause of hyposalivation, however, is radiation-induced destruction of salivary gland tissue.

Compromised motility of the velum and velo-pharyngeal closure significantly impair quality of life. This is due to congenital (CLP) as well as traumatic or iatrogenic defects, or scarring after extensive resections of oropharyngeal tumors. Symptoms are hyperrhinophonia and rhinolalia, impaired triggering of the swallowing reflex, disturbed or incorrect bolus transportation, or early passage of the bolus into the pharynx without coordinated swallowing process.

#### 3.2.2 Disorders of the pharyngeal phase

##### 3.2.2.1 Tumorassociated dysphagia

**Figure 10:**
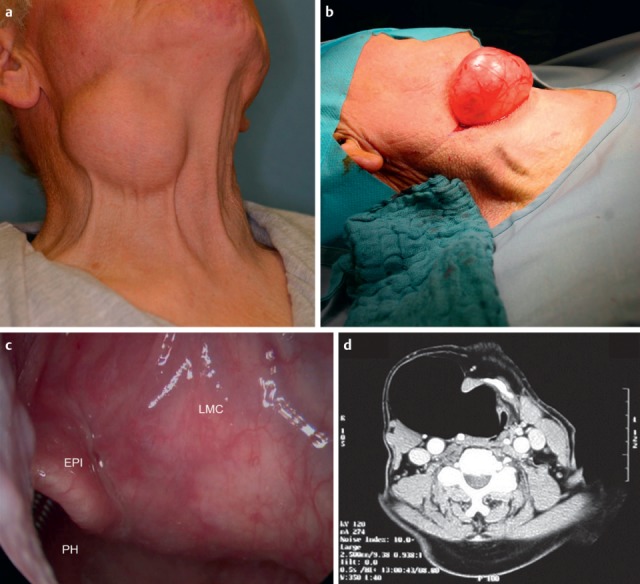
73-year-old patient with advanced right-sided internal and external laryngocele. Increasing dysphagia and muffled speech: a – clearly prominent mass at the right side of the neck; b – intraoperative situation; c – preoperative endoscopic image (LC – laryngocele, EPI – epiglottis, PH – posterior pharyngeal wall); d – horizontal CT scan of the neck with advanced right-sided laryngocele.

Once they reach a certain size, all pharyngeal tumors impair the swallowing process (Figure 2 (Fig. 2), Figure 10 (Fig. 10)). Generally, these tumors are squamous cell carcinomas. Dysphagia is caused by the tumor or its metastases. Regarding the severity of dysphagia, tumor size, infiltrating structures of hypopharynx and larynx as well as functional loss are relevant. Especially hypopharyngeal tumors remain undetected and, since there are no anatomical barriers, can expand in an unhindered way. Tumors affecting the larynx and hypopharynx may lead to combined dysphagia and dysphonia. Postoperatively, those tumors are characterized by substantial loss and changed anatomy. Dysphagia occurs, for example, when a bolus passes unhindered into the glottis. Damage to the superior laryngeal nerve due to partial resections or after thyroidectomy lead to a reduced protection reflex (coughing) and sometimes subsequently to silent aspiration. In particular after resection of parts of the epiglottis or the aryepiglottic folds, swallowing has to be re-learned. These mostly transitory swallowing disorders must be distinguished from chronic swallowing disorders caused by scarring and edematous swellings, for example after radiation.

**Figure 11:**
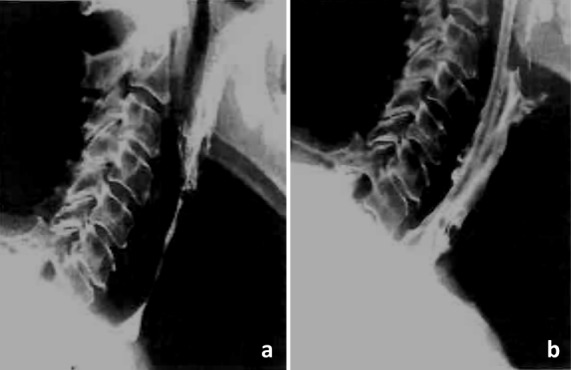
Laryngeal stenosis after laryngectomy and subsequent radiation: a – prior to surgery; b – skin-platysma-fascia flap [278].

After laryngectomy, transportation of the bolus may be impaired, e.g. because of scar strictures with stenosis of the neopharynx and the upper esophagus in the pharyngo-esophageal segment (Figure 11 (Fig. 11)). Aspiration may occur because of a tracheo-esophageal fistula with vocal valve.

##### 3.2.2.2 Radio-chemotherapy and dysphagia

Dysphagia is a relevant side effect of radiotherapy in the context of head and neck cancer and may even lead to limitation of the dosage [38]. The swallowing function may be disturbed by edema, neuropathy, and fibrosis. Acute mucositis and edema impair the swallowing function during radiation therapy. However, in most of the patients, these complaints are clearly regredient in the months following radiotherapy or radio-chemotherapy. In contrast, neuropathy and fibrosis of oral, laryngeal, and pharyngeal muscles may develop after the end of radiotherapy. According to Nguyen [39], dysphagia occurs more frequently in the context of radio-chemotherapy in comparison to radiation alone. Aspiration was generally observed in both groups. Frowen et al. [40], [41] report about the long-term results of dysphagia in patients with head and neck tumors who were examined regarding their swallowing function 6 months and 5 years after poly-chemo-radiotherapy. Factors of poorer postoperative swallowing function were former severe alcohol consumption, advanced tumor stage (T4), location of the tumor in the hypopharynx, bilateral radiation of the pharynx as well as rural origin of the patient. The most frequent predictor of dysphagia was the T classification, followed by history of alcohol abuse and type of radiation. After 5 years, 74% of the patients had recovered to their preoperative functional state. In contrast, 24% observed a significant deterioration of the swallowing function even after 5 years. Van den Berg et al. [42] described swallowing disorders after polychemo-radiotherapy in 75% of head and neck cancer patients. In 57%, pathological findings could be observed during video-fluoroscopy. According to Gourin et al. [43], pretherapeutic dysphagia, radio-chemotherapy, and salvage surgery are significant predictors for long-term dysphagia in elderly patients suffering from laryngeal carcinoma. On the other hand, long-term dysphagia, the presence of PEG and/or tracheostomy, weight loss, airway obstruction, and pneumonia may be associated with a poorer survival rate. Especially in the context of pneumonia, the highest mortality rate was observed in relation to a 5-year survival rate.

##### 3.2.2.3 Hypopharyngeal diverticulum

**Figure 12:**
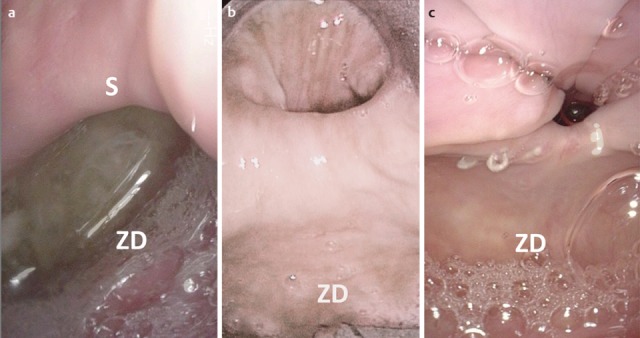
a – exposed entrance of Zenker’s diverticulum during flexible endoscopy; b – intraoperative view with diverticuloscope; c – 6 months after transoral endoscopic stapling, discrete residual diverticulum, the patient is free of complaints.

One of the organic disorders of the pharyngeal phase is a hypopharyngeal diverticulum (Zenker’s diverticulum). The pouch develops as a mucosal protrusion (false diverticulum) between the inferior horizontal and the superior diagonal part of the cricopharyngeal muscle (Figure 12 (Fig. 12)). This locus minoris resistentiae is called Killian’s Triangle. The diverticulum is mostly associated with a globus sensation as well as regurgitation of residues from the diverticulum. Complications of a hypopharyngeal diverticulum are aspiration pneumonia, perforations, and bleedings.

#### 3.2.3 Disorders of the esophageal phase

**Figure 13:**
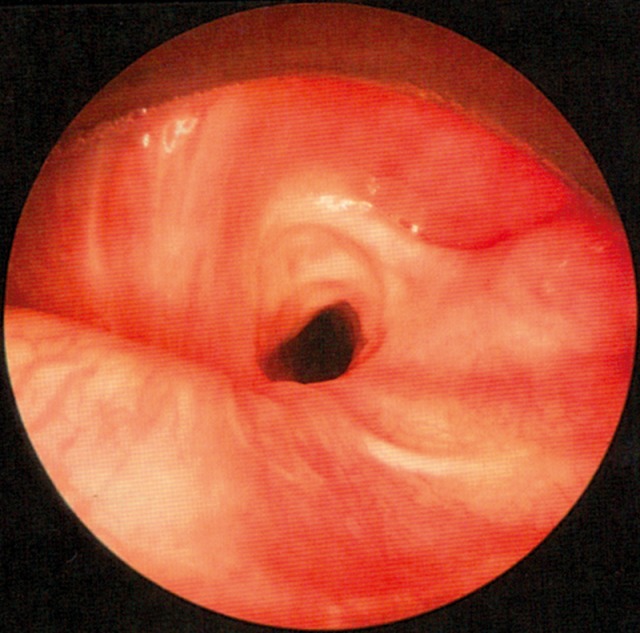
Ring-shaped scar causing stenosis of the distal esophagus

**Figure 14:**
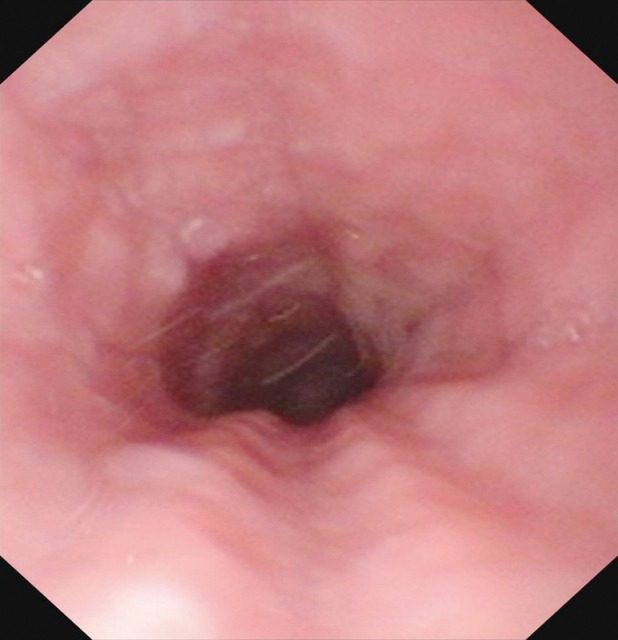
Eosinophilic esophagitis with persisting dysphagia. Condition after several esophagoscopies with removal of bolus. Increasing rigidity of the esophageal wall with problems of bolus transportation in the esophageal phase.

Inflammations of the esophagus such as, for example, eosinophilic esophagitis or inflammation caused by reflux disease, thrush or virus infections (*Herpes oesophagi*) do not impede transportation of the bolus, but under certain circumstances they may cause severe pain during the swallowing act (Figure 13 (Fig. 13), Figure 14 (Fig. 14)).

Besides motility disorders of the esophagus, probably caused by esophageal spasms, achalasia, Barrett’s esophagus, scleroderma, vasculitis, or collagenosis, diverticula and stenoses in particular may severely impair this phase.

Among tumors of the esophagus, adenocarcinomas and squamous cell carcinomas should be mentioned. Dysphagia is then a sequel of the tumor, surgical therapy, and/or radio- or radiochemotherapy.

### 3.3 Cervicogenic dysphagia

Cervicogenic dysphagia can be divided into three different groups:

1. Functional disorders of the cervical spine
2. Morphologic disorders of the cervical spine
3. Dysphagia after surgery of the cervical spine via an anterior approach

#### 3.3.1 Functional disorders of the cervical spine with dysphagia

Cervicogenic swallowing disorders and a foreign body sensation in the pharynx or throat, independent of food intake, are often elicited by functional reflex disorders of the cervical spine. Functional cervical spine disorders are almost exclusively located in the superior part of the cervical spine and the head joints. They develop either independently of pathomorphological disorders or as their consequence, and are usually completely reversible. In the case of persisting complaints, they may also merge into pathomorphological changes. 

Generally, C2/3 and C3/4 are the main segments responsible for functional swallowing disorders. The origin of functional cervical spine disorders are wrong movement patterns, degenerative changes or trauma, whiplash, and distortions. Even head injuries may lead to severe and persistent cervical spine disorders and swallowing disorders [44], [45].

Functional swallowing disorders can be associated with globus, lump, or foreign body sensation. Patients describe a sensation of swallowing “down a staircase”. This sensation may also be combined with a sensation of narrowness or stenosis in the throat. It is not possible to objectify an origin of the impaired swallowing. Psychological factors may also be responsible for the development and persistence of functional swallowing disorders. Seifert, however, describes premature conclusion as psychogenic “globus nervosus sive hystericus” as erroneous and therapeutically inefficient [46]. Furthermore, any organic origin, and especially in this context tumor disease, must be excluded through differential diagnostics.

Tension of the muscles inserting at the hyoid bone with pain on pressure in the sense of hyoid tendopathy can be proven by palpation. Diagnosis of cervical spine disorders is based on the methods and techniques of manual medicine relating to the cervical spine segments.

#### 3.3.2 Morphological origins of the cervical spine with dysphagia

The origin of morphological changes of the cervical spine may be inflammatory, degenerative, and traumatic processes and may affect patients beyond the age of 50.

In this context, changes as described by Schröter-Morasch which impair the form and function of the posterior pharyngeal wall are distinguished from those leading to stenosis of the spinal canal. The latter complaints may result in compression of the medulla and/or peripheral nerves with corresponding neurological symptoms and disorders of swallowing [47].

Degenerative changes of the skeleton like, for example, ankylosing spondylitis, arthritis deformans, or diffuse idiopathic skeletal hyperostosis as well as development of hereditary multiple exostoses can contribute to the formation of retropharyngeal masses due to hyperostosis. Asymptomatic cervical osteophytes, however, are also found in 20–30% of the population [48].

The flexibility of the pharyngeal tube is also responsible for the fact that deformities may be visible but not symptomatic. The swallowing disorder develops only when coordination disorders with impairment of bolus transportation occur due to reduced motility. According to Strasser et al. [49], dysphagia with aspiration is observed in patients who have cervical osteophytes.

The following aspects influence the swallowing process [47]:

1. Mechanical blockage of the bolus passage, especially for solid food
2. Impeded dorsal inclination of the epiglottis and thus impairment of complete closure of the laryngeal entrance
3. Impaired pharyngeal muscle contraction

Often, a foreign body sensation during swallowing is reported as a symptom of dysphagia. In such cases, dysphagia may also be associated with cranial or facial pains.

**Figure 15:**
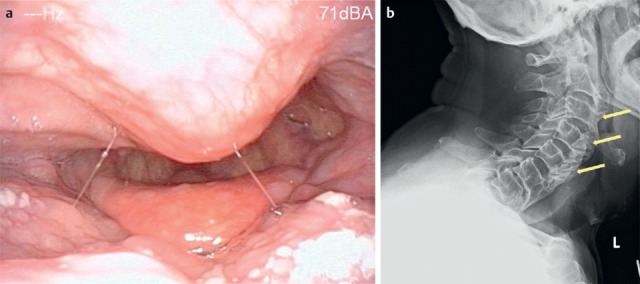
Forestier’s disease in a 75-year-old female patient. Dysphagia because of prominent hyperostosis of the cervical spine: a – endoscopically, a clear midline protrusion of the posterior pharyngeal wall is visible with relation to the base of tongue and epiglottis; b – lateral radiography of the cervical spine of the same patient with visible bony attachments to the vertebrae and ligamentous structures (C2–6, see arrows).

Bony-cartilaginous protrusions of the cervical spine or also ligamentous ossifications into the pharynx and/or cervical esophagus, as observed in diffuse idiopathic skeletal hyperostosis (M. Forestier) (Figure 15 (Fig. 15)), are mechanical obstacles that may impair the swallowing act. The prominent osteophytes in the area of the vertebrae C2–4 are regularly apparent in the context of rigid and flexible endoscopy. Because of the insufficient closure of the laryngeal entrance due to the prominent osteophytes of the vertebrae C3/4 and C4/5, an intradeglutitive aspiration of liquids may result. Computed tomography or radiography of the cervical spine can confirm the diagnosis. Differential diagnosis must always exclude a tumor disease.

Furthermore, fibrosis or also spasms may favor dysphagia in the case of contact with the bolus in the area of the osteophytes.

Seidler et al. mention 4 reasons for dysphagia in hyperostosis [50]:

1. Incomplete protection of the upper airways due to impaired mobility of the epiglottis; large osteophytes can block the downward movement of the epiglottis.
2. Incomplete glottic closure when swallowing due to osteophytes impeding the adduction of the arytenoids and thus of the vocal folds
3. Impaired elevation and anterior movement of the larynx
4. Mechanical impairment of the bolus transportation because of protrusion of the posterior hypopharyngeal wall

#### 3.3.3 Postoperative dysphagia after surgery of the cervical spine

**Figure 16:**
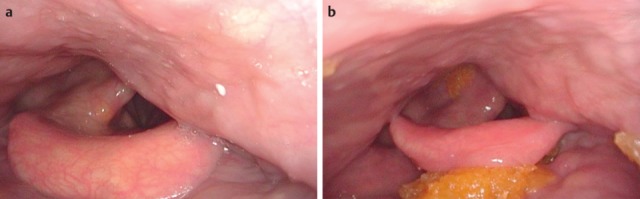
53-year-old patient with stiffening of the cervical spine and revision surgery, persisting dysphagia: a – the left lateral pharyngeal wall protrudes over the larynx with contact to the epiglottis; b – small bolus in the vallecula (physiological), transportation disorder of the bolus (carrots) in the right hypopharynx.

Surgery of the cervical spine in cases of trauma, decompression with discectomies or vertebral fusions may lead to an impaired pharyngeal phase of the swallowing act. This phenomenon is mainly observed after surgery of the cervical spine via an anterior approach. Fountas mentions postoperative dysphagia as the most frequently occurring complication with 9.5% in 1,015 patients after this surgical standard procedure [51]. According to Mukherjee [52], the etiology is most likely a multifactorial one. Dysphagia is elicited by pharyngo-esophageal denervation, swelling of cervical soft parts, scarring, and pressure damage [53] (Figure 16 (Fig. 16)).

In the first postoperative week, postoperative dysphagia after anterior cervical spine surgery is observed in up to 79%, after one month in up to 50–56%, and after one year in 13–21% [54], [55]. Risk factors are: high age, female gender, and multilevel surgery [55], [56]. Bartholome and Schröter-Morasch recommend intensive postoperative care for such patients in order to be able, at a timely stage, to introduce further functional or surgical therapy based on clinical, endoscopic, and radiological examinations [47].

### 3.4 Dysphagia caused by hypersalivation or xerostomia

Hypersalivation is triggered by the following factors:

- Central-neurological disorders (amyotrophic lateral sclerosis, Parkinson’s disease, apoplexy, infantile cerebral paresis)
- Peripheral disorders of the nerves V and VII
- Orofacial dysfunction
- Gastro-esophageal reflux
- Tumor surgery/iatrogenic salivary fistulas
- Adverse effects of medication (anti-neuroepileptic drugs, haloperidol)
- Mouthwash, tooth paste
- Idiopathic

Xerostomia may have the following origins:

- Central origins: Parkinson’s disease, depression, psychological factors, head injury, tumors of the central nervous system
- Adverse effects of medication: tricyclic antidepressants, sedatives and tranquilizers, antihistamines, antihypertensive drugs, chemotherapeutics
- Diabetes mellitus, exsiccosis
- Irradiation (>25 Gy, irreversible damage)
- Sarcoidosis, amyloidosis
- Pediatric patients: cystic fibrosis
- Resection of the salivary glands

### 3.5 Neurological diseases

For patients with dysphagia due to neurological disease, an interdisciplinary cooperation is always required in order to define conservative or surgical therapy based on the symptoms and prognosis. The following groups could be identified [57]:

- Neurovascular diseases (e.g. ischemic stroke)
- Neurodegenerative diseases (e.g. Parkinson’s disease)
- Neuromuscular diseases (e.g. amyotrophic lateral sclerosis, polymyositis)
- Neuro-traumatological diseases (e.g. head injuries)
- Neuro-oncological diseases (e.g. gliomas, para-neoplastic diseases)
- Neuro-infectiological diseases (e.g. brain stem encephalitis)
- Age-related changes of the swallowing function (presbyphagia)

### 3.6 Drug-induced dysphagia

Because of their pharmacological profile, nearly all drugs generally have the potential to induce or increase dysphagia. Factors like reduced general condition, high age, multiple medication, anatomical particularities of the gastro-intestinal tract, degenerative cerebral changes, and/or psychiatric anomalies are risk factors [1]. Overdosage of unnoticed reduced liver and renal performance with cumulative effects may also initiate or enhance drug-related swallowing disorders. Furthermore, there may be an interrelation of different drug substances having cumulative effects [58].

Drugs may cause a *direct* impact on the swallowing function when they have an effect on structures that directly contribute to the swallowing process like, for example, the muscles of the esophagus. They have an *indirect* effect when they influence the preconditions of the swallowing act such as, for example, drug-induced xerostomia [59].

Drug-associated influences on the swallowing function are often not sufficiently noticed, implicitly accepted, or remain unidentified [60]. Drug effects on the swallowing act are particularly crucial when anatomical functional changes such as, for example, chronic esophagitis or esophageal stricture [61] are present, when different medication is applied simultaneously, and/or when swallowing problems have already been known for a longer period of time.

#### 3.6.1 Oral medication-induced esophageal injury

Drug substances can lead to local inflammations and ulcerations through direct contact with the esophageal mucosa during swallowing. They are summarized as an independent symptom complex referred to as oral medication-induced esophageal injury (OMIEI) or drug-induced esophageal injury (DIEI). The major symptoms are dysphagia, sometimes foreign body sensation, globus sensation, and odynophagia. In many cases, esophageal transportation disorder is reported. Instinctively, patients drink more when swallowing solid consistencies. OMIEI is mostly to be expected in elderly patients, patients with reduced general condition, and patients with motility disorders or anatomical changes of the esophagus [59]. An overview of drugs causing predominantly OMIEI is given in Table 5 (Tab. 5).

Not only the substance, but also the type and size of the tablets and the used quantity of liquids have an impact on OMIEI [62]. Furthermore, a latency may be observed between the time of first drug intake and the occurrence of OMIEI [63].

#### 3.6.2 Systemic effect of medication on swallowing (classification)

##### 3.6.2.1 Centrally sedating/mind-altering substances

One important group consists of substances that purposely reduce central irritability and vigilance. Among these are anticonvulsive agents but also many antidepressants. In addition, antiallergic drugs as well as analgesics, especially with effects similar to opiates due to sedating components with impact on reflux, sensor function, and muscular coordination, may negatively influence swallowing functions. In the context of benzodiazepines, an effect on the laryngeal swallowing activity is assumed [64]. Regarding the treatment of pediatric epilepsy with nitrazepam, muscular coordination disorders of the cricopharyngeal region with aspiration and with lethal outcome were described [65]. Table 6 (Tab. 6) summarizes the most important drugs that may induce or enhance dysphagia based on their central effect. 

##### 3.6.2.2 Centrally effective drugs with peripheral side effect

###### Xerostomia 

Xerostomia is a predominantly peripheral side effect of centrally effective drugs. Among these are tricyclic antidepressants (e.g. amitriptyline), serotonin reuptake inhibitors [1], and opiate-containing analgesics [66]. Since aging is always associated with changes of saliva consistency and saliva production is reduced, drug-induced xerostomia may have considerable consequences, especially on bolus transportation [67]. Table 7 (Tab. 7) shows a list of drugs that typically induce xerostomia.

Often, the application of ACE inhibitors is typically associated with the side effect of chronic coughing which may have an indirect impact on the swallowing profile or even mimic aspiration. ACE inhibitors, however, are the only drugs that can evidently improve the swallowing function. Arai et al. described a reduced risk for aspiration pneumonia in stroke patients so that, even in the case of patients with normal blood pressure, ACE treatment was recommended [68]. 

###### Neuromuscular effect 

Neuroleptics reduce coordination and muscle activity of the pharynx and esophagus and may induce dyskinesia with an uncoordinated swallowing process [69]. Single cases of aspirations with fatal outcome have been reported [1].

Dopamine antagonists, which are applied, for example, in Parkinson’s disease, may provoke late dyskinesia or even elicit Parkinson’s disease and negatively influence an already existing swallowing disorder [67], even if they are intended to improve muscular coordination. Drugs can induce muscular weakness and/or myositis (Table 8 (Tab. 8)). Among these, lipid reducers (statins) and colchicine in particular should be mentioned [70]. One risk which has been known for a very long time and which occurs with high frequency is the drug-induced myopathy elicited by corticosteroids (steroid-induced myopathy) [71]. 

Drugs or substances that have an effect based on their form as injections or local applications are, for example, botulinum toxins, injection of which in cases of spasmodic dysphonia or cervical dystonia may cause swallowing difficulties [72]. Furthermore, superficial anesthetics (contained in some pastilles) reduce sore throat and pains of aphthosis. These are also applied prior to endoscopy of the upper aerodigestive tract for reversible reduction of the superficial sensitivity. 

### 3.7 Pediatric dysphagia

An age-appropriate development of sensory and motor functions is the precondition for normal swallowing ability. Table 9 (Tab. 9) shows a synopsis of gross motor and orofacial development [73].

#### 3.7.1 Origins of pediatric dysphagia

**Figure 17:**
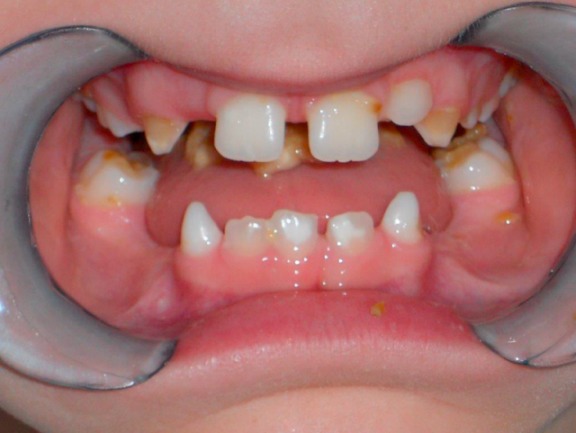
Dysphagia with disorders of the oral phase. Parts of the bolus are transported into the oral vestibule in an anterior and lateral direction because of a protrusion of the tongue. Malocclusion, dental enamel defects.

**Figure 18:**
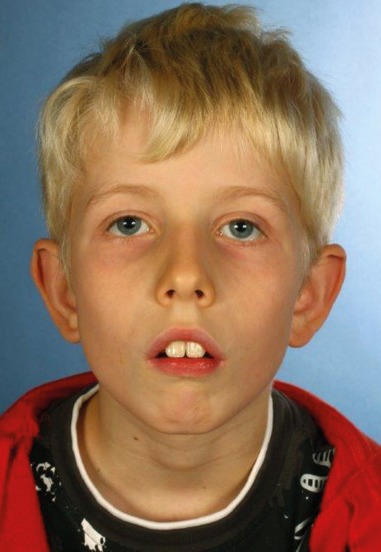
Adenoid face with orofacial dysfunction (myofunctional syndrome) with reduced orofacial tonus. As a consequence, open mouth, compensatory tension of the mental muscle, tired expression.

Pediatric dysphagia may have neurological, structural, functional, or behavioral origins [74], [75] (Figure 17 (Fig. 17), Figure 18 (Fig. 18)). Among these, traumatic brain damage and bleeds should be mentioned, along with neuromuscular disorders (infantile cerebral paresis, spinal muscle atrophy type II) and sequelae of premature delivery (coordination disorder of swallowing and breathing function with sensory-motor deficits, bronchopulmonary dysplasia, apnea bradycardia syndrome, paresis of the vocal folds after heart surgery, laryngomalacia, necrotizing enterocolitis after antibiotic therapy) [2]. Gastro-esophageal reflux [76] as well as acute and chronic, often immune-mediated mucositis (e.g. eosinophilic esophagitis, Crohn’s disease) complicate bolus transportation [77], [78], [79] and are associated with coughing, pneumonia, apnea, eating disorders, and failure to thrive. Even OMIEI can already be observed in children, especially when chronic diseases have been treated over a long period of time with oral drug intake (e.g. juvenile rheumatoid arthritis) or chemotherapy in cases of malignomas. Often, OMIEI and associated unspecific behavioral changes with selective/insufficient eating behavior is misinterpreted as a character-related particularity. Failure to thrive and recurrent vomiting are sometimes the only signs [79].

Furthermore, cranio-facial malformations (CHARGE syndrome: coloboma, heart anomalies, atresia of the choanae, retarded growth, genital hypoplasia, ear anomalies), Pierre Robin syndrome (cleft formations), anatomical particularities of the entire swallowing pathway (e.g. velum cleft, esophageal stenosis), and the consequences of tracheostomies may cause swallowing disorders in pediatric and adolescent patients. Premature infants, even without cerebral complications, are at an increased risk of developing dysphagia, even beyond the age of small children [80]. In general, children with delayed development show lifelong masticatory and swallowing difficulties that cannot ensure sufficient oral food intake.

Dysphagia must be distinguished from eating, feeding, and interaction disorders [81], while especially in infants and small children this idiosyncratic aspect does not meet the complexity of the disorder and a swallowing disorder may even be a symptom of a feeding disorder [82]. The attitude of considering “feeding disorders” and “dysphagia” as separated entities is currently being revised in favor of “swallowing and feeding disorders in developmental disabilities” (SFD-DD), which summarizes dysphagic symptoms with problems of the single swallowing phases and feeding disorders with problems of eating.

The problem of dysphagia is the result of persistent primitive reflexes that inhibit the development of physiological swallowing patterns. Ineffective orofacial coordination and impaired pharyngeal motility as well as insufficient triggering of the swallowing reflex lead to a quantitatively and qualitatively insufficient swallowing activity [83]. A typical symptom of pre-, peri-, or postnatal brain damage is infantile cerebral paresis. It is observed especially in premature infants as well as after complications during birth and is characterized by a very variable appearance [84].

The incidence of swallowing disorders in children with cerebral paresis amounts to nearly 99% [85], [86], [87]. The prevalence of swallowing disorders in the group of patients with diplegia and hemiparesis amounts to 25–30%, while disorders of oral food intake are observed in up to 80% of the patients with tetra-paresis and extrapyramidal mobility disorders [82], [84]. In children with syndromes (e.g. trisomy 21, fetal alcohol disorder syndrome), swallowing disorders are present in more than 50% [88].

Table 10 (Tab. 10) gives an overview of the origins of pediatric swallowing disorders.

The consequences are multiple: impaired quality of life, dependence on feeding tubes and tracheal cannulas, aspiration and aspiration pneumonia [89], dehydration as well as malnutrition are all severe sequelae that lead to developmental disorders, especially in maturing brains, and may even be life-threatening [90], [91]. Hence, the early diagnosis and introduction of therapeutic concepts in infants and children plays a major role.

#### 3.7.2 Symptoms/clinical aspects of pediatric dysphagia

Apparent signs of pediatric dysphagia are vomiting or regurgitation, coughing during or after food intake, and increased passage duration during the oral swallowing phase. Often, secondary hints are observed that indicate swallowing disorders such as lack of interest in eating, muscular tension during food intake, prolonged feeding times, spitting of food and liquids, gargling breathing sound or husky voice, breathing problems, and failure to thrive [73]. In cases of silent aspiration, visible symptoms are missing [92]. Chronic broncho-pulmonary diseases may be an indication of an unidentified pediatric swallowing disorder with aspiration. In cases of mild symptoms, such children are often erroneously treated with broncho-dilative or corticoid-containing drugs. In children with diseases of the neuro-muscular passage, various other basic diseases, and the side effects of medication, e.g. increasing fatigue during food intake, may enhance the risk of dysphagia.

#### 3.7.3 Feeding, screening, and clinical examination of swallowing in children

Clinical assessments are methods that do not show sufficient validity as a single diagnostic method regarding the prediction of aspiration in children and are thus considered as insufficient [93]. On an international level, only few English, standardized, and validated procedures exist; and only the “Dysphagia Disorders Survey” (DDS) and the “Schedule for Oral Motor Assessment” (SOMA) are considered to have sufficient clinical value [94]. No comparable, validated instruments exist in the German language. Transfer of screening procedures as performed on adults to pediatric patients is not allowed, if only because of the course and the quantities to be swallowed.

If the examination circumstances and the general condition of the pediatric patient are appropriate, feeding should be observed. In children who apparently aspirate anamnestically, this procedure must be omitted. As in the procedure with adults, in children several aspects are also assessed, such as vigilance, breathing, body tension, and especially head and trunk control. The findings must be related to the corresponding milestones of motor, cognitive, and orofacial development (including sucking, lip closure, control of jaw and tongue, masticatory movements, hand-mouth coordination, see Table 9 (Tab. 9)).

#### 3.7.4 Standardized procedure regarding anamnesis and examination

##### 3.7.4.1 Anamnesis in children

The following questions must be clarified with the reference persons (modified according to [95]):

- How long does it take to eat a meal (more than 30 minutes?)?
- Do you or the child consider meal times as stressful?
- Does the child show breathing problems during food intake?
- What about the weight development of the child?
- Has the child suffered from (aspiration) pneumonia?

In the anamnesis, questions are also asked about pregnancy, birth, general development (motor, swallowing, speech), allergies, medication, previous diseases, previous treatments and therapies. One main focus is placed on the current nutrition and eating situation. Also, oral habits such as the use of pacifiers, teeth grinding, and especially oral preferences must be assessed.

##### 3.7.4.2 Preparation of flexible endoscopic swallowing examination in children

Oxygen saturation is measured by pulse oximetry. The swallowing frequency is observed. One gulp per minute is normal; during endoscopy 3 times per minute [96]. The ENT specific findings of the head and neck region and the dental status are documented, while the focus is on nasal anatomy, peri- and enoral as well as oropharyngeal motor and sensory functions. After decongestion of the nasal mucosa with age-appropriately dosed nasal drops, mucosal anesthesia of the anterior nasal parts is performed with gel (e.g. Xylocain gel, 0.1 ml on each side). This measure may increase the cooperation of the child significantly and avoid traumatization regarding subsequent examinations. Mucosal anesthesia of the anterior nasal parts with gel does not influence swallowing ability [97]. Anesthetizing sprays should be avoided because they are painful and the anesthetized area cannot be well controlled. In cases of instable children with cardio-pulmonary diseases, the application of decongesting nasal drops and anesthetizing gel must be discussed with the treating pediatricians in the sense of risk evaluation. Sedation (e.g. with chloral hydrate) should not be performed. Although swallowing is a basic ability, it is to be expected in the examined patient population that even only a little sedation will negatively influence the examination result.

Besides the reference person, the presence of another person, e.g. the treating therapist, has turned out to be very useful and is also recommended by the American Speech-Language-Hearing Association (ASHA) [98]. In an interdisciplinary team, an individual treatment concept can be directly elaborated. Since parents often estimate the swallowing function of their children as being better that it actually is [86], [99], [100], it is recommended that additional information be acquired from therapists such as pediatricians, speech therapists, but also from educators and teachers.

Three different food textures are prepared and first a native swallow per consistency is performed. As crumbly food, homogenous bread without crust is appropriate. Viscous food is prepared with water and thickening agents, and for liquid, water is used. Of course, special preferences of the children can also be taken into consideration. If necessary, commercial food coloring can be used. This may have the advantage that even in the context of retrospective evaluation of the collected data, the color represents the examined consistency. Green and blue coloring provide a very good contrast to the mucosa. The fluids should be strongly colored so that they look dark in the cup, thus allowing even small residues to be identified. This is especially important in order not to overlook laryngeal penetration and possible moistening of the vocal folds.

Regarding methylene blue for bolus coloring for better visualization, reports in the USA stated that septic patients developed allergic reactions [96]. The suggestion was made to do without coloring, and, in cases of insufficient visibility, to mix the bolus with barium. Barium sticks strongly to the mucosa and the endoscope and thus prevents assessment of other boluses. If barium is aspirated, it may lead to pneumonia, so barium should not be applied. The use of jelly is not recommended if liquid can be aspirated because jelly turns to liquid when it is kept in the mouth for a longer time and the intended consistency is not monitored. Commercial thickening agents should be preferred (e.g. Nestargel, ThickenUp, Thick&Easy, Nutilis).

##### 3.7.4.3 Standardized performance of functional endoscopic swallowing examination in children

For the examination of dysphagia in children [73], video-fluoroscopy and functional endoscopic swallowing examination are generally available, as they are for adults. Both procedures are often applied in a complementary manner [101], [102]. In the context of video-fluoroscopic swallowing examination, the radiation exposure of the child is a clear disadvantage, especially if examinations have to be repeated. However, it is required when evidence-based results are expected and therapeutic success is to be evaluated. Data on radiation exposure and the effective dose were presented by Weir et al. [103]. In their report, the radiation dose was considered acceptable for older children; for younger children as being too high. In children with motor and cognitive impairment, it is better to perform a functional endoscopic swallowing examination. Considering that studies performed on children indicate a nearly 100% conformity of video-fluoroscopic and functional endoscopic swallowing examination [104], it seems reasonable, particularly in the case of children, to first perform regular functional endoscopic swallowing examinations and then video-fluoroscopic swallowing examination if findings or questions appear that have to be further clarified. Complications like hypersalivation, epistaxis as well as syncope due to vasovagal reflex or laryngeal spasm are described. These complications, however, are very rare, and the method is estimated as being safe, also in an outpatient context [105], [106]. Equipment with a pediatric emergency kit and regular courses on pediatric reanimation are recommended in all cases.

##### 3.7.4.4 Procedure

As with examinations of adults [101], an observation at rest (saliva, morphology, spontaneous movement, etc.) is performed along with functional examinations without food (phonation, swallowing of saliva, cleaning functions, etc.), functional examinations with food (different bolus volumes and consistencies), and an examination of swallowing techniques and cleaning maneuvers. It is recommended that a sheet be wrapped around the trunk and legs of the child, depending on the age, and that the child be placed on the reference person’s lap. The arms should be positioned near the body so that the child’s legs are between the legs of the reference person [73]. This position provides a sense of security and ensures a stable position during the examination. At the same time, uncontrolled movements of the arms are avoided. An additionally present person may support the child’s head from behind.

It is recommended to use an endoscope which ensures a good overview, on the one hand, and, on the other, only minimal irritation of the mucosa in order to avoid injuries and to keep the children cooperative. In premature babies or neonates, flexible endoscopes with a diameter of about 2 mm are appropriate. In addition to the assessment of anatomical morphological and functional findings of the naso-, oro-, and hypopharynx and the larynx (motility of the vocal folds), special attention should be paid to salivary residues or salivary aspiration as well as spontaneous cleaning mechanisms (harrumphing, coughing). If silent aspiration is found, the examination should be interrupted or further swallowing should be continued only very carefully and with small quantities of thickened food. In cases of residual saliva, the patient can be asked to cough, or a better overview can be achieved through transnasal or transoral suction. The different consistencies of “thickened”, “liquid”, and “crumbly” are mostly applied with a spoon that is appropriate to the child’s age, starting with low and, if possible, increasing quantities. The evaluation is performed based on the penetration-aspiration scale described by Rosenbek [107]. The examination is video-documented. A statement on the laryngeal sensitivity can be given by slight touching the laryngeal epiglottis surface near the tip and observing the reaction with coughing or choking, or by applying defined air jets [108].

If the child cries permanently, oral application of the substances is not possible because of the risk of aspiration and the probability of false-positive evaluation. If oral residues are present, they are removed after the examination.

Generally, the examiner tends to remove tracheal cannulas and feeding tubes prior to a functional endoscopic swallowing examination in order to ensure as undisturbed as possible laryngeal elevation, to reduce possible compression of the esophagus by the cuff, and to reduce the presumed laryngeal and hypopharyngeal mucosal irritation caused by the feeding tube. Studies in adults revealed that swallowing ability in the context of endoscopic evaluation is the same with and without a tracheal cannula [74], [92]. Tracheostomized children can be examined with or without a cannula; children with a naso-gastric feeding tube can be examined with or without the tube [109]. If there is even the slightest difficulty in removing the tracheal cannula beforehand, the examination should be performed with the cannula. Feeding tubes are generally left in place because changes of the results are not to be expected. If the situation occurs during the examination that laryngeal elevation seems to be relevantly impaired or that a reflux of the bolus is observed postdeglutitively, first the examination has to be performed with present cuff with unblocked cannula, and then, in the case of persistent postdeglutitive impaired passage, with the cannula removed.

## 4 Diagnostics of dysphagia

### 4.1 Anamnesis of dysphagia

The anamnesis provides hints on possible origins of the dysphagia, its clinical symptoms, and its consequences (see Table 11 (Tab. 11)). It can be obtained by questioning the patient, relatives, therapists, and nursing staff or by studying the patient’s file. Information on the following aspects is important:

- Basic major disease, time of first diagnosis
- Treatment up to now
  - In tumor patients: type and stage of the primary tumor and surgical, radiological, and chemotherapeutic intervention
  - In neurological patients: location and extent of the lesion, course, medication
- Nutrition in the course of the disease: parenteral, enteral via feeding tubes, oral with modification of food intake, of the consistency of food
- Signs of malnutrition and exsiccosis
- Respiratory status in the course of the disease: ventilation, tracheostomy, pulmonary complications
- Hints of reflux symptoms
- Drug intake (many drugs may cause or increase dysphagia, e.g. by xerostomia, extrapyramidal mobility disorders, gastrointestinal disorders, myopathy [70], [110], see chapter 3.6 on drug-induced dysphagia)
- Complaints when breathing, swallowing of saliva and secretion, eating, and drinking

Symptoms of dysphagia of the oral, pharyngeal, or esophageal phase can be assessed by means of a questionnaire [37]. According to Wright and Ellis [111], the subjective complaints of the patients and locations of the oropharyngeal dysphagia correlate strongly.

### 4.2 General assessment, clinical examination

The swallowing procedure is a highly complex movement of which disorders are generally caused by several factors. The pathophysiological causes must be sought by intensive medical examination of the head and neck region with evaluation of relevant single neurological functions in order to find the appropriate therapy. An orienting check of cerebral performance, ability to communicate, and overall motor functions (posture, control of head and torso, paresis, ataxia, hyperkinesia, dystonia; assessment of the cervical spine, head and neck muscles) are included among other aspects.

The diagnosis of whether it is the case of a peripheral neural lesion or supranuclear central damage is mainly based on an exact assessment of reflexes and voluntary mobility as well as muscle tonus (see Table 12 (Tab. 12)). Both types may occur at the lips, the tongue, the velum, and the larynx on one or both sides [112], [113].

The symptoms cannot always be delineated because, for example, damage of the brain stem affects the second peripheral as well as the first central motor neuron. During phases of regression, changing clinical aspects can be observed. 

#### 4.2.1 Examination of the lips, jaws, tongue, and anterior oral cavity

##### 4.2.1.1 Structures

The structures are assessed based on the form and symmetry of the lips and the tongue, on the jaw and dental status, and on the appearance of the mucosa. Patients after cancer treatment, for example, have massive structural deficits and dental defects. Extended scarring and edema may significantly impair head posture, motility of the floor of the mouth, and laryngeal elevation.

After intensive medical care, while no dentures are inserted, changes of the alveolar ridges already occur after a few weeks.

##### 4.2.1.2 Motility

The examination of the motility is first performed by asking the patient to make voluntary movements:

- Lips: closing, pursing, spreading
- Jaw: opening, closing, lateral shifting, rotating
- Tongue: sticking out, lifting, moving backward, circling, moving along the lips, pressing the tip of the tongue into the buccal pouch

According to a study performed by Leder in more than 3,900 patients, reduced motility of the tongue is a risk factor for aspiration, independent of the basic disease [114].

Muscle tonus impairs motility if it is unbalanced (too high or too low). The mandible may drop because of reduced tonus of the masticatory muscles or – a frequent symptom after head injury – because of too high tension of the muscles lowering and retracting the mandible. Closure of the lips may be insufficient due to too high (retraction of the lips) or too low tension while the range of motion is not necessarily impaired.

##### 4.2.1.3 Evaluation of the oral sensitivity

In cases of reduced sensitivity, there is always the risk of limited bolus control. Often, patients with disorders of tactile sensitivity complain that they bite their tongue and the inward side of the cheek and that this is very painful. Tactile sensitivity is examined by touching with cotton swabs.

Perception of the temperature in the oral cavity is only checked in an orienting way by touching with a small laryngeal speculum that was put either in cold or warm water. Determination of thresholds is methodically very complex and its clinical relevance is a subject of discussion.

#### 4.2.2 Examination of the posterior oral cavity, velum, and pharynx

##### 4.2.2.1 Oral cavity

In the posterior part of the oral cavity, special attention must be paid to swellings, substantial defects, and scars, especially in patients who have undergone tumor therapy of the tongue, the palate, the pharynx, and the larynx. Protrusions of the posterior pharyngeal wall must be clarified with regard to changes of the cervical spine, sometimes also of inflammations and abscesses.

##### 4.2.2.2 Velum

Too high tension of the muscles of the palatine arch give a sharp contour to the palatine arch and create a relevant distance between the velum and the posterior pharyngeal wall. Insufficient velar tonus can be seen by ventral advancing of the velum during forced expiration (“fluttering in the airflow”).

A central paresis is diagnosed when voluntary phonation of [a:] lasting for seconds and repeated phonation of [a] does not cause lifting of the velum but a reflex elevation when attempting to trigger the palatal or gag reflex, e.g. laughing, crying, or yawning. If the gag reflex cannot be triggered (a frequent symptom, for example, after head injury), examination with the flexible endoscope is possible to see if lifting of the velum and a nasopharyngeal closure occurs during the swallowing act. If this phenomenon is not observed, peripheral paresis can be expected.

##### 4.2.2.3 Pharynx

The contraction of the pharyngeal muscles is examined at the same time, during voluntary phonation and when triggering the gag reflex. However, it is not possible to exactly evaluate whether the contraction is normal or impaired. This aspect can only be assessed by video-endoscopic, radiological, or manometric findings.

##### 4.2.2.4 Sensitivity and possibility of triggering a reflex in the oropharynx 

Disorders of sensitivity are found as already described:

- After tumor surgery – lesions of peripheral nerves in the area of scars of the pharynx and larynx or in the area of flap transplantations
- After radiation
- After a stroke – the base of tongue, palatal arches, velum, and posterior pharyngeal wall are mostly affected unilaterally.

Recent examinations revealed that in cases of unilateral cerebral infarction, disorders of the sensitivity of the palatal arches may be associated with prolonged latency of the swallowing reflex and aspirations [115], [116]. Hence, an important role of the swallowing cortex seems to be to secure intact sensitivity of the oral cavity and to coordinate the oral with the pharyngeal phase in such a way that no early passage of swallowed material and no aspiration occur [101]. The possibility of triggering the gag reflex is individually very different. If it is missing, there seems to be an increased risk of the presence of dysphagia [117], although an existing gag reflex does not automatically allow the conclusion to be drawn of an undisturbed swallowing procedure.

There are also people who do not have a gag reflex. When the lips already touch a straw, the expectation of liquid or food triggers hypoesthesia of the pharynx and larynx. Thus it is possible that during swallowing, for example, the laryngeal epiglottis surface may be touched.

### 4.3 Questionnaire assessment, screening procedures, and clinical diagnostics of dysphagia

In the clinical routine, the equipment for swallowing examinations is not always available, so structured questionnaires and screening procedures are often used first in order to make decisions on further diagnostic and therapeutic procedures. Clinical swallowing examinations are usually preceded by instrument-based diagnostics and are mostly performed by speech therapists. Up to now, no guidelines exist on all mentioned procedures.

#### 4.3.1 Questionnaire assessment

The present assessments focus either on “symptoms of dysphagia” or “quality of life”. The former are more appropriate for obtaining information on dysphagia-specific symptoms; the latter are better for assessing the overall situation of the patients with their dysphagia-related impairments. In this way, they meet the perspective of diseases according to the “International Classification of Functioning, Disability, and Health” of the World Health Organization.

Other parts of symptom-specific assessment are the 10-item assessment tool (EAT-10) [118], [119], the Sydney Swallowing Questionnaire (SSQ) [120], [121], and the Munich Dysphagia Test – Parkinson’s Disease (MDT-PD) [122].

To evaluate the areas related to quality of life and its impairment, the MD Anderson Dysphagia Inventory (MDADI) [123], the Swal-QOL and the Swal-Care [124], [125], [126], the Dysphagia Handicap Index (DHI) [127], the Performance Status Scale for Head and Neck Cancer Patients (PSS-HN) [128], [129] as well as the Functional Assessment of Cancer Therapy – Head and Neck Scale (FACT-H&N) [128] are applied.

#### 4.3.2 Screening procedures

Screening procedures are useful instruments to identify patients who are possibly at risk and to estimate their risk without taking too much time [130] in order to initiate further diagnostics. They should be quick, simple, safe, significant, and associated with little stress for the patient in the sense of bedside examination that could also be performed by staff members who have received special training. For first rapid decisions, swallowing disorders should be found with a sensitivity (clear evidence) and specificity (clear exclusion) of 70% each [131]. A gold standard for current screening procedures does not yet exist. Nonetheless, screening procedures have a high significance because availability of instrument-based swallowing diagnostics (functional endoscopic and video-fluoroscopic swallowing examination) is not given everywhere and at short notice, but an immediate, optimally reliable estimation of the nutrition of the patient has to be made. More than 50% of the patients who aspirate do not cough. So in the clinical diagnostics, a high percentage of silent aspiration, which is a limiting factor of sensitivity, remains unidentified.

Contraindications of an aspiration quick test are:

- Already known signs of aspiration
- Pulmonary pathologies
- Severe disturbed consciousness

For the screening of neurogenic dysphagia, the timed test [132] is a valid instrument. 150 ml of cold still water must be drunk as fast as possible from a glass. Time, swallowing frequency, remaining quantity in the glass as well as coughing and quality of voice after drinking are documented. The data refers to ml/s and the mean volume per mouthful (ml). A swallowing rate of <10 ml/s is considered to be pathological. For the item of swallowing frequency, a sensitivity of 96% and a specificity of 69% is given. The authors mention that the test is not appropriate for patients with severe swallowing disorder and apparent aspiration.

In the acute phase of a stroke, well-trained caring staff have at their disposal the practicable Standardized Swallowing Assessment (SSA) [133], [134], the Gugging Dysphagia Bedside Screening [135], or the Daniels Test, which is performed by speech therapists [13]. The last-mentioned test is expected to predict aspiration with a sensitivity of 92% and a specificity of 67% based on the 6 clinical symptoms of dysphonia, dysarthria, impaired voluntary coughing, reduced or missing gag reflex, coughing, and change of the quality of the voice after swallowing water. Another test to be applied is the 3-Ounce Water Swallow Test described by Suiter and Leder [136]. This test is positive when coughing, attack of suffocation, or a damp voice occur and the test has to be interrupted. Because of the high quantity of water, the test should only be performed after sufficient testing with lower quantities.

Aspiration quick tests are appropriate for reaching a decision on urgent measures because of the shorter duration and the lower costs. In cases of anamnestically or clinically expected aspiration/dysphagia, the first examination is the functional endoscopic swallowing examination. After this examination, all further steps regarding further examination, intensive clinical and radiological diagnostics and their order can be planned.

For dysphagia after a stroke, numerous different tests exist like, for example, the 50 ml water test [47], the 3-Ounce Water Test (=90 ml water swallow test) [137], the Kidd Water Test [138], the Burke Dysphagia Screening Test [139], the water test according to Daniels [13], the Bedside Swallowing Assessment (BSA) [140], the Standardized Swallowing Assessment (SSA) [133], [134], the Massey Bedside Swallowing Screening [141], the Nishiwaki Score [142], the Gugging Swallowing Screening [135], the Volume-Viscosity Swallow Test (V-VST) [143], the Toronto Bedside Swallowing Screening (TOR-BSST) [144], the Modified Mann Assessment of Swallowing Ability (MMASA) [145], the Acute Stroke Dysphagia Screen [146] as well as the Cough Test [147]. 

A relatively high sensitivity/specificity and a relatively easy practicability are achieved by the 90 ml water test [136], [137] and the Gugging Swallowing Screen (GUSS, evaluation of different consistencies) [135].

##### 4.3.2.1 90 ml water test (3-Ounce Water Swallow Test)

The patient is asked to drink 90 ml of water from a glass with or without straw without interruption. Hints of aspiration and interruption criteria are the following:

- It is not possible to drink the whole quantity of water.
- Coughing or attack of suffocation occur up to 1 minute after the end of the test.
- Gargling, damp quality of the voice

Since a quantity of 90 ml may be risky for the lungs, pre-tests with smaller water quantities (1, 3, 5, 10 ml) are useful. If no symptoms become apparent, most probably no aspiration of liquid consistencies is to be found. Because of the low specificity, many patients are not at risk of aspiration despite suspect results. In any case, any hint of aspiration should lead to further diagnostics. 

##### 4.3.2.2 Gugging Swallowing Screen (GUSS)

The preliminary examination or the indirect swallowing test include verification of vigilance (the patients must be able to stay awake for at least 15 minutes), ability to swallow saliva, to harrumph twice voluntarily, and/or to cough, as well as assessment of drooling and changes of the voice during this time.

In the direct swallowing test, the consistencies of pulpy, liquid, and solid are examined.

Dysphagia is present when at least one of the following symptoms is observed:

- Swallowing is not possible.
- Delayed initiation of swallowing (liquid >2 s, solid food >10 s)
- Drooling
- Involuntary coughing before, during, or after swallowing (up to 3 minutes later)
- Change of the voice after swallowing (the patient is asked to pronounce [o] before and after swallowing)

Based on a scale of 20 points, 4 severity degrees are distinguished.

The test assesses the severity of dysphagia as well as the risk of aspiration with a sensitivity of 100%. With a specificity of 50–69%, a high rate of false-positive results can be expected. Consequently, endoscopic swallowing diagnostics are recommended in cases of hints of low-grade dysphagia with a low risk of aspiration. A protocol form with evaluation can be retrieved on the internet [148].

In order to assess the ability to swallow after tumor resection of head and neck tumors, the Frankfurt Dysphagia Screening (Fra-DySc) [149], the Water Swallow Screening Test [150], and the Mann Assessment of Swallowing Ability – Cancer (MASA-C) [151] are described. Some of these screening tests are not validated or not sufficiently significant. Thus, the swallowing function of tumor patients with suspected dysphagia should always be examined via endoscopy [152].

#### 4.3.3 Examination of patients with tracheal cannulas

In patients with tracheal cannulas, aspirated material can be relatively easily suctioned and identified as such after staining [153] or detected by means of the glucose oxidase test [154].

A positive result always indicates aspiration. A negative result, however, does not allow a conclusion to be drawn because low quantities of aspirated material are often not assessed.

For staining, blue food coloring is useful. Because of possible toxicity, the application of methylene blue should be avoided [96], [155].

### 4.4 Clinical procedures of swallowing examination

The term clinical swallowing examination summarizes an examination procedure which includes, in addition to the specific anamnesis of swallowing and the assessment of the vigilance and compliance of the patient, examination of all structures relevant for swallowing as well as the brain nerves involved and the performance of swallowing tests in the context of screening procedures [47], [156], [157], [158]. So far there is no standardized procedure [16], [159] and no sufficiently validated procedure is available. Particularly profound knowledge of the functionality of tracheal cannulas is extremely important in this context. For evaluation of the results of the clinical swallowing examinations, the Nishiwaki Score [142] and the Cologne System for Swallowing Examinations (Kölner Befundsystem für Schluckuntersuchungen) [160] are applied, for example.

#### 4.4.1 Gradual approach

In the German language, the NOD approach for patients with neurogenic oropharyngeal dysphagia [161] and the concept according to Stix for geriatric patients [162] are described as gradual approaches. These concepts are based on different methods of anamnesis, screening, clinical swallowing examinations, and instrument-based swallowing examinations. The aim of these gradual approaches is to clarify responsibilities, to identify patients with swallowing disorders at an early stage by means of structured procedures, and to introduce appropriate therapies based on a consistent terminology and defined criteria.

#### 4.4.2 Endoscopic examination techniques

Video-endoscopy is an established tool for the efficient assessment of swallowing disorders [2], [163], [164], [165], [166] and it is considered nowadays as an essential basic examination. It only stresses the patient to a low extent.

Regarding the assessment of residual findings, penetration, and aspiration, it is equivalent to radiological video-fluoroscopy [25], [28], [163], [165], [167], [168], [169], [170], [171], [172], [173], [174], [175], [176] and is thus of high clinical relevance. The number of aspiration pneumonias in an interval of 6 months was significantly reduced to 0 after the introduction of endoscopic swallowing examination in the management of dysphagia [177].

The objectives of endoscopic examination are:

- Diagnostics and delineation of structural and neurological disorders and their characteristics
- Decision on whether food/liquid intake is sufficient in a reasonable time in a natural way or whether feeding tubes or parenteral nutrition must be discussed
- Decision on whether protective measures for the deep airways are required (intubation, tracheostomy)
- Presence of an indication of saliva reduction
- Decision support for swallowing attempts with food, nutrition development, unblocking/decannulation
- Verification of the effectiveness of posture changes, modifications of food/liquid
- Indication for further diagnostic measures (video-fluoroscopy, pH-metry, manometry)
- Indication for functional therapy of dysphagia and its evaluation
- Indication for prosthesis and surgical therapy

The following methods are available for video-endoscopic examination:

- Transoral examination (**T**rans**o**ral **e**valuation of **s**wallowing = TOES) with rigid laryngoscope [166]
- Transnasal examination with a flexible endoscope as standard procedure [2], [27], [164]
- Transstomal “retrograde” examination in patients with tracheostoma

Each method has its advantages and limitations. The principles of the examination and the assessment criteria are always the same.

##### 4.4.2.1 Evaluation of swallowing (TOES) by means of rigid endoscopy

Laryngoscopic examination is performed with 70° or 90° laryngoscopes for assessment of the hypopharynx and larynx and the functions relevant for swallowing (see below).

After removal of the laryngoscope, the patient receives appropriate material to swallow. Immediately afterwards, laryngoscopy is performed again. After modification of the food and/or the performance of cleaning and swallowing techniques, the patients has to undergo endoscopy. This clearly implies that a high degree of readiness to cooperate is necessary.

The advantages of this examination are:

- After intensive medical treatment with nasal intubation, gastric tube, suction maneuvers, some patients fear having “a tube through the nose again” and would rather tolerate laryngoscopy than transnasal examination.
- During swallowing there is no interference caused by the endoscope.
- There is no time limit. Therapeutic interventions as mentioned above (change of food consistency, posture, swallowing mode, cleaning techniques) can be repeated as often as needed.
- During endoscopy, the examiner holds the tongue and can thus more easily control the patient’s head, which may be stabilizing in patients with impaired motor function.
- The mentioned risks of transnasal examination are avoided (see below).

The disadvantages of this examination are that the following phases cannot be observed:

- Early passage of the material from the oral cavity into the pharynx or larynx (leaking, pre-deglutitive aspiration)
- Initial closure function of the larynx
- Re-elevation of the epiglottis after dorsal flexion during the swallowing reflex

Mucosal anesthesia is obsolete because it would impair triggering of the swallowing reflex. So the patient may be unwilling to undergo the examination because of the gag reflex. The majority of patients suffering from dysphagia, however, show significant sensitivity disorders in the pharynx. 

Even if the evaluation of the swallowing function in transoral examinations is only possible as an “indirect assessment”, i.e. post-deglutitive, residues, penetration, and aspiration [29], [176] as well as effects of therapeutic interventions and evaluations can be revealed in a similar way as in transnasal examinations. 

##### 4.4.2.2 Flexible video-endoscopy of swallowing

The principles of examination of the pharyngeal swallowing function by means of flexible and video-endoscopy were described by Langmore et al. [2] in 1988 as well as by Bastian [164] in 1991 with the term “flexible endoscopic evaluation of swallowing (FEES)”. In the following years, they were methodically extended or varied. The different possibilities are:

- Flexible endoscopic swallowing examination as standard procedure: flexible rhino-pharyngo-laryngoscope, 2.3 or 3.5 mm in diameter
- Flexible endoscopic evaluation of swallowing with sensory testing (FEESST) [178]: flexible rhino-pharyngo-laryngoscope with an additional channel for issuing a defined air flow for sensitivity testing
- Fiberscope (bronchoscope) with suction channel for removal of secretion and non-swallowed material as well as for release of liquid; appropriate for sensitivity testing as well as for swallowing abilities; 4.5 mm in diameter [179], [180], [181]
- Transnasal video-panendoscopy (ViP) for further questions [182], see chapter 4.7 and video at https://www.thieme-connect.de/products/ejournals/html/10.1055/s-0035-1545298#N71110.

###### Flexible endoscopic swallowing examination

The swallowing examination is performed with the flexible rhino-pharyngo-laryngoscope. The width of the nasal cavity is revealed by anterior rhinoscopy. Sometimes decongestion of the mucosa may be indicated. Local mucosal anesthesia by means of Xylocain gel reduces the sense of pressure or the risk of triggering pain and improves the sliding capacity of the endoscope. However, it may also lead to hypersalivation, disturbed sensitivity, and changes of the swallowing procedure.

It is inserted on the nasal floor and guided through the lower or middle nasal meatus. For observation of the velar elevation, the tip of the endoscope is placed at the border between the hard and soft palate.

For observation of the vallecula, the base of the tongue, the pharynx, and the larynx, the endoscope is positioned at the level of the palatal arches directly below the uvula (“superior position” or “swallow position”, corresponding to position 1 in ViP). The position of the tip of the endoscope should remain at this level during the whole swallowing reflex in order not to impair laryngeal movements.

For careful examination of the laryngeal entrance, the vocal folds and the trachea, the tip of the endoscope can be moved behind the cranial epiglottis edge (“inferior position” or “postswallow position”, corresponding to position 2 in ViP) [28], [169]. After the swallowing procedure, this position is recommended for assessment of even low quantities of possibly penetrating material and for identification of aspirated material in the glottis and trachea (cave: vasovagal reflex), see also chapter on video-panendoscopy and NBI. 

If the patient is tracheostomized, the glottis can be examined by means of retrograde laryngoscopy after removal of the cannula. In this way, tracheostoma and suprastomal trachea can be assessed. Spontaneous aspirations of saliva, but also aspirations after application of (colored) food and liquid, are found.

The advantages of transnasal examination are:

- It can be applied in patients with impaired consciousness or missing head control / torso stability, e.g. in intensive care units (see chapter 3.7) [183], [184].
- It is appropriate for children [104], [108], [185], [186] and patients with limited ability to cooperate such as patients suffering from dementia [179], [187].
- Structures and single functions can be judged that can only be assessed with difficulty, e.g. inflammations, swelling of soft parts, defects, scars (especially after tumor therapy) [188], changes of tension, or discrete mobility disorders.
- Aspirations of saliva can be detected that do not occur during swallowing of test substances, e.g. contrast media [23], [24], [25], [169], [176].
- It can be repeated as often as required and is thus excellent for evaluation of therapies.
- No radiation exposure occurs.
- The examination is possible “on site”, e.g. in all departments in emergency hospitals or rehabilitation institutions as well as in retirement and nursing homes [189], [190], [191].
- Use of bio-feedback procedures [192], [193]
- Lower costs than radiological diagnostics

The disadvantages are:

- The oral preparatory phase can only be observed in endoscopy with retrospection.
- During the pharyngeal phase, the base of the tongue, pharyngeal and laryngeal structures can only be assessed directly before and after the pharyngeal phase; during reflex swallowing, the optic mostly comes in contact with the posterior pharyngeal wall due to the correlated effect of the elevating velum and the contracting pharynx. In flexible endoscopy, white out [157] inhibits an adequate evaluation of the crucial components of laryngeal closure and opening of the esophageal sphincter.
- No assessment of the esophageal functions
- No assessment of the quantity of aspirated material
- In patients with significant disorders of motor function such as pareses, ataxia, tremor, or dystonia, it may be difficult to position the endoscope and to keep it still during the swallowing maneuver [186].
- Cave: vasovagal reflex with hypotonia and bradycardia, especially when touching the epiglottis with the tip of the endoscope, or laryngeal spasm when touching the false vocal folds / vocal folds while the larynx elevates during swallowing. Because of the rare occurrence of such events, this method is considered safe, also in the outpatient context, and is associated with low stress for the patient [105], [194], [195].

Possibilities for suction, treatment of circulation disorders, the availability of an emergency kit, and the possibility of timely emergency bronchoscopy are recommended for safe performance of flexible endoscopic swallowing examinations [196]. 

###### Flexible endoscopic evaluation of swallowing with sensory testing (FEESST)

A specially-developed rhino-pharyngo-laryngoscope (Pentax Precision Instruments Company, Orangeburg, New York) is equipped with a channel through which a defined air flow is released into the larynx (50 ms with a pressure of 0–15 mmHg). The data of the patients are evaluated, i. e. if and at which pressure they notice the stimulus [178] and/or triggering of the laryngeal adduction reflex (LAR, short glottic closure as stimulus response) [197], [198], [199], [200]. The examination is also called laryngopharyngeal sensory discrimination threshold testing (LPSDT) and is useful for definition of the threshold. 

###### Pharyngo-laryngoscopy with bronchoscopic examination

At the beginning or during bronchoscopic examination, a transnasally inserted flexible bronchoscope can also be used to perform an examination of the velum, the pharynx, and the larynx as well as a swallowing evaluation. This is especially recommended in patients in intensive care units and patients with reduced consciousness or ability to cooperate. For this purpose, liquids (water, saline solution, local anesthetics) are introduced into the pharynx via the suction channel of the endoscope and the triggering and process of the swallowing reflex can be immediately observed [179], [180]. Before a local anesthetic is applied for bronchoscopy, a swallowing examination with food and liquid can be performed in patients who are awake (functional endoscopic swallowing examination). After passage of the glottis (which is usually anesthetized after the swallowing examination), evaluation of the trachea and the bronchial system is possible, as is the assessment of aspirated substances that have reached the deep airways.

### 4.5 Examination modalities and evaluation criteria of video-endoscopic analysis

Despite numerous published examination protocols, there is no gold standard so far [37], [157], [161], [175], [201], [202]. The most important assessment parameters are listed in Table 13 (Tab. 13).

### 4.6 Functional tests

Before performing tests with food and liquid, an evaluation of movements of the pharynx and larynx is required. Laryngeal closure can be impaired by:

- Defects due to surgery
- Fixation of the crico-arytenoid joint or other mechanical impairments, e.g. after intubation or in cases of sclerodermia, rheumatoid arthritis
- Pareses of the vocal folds or hyperkinesia (see above)
- Reduced intensity of the medial laryngeal compression, e.g. in Parkinson’s disease, encephalomyelitis disseminate, atrophy of the intrinsic laryngeal muscles at higher ages
- Incomplete or delayed triggering of the swallowing reflex and/or incomplete or missing laryngeal elevation [115]
- Reduced or missing sensitivity of the larynx [203]

#### 4.6.1 Evaluation of the closure of the glottis and supraglottis

- Phonation of [e:]: sustained for several seconds in a middle register as relaxed as possible: assessment of tension and motility of the vocal folds as well as glottic closure
- Repeated phonation of [e] at short intervals: evaluation of the diadochokinetic motility (which is reduced in spastic paresis)
- Loud phonation of [iii:] as high as possible: evaluation of the extension of the vocal folds and elevation of the larynx. At the same time, the pharyngeal contraction is observed that is visible in the lateral contraction of the pharyngeal muscles and the narrowing of the piriform recess (“pharyngeal squeeze”) [204], [205], [206].
- Slightly holding one’s breath: glottic closure
- Holding one’s breath, slight and more intensive pressing: the arytenoids are pressed together and moved in anterior direction; closure of the false vocal folds; contraction of the aryepiglottic folds; dorsal inclination of the epiglottis [27], [157], [207], [208]. The last-mentioned finding is particularly important in patients without complete glottic closure!
- Voluntary coughing: glottic closure or closure of the false vocal folds with adequate blow
- In case of residues/penetration/aspiration of saliva or secretion: evaluation of the ability to clear the pharynx and larynx by means of changed posture, harrumphing, coughing, and repeated swallowing or, if necessary, spitting out.

#### 4.6.2 Sensitivity tests

Sensitivity can be evaluated with a flexible endoscope by applying liquid into the pharynx, a defined air flow (see above), or touching the epiglottis, false vocal folds, arytenoids, and vocal folds with the tip of the endoscope via the suction channel. During examination with a rigid laryngoscope, the laryngeal entrance may be touched with a curved cotton carrier in cases of urgent indication.

A pharyngo-laryngeal sensitivity disorder is associated with a high risk of aspiration [115], [200], [209]. In cases of extensive passage of saliva into the subglottic space without coughing, any oral food intake must be omitted prior to further diagnostics. An already inserted tracheal cannula must be blocked! In patients suffering from these symptoms, tracheostomy can sometimes be avoided or decannulation can be accelerated by drug-induced reduction of saliva.

#### 4.6.3 Verification of swallowing with food

The evaluation of swallowing with food is performed when no saliva aspiration is found and effective coughing is possible so that substances that have possibly entered into the trachea can be cleared. The patient’s safety is paramount. For the examination, the following material should be available [37]:

- Cup/glass, spoon, straw
- Ice (frozen tea or juice, as cube or crushed)
- Pulpy substances: for example jelly, vegetable mash, yoghurt
- Thickening agents for variation of the liquids
- Soft food: potatoes, vegetables, white bread
- Solid food: brown bread, rolls, biscuits, cake
- Food coloring in order to mark the substances for differentiation from mucosa. Sometimes special coloring of the different consistencies may be helpful [210].

Patients who have not received oral food intake before are first given small quantities (about 1/3 of a tea spoon) of frozen jelly. For most patients, the semi-solid consistency is easier to swallow than liquid or solid food, and the cold stimulus possibly provokes better triggering of the swallowing reflex and a more intensive swallowing procedure. Jelly passes easily and becomes fluid after warming in the body. So it can be coughed or suctioned out more easily, or is absorbed without important symptoms of irritation. Green or blue coloring can be well differentiated in the pharynx, allowing the bolus to be pursued (transnasal examination). Immediately after swallowing, residues/penetration/aspiration can be identified (by transnasal or transoral examination).

If the mucosal surface is very dry (e.g. after radiotherapy), thickened liquid (e.g. carrot juice or coffee) or finely mashed vegetables or applesauce are more appropriate.

If oral nutrition is possible, the endoscopic examination is performed first with the consistency that is most comfortable for the patient to swallow (usually pulpy substances). If effective coughing is present, yoghurt may be used. Yoghurt's slightly acidic property enhances sensory input, so it passes easily and is well visible. Depending on the endoscopic findings obtained so far, the intake of solid food (mostly buttered bread) or liquids (coffee, tea) is evaluated.

The most important events during the pharyngeal phase, i.e. laryngeal closure and opening of the upper esophageal sphincter, can only be assessed by transnasal endoscopic examination when the tip of the endoscope is in position 3 in anterograde or 4 in retrograde perspective and allows sufficient space for movement. Assessment of intradeglutitive aspiration is also generally not possible and can only be assumed from staining of the internal laryngeal mucosa and the subglottis as a sign of incomplete laryngeal mucosa. Intradeglutitive aspiration can only be confirmed by radiological examination or retrograde laryngoscopy through a tracheostoma.

If, after swallowing, no significant aspiration is observed, a higher quantity is used (1 tea spoon = about 3 ml), followed by a small table spoon (= 5 ml). Then the next consistency is tested.

A corresponding therapeutic concept can be developed from the symptoms observed and their possible pathophysiological correlation (Table 14 (Tab. 14)).

#### 4.6.4 Verification of the effectiveness of therapeutic maneuvers

The effects of the following actions can be immediately checked:

- Changed posture
- Clearing techniques
- Swallowing techniques

#### 4.6.5 Severity of aspiration and/or effective transportation of substances

The objectives of diagnostics of patients with dysphagia (including clarification of the pathophysiological origins of the disorders) are:

1. Decision-making regarding the immediate securing of the airways: in severe cases tracheostomy and the application of blocked cannulas
2. Decision-making regarding the mode of nutrition: oral food and liquid intake is possible with/without impairment, nasogastric feeding tube, PEG/PEJ.

In the course of the disease, it is necessary to evaluate whether a functional swallowing therapy is leading to improvement or even whether surgical intervention must be discussed [37]. For such a far-reaching measure, intensive evaluations of the clinical, endoscopic, and/or radiological signs of aspiration as well as of the severity are required. Different classifications have been elaborated that evaluate different examination modalities (clinical assessment, video-endoscopy, video-fluoroscopy) and different criteria (suspect findings of clinical examination including dysarthritic symptoms, residues, symptoms of aspiration of saliva and/or food, ability to actively intake food). Only some of them are evaluated and validated.

#### 4.6.6 Penetration aspiration scale (PAS)

This eight-step classification was presented by Rosenbek et al. in 1996 [107] (Table 15 (Tab. 15)). It was well evaluated for radiological findings and for endoscopic examinations [169], [211]. It proved to be appropriate for clinical use and for the evaluation of studies. The German version was validated by Hey et al. in 2014 [212].

PAS has a high significance and good diagnostic accuracy [169]. It evaluates mainly the presence of and reaction to substances in the larynx/trachea. However, the extent of residues, i.e. the effectivity of bolus transportation, is not considered.

#### 4.6.7 Flexible endoscopic evaluation of dysphagia (FEED)

There is no regulation for the examination procedure. However, an evaluation scheme was developed from the practice of examinations at the Charité, University Hospital of Berlin, Germany, and the University Hospital of Zurich, Switzerland. It is based on flexible endoscopic swallowing examination and provides additional information on the patient’s situation. 

The basic examination includes the following aspects:

- Reason for examination
- Type of nutrition, oral, parenteral, via nasogastric feeding tube or PEG
- Presence of tracheal cannula at the time of examination
- Therapy up to now
- Condition at the time of examination
- Body control
- General condition
- State of consciousness
- Cooperativeness
- Voice, speech, speaking

Examination findings:

- Function of the facial nerves
- Quality of the oral mucosa
- Motility and strength of the tongue
- Drooling

For examination of the swallowing act without food, the following aspects are described:

- Quality of the mucosa in the oro- and hypopharynx
- Velo-pharyngeal closure
- Motility of the vocal folds
- Closure of the larynx when holding the breath and pressing: vocal folds, supraglottic structures
- Sensibility of the hypopharynx and larynx
- Residues, penetration, aspiration

During endoscopic swallowing examination, different consistencies are evaluated: e.g. jelly, pudding, water, cake. For each consistency, an evaluation is performed on:

- Control of the bolus
- Triggering of the swallowing reflex
- Leaking
- Elevation of the larynx
- Classification according to the penetration-aspiration scale (PAS)
- Effect of maneuvers on swallowing (for example hard swallowing, inclination of the head, Mendelsohn‘s exercise, supraglottic swallowing, supersupraglottic swallowing, turning or tilting of the head)

The results of the examination lead to therapeutic consequences:

- Functional therapy of dysphagia
- Facio-oral tract therapy
- Feeding tube (nasogastric tube, PEG)
- Recommended diet
- If needed: surgical therapy

#### 4.6.8 FEES

FEES is a method of examination and evaluation [2], [175] and is generally used as a synonym for flexible endoscopic examination of swallowing with defined examination steps.

With the inclusion of video documentation and observation of the extensive FEES protocol, reliability has been increased. For evidence-based examinations, uniformity of the examination procedure and evaluation is required [213]. By means of a documentation software, Hey et al. succeeded in reducing the time of evaluation from 42 to 18 minutes [214]. In practice, however the examination steps are not followed consistently. This is due, on the one hand, to the duration of the examination. On the other hand, the complete protocol cannot be performed in all cases due to patients’ unwillingness to undergo the complete examination or because the symptoms and signs of dysphagia are not uniformly related to the basic disease.

#### 4.6.9 Bogenhausener Dysphagia Score (BODS)

The Bogenhausener Dysphagia Score (BODS) includes 2 different scales and assesses the ability to swallow saliva for protection of the deep airways (BODS-1) and the ability to take in food (BODS-2). The scales can be used separately or for overall evaluation to determine the severity of a dysphagia. They are useful for patients with and without tracheal cannulas. The reliability and content validity of BODS are proven [215]. See tables and recommendations for the use of BODS (Table 16 (Tab. 16), Table 17 (Tab. 17), Table 18 (Tab. 18), Table 19 (Tab. 19)).

The Bogenhausener Dysphagia Score, BODS-2, evaluates the possibilities of food intake. It replaces the “food oral intake score” as a more detailed score (FOIS) [216].

#### 4.6.10 Classification of the severity of aspiration

Based on the video-laryngoscopic findings, a classification of the aspiration symptoms according to severity was developed in 1996 which includes the degrees 0-IV (see Table 20 (Tab. 20)) [217].

In cases of severity I and II, conservative measures (diet, functional swallowing therapy, reflux prophylaxis) may suffice. In the context of severity degree III, more far-reaching decisions must be discussed, like, for example, forbidding oral food intake or even tracheostomy and blocked cannula. With this classification, the presence of a coughing reflex and the ability to expectorate voluntarily are also assessed.

Since endoscopy cannot define the whole swallowing process but only part of the origins of the disorders, video-fluoroscopy should be performed additionally, especially for initial diagnostics of a clinically relevant dysphagia.

After the introduction of high-speed cinematography for analysis of the swallowing act [218], radiological examinations were first considered as the gold standard for many years [156]. Optimal assessment of the complete swallowing process, however, is associated with high technical expenditure, with radiation exposure, and can only be performed with cooperative patients. Based on the radiological findings, the severity of the aspiration is classified according to Hannig et al. [218] (see Table 21 (Tab. 21)).

The pooling score presented by Farneti in 2008 [219] evaluated residues as well as collections in the laryngeal entrance and the glottis (i.e. actually penetration and aspiration) in relation to location, quantity, and “management” (controllability by the patient). However, it is not sufficiently validated.

The secretion evaluation scale described by Murray et al. [25] has also been validated in its German translation and is available as a long and a short version [220].

Numerous other evaluation protocols can be used for the requirements of the different patient groups under clinical or scientific aspects [101], [201], [202], [216], [219].

### 4.7 Transnasal video-panendoscopy (ViP)

Transnasal video-panendoscopy was developed in Europe and published in the USA in 1997 [182] (see video at https://www.thieme-connect.de/products/ejournals/html/10.1055/s-0035-1545298#N71110). It can be applied to examine the larynx, pharynx, esophagus, and stomach in addition to the nose, the paranasal sinuses, and tubes by applying ultrathin flexible endoscopes.

Measurements of the nasal cavity showed that flexible endoscopes with an external diameter of 4.0 mm can be introduced through the nose in 94% of adults, at least on one side, without causing trauma or pains. Usually, only a slight pressure sensation is noticed. The length of the endoscope should amount to 60–100 cm and it should be possible to turn the tip of it by up to 200° for a retroflexed view [221].

**Figure 19:**
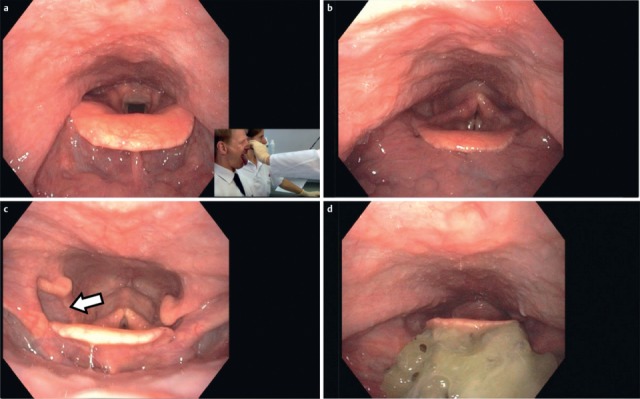
Position 1 – view over larynx and pharynx, optic window (oF) = level of the palatal arch: a – with the tongue stuck out, the valleculae can be directly seen; b – pharynx and larynx in phonation of “I”; c – the Valsalva maneuver shows the structures of the thyroid cartilage and pouch (arrow: muscle-free triangle of the thyrohyoid membrane); d – valleculae and base of tongue prepare the kiwi bolus.

**Figure 20:**
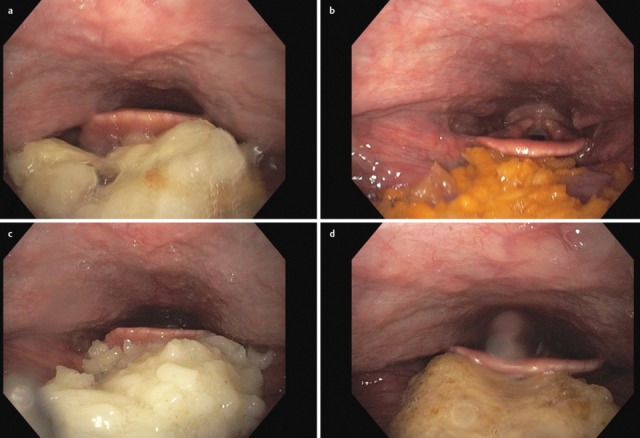
Position 1 – oF = level of the palatal arch, the bolus is collected over the back of the tongue in the valleculae: a – preparation of swallowing apple; b – preparation of swallowing carrots; c – preparation of swallowing spaghetti; d – preparation of a semi-solid bolus.

**Figure 21:**
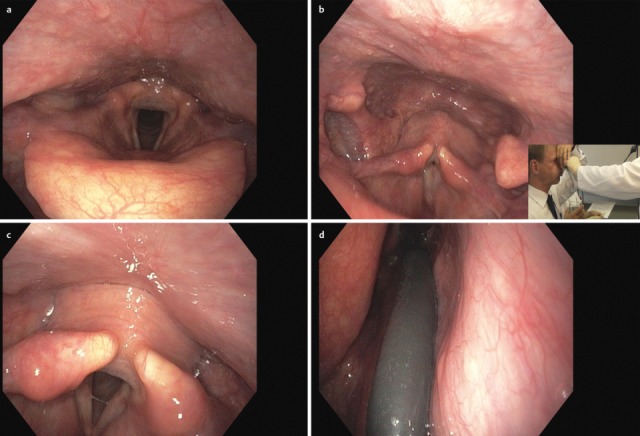
Position 2 – a – oF = level of the upper epiglottis edge, view over larynx and dorsal pharynx; b – position 2 – oF = Valsalva maneuver; c – position 3 – oF = anterograde view above the interarytenoid region; d – position 3 – oF = retrograde view above the interarytenoid region.

**Figure 22:**
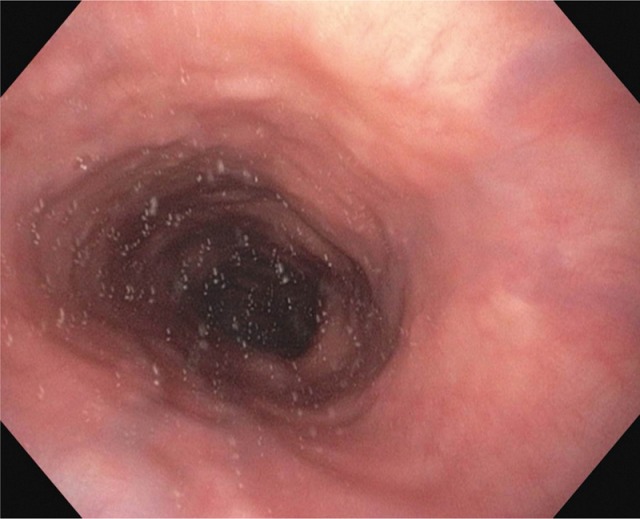
Middle esophagus: particles are visible under gas pressure and “fly” through the open esophageal lumen due to a pressure gradient (gas reflux)

**Figure 23:**
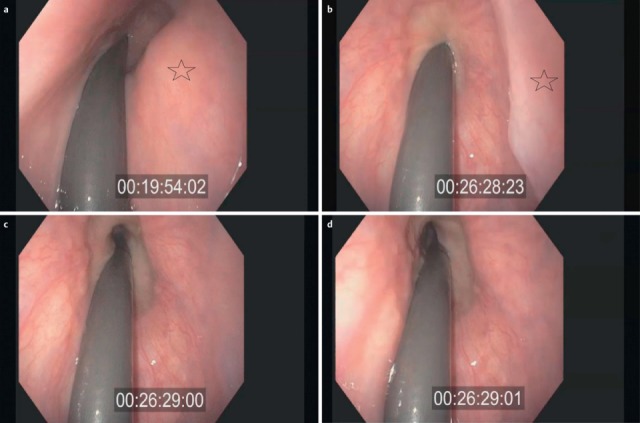
Position 4 – upper esophageal sphincter during eructation in a retrograde view (on the right side, a vertebra is visible (*)); a – upper esophageal sphincter at rest without increased pressure; b – the lower sphincter was open (pressure balance); the space of the upper esophageal sphincter is wider because of increased pressure, the circular muscles are tense (yellow color); c – the circular fibers open first (NB time code); d – the cricopharyngeal muscle is open, its contours are visible (NB time code).

**Figure 24:**
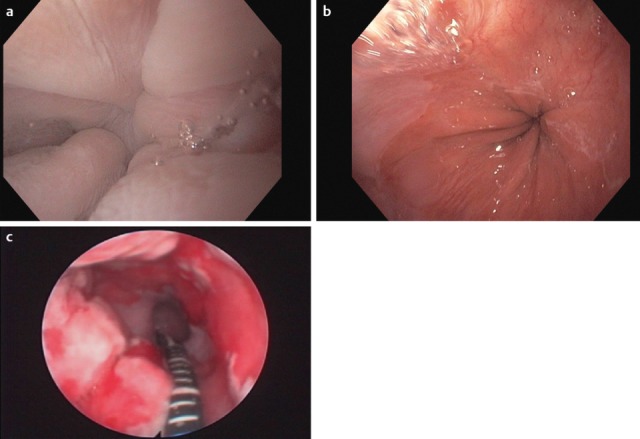
Position 5 – in front of the lower esophageal sphincter; a – clear water is collected in front of the cardia; b – the cardia opens; the esophago-gastric transition becomes visible; c – Barrett’s mucosa in the distal esophagus.

For flexible transnasal endoscopy, the standard positions of P0-P6 in anterograde and 180–200° in retroflexed view are defined in order to allow comparison [182], [222]. These positions and the corresponding optic window (oF) in anterograde and retroflexed view are (Figure 19 (Fig. 19), Figure 20 (Fig. 20), Figure 21 (Fig. 21), Figure 22 (Fig. 22), Figure 23 (Fig. 23), Figure 24 (Fig. 24)):

- Position 0: the optic window is positioned at the border of the hard and soft palate. Anterograde view: nasopharynx, tube entrances, and velum. Retroflexed view: septum, turbinates, nasal floor and roof of the posterior to middle nose on the contralateral side.
- Position 1: the optic window is at the level of the palatal arches. Anterograde view: pharynx and larynx with epiglottis. Retroflexed view: vomer, septum, and turbinates of the posterior nose on both sides.
- Position 2: the optic window is positioned behind the upper epiglottic edge. Anterograde view: petiolus and larynx as well as hypopharyngeal entrance on both sides with posterior wall of the pharynx. Retroflexed view: nasopharynx, view through upper esophageal sphincter into the oral cavity.
- Position 3: the optic window is located above the postcricoid region at the posterior pharyngeal wall. Anterograde view: arytenoids and interarytenoid region, hypopharyngeal entrance on both sides. Retroflexed view: laryngeal epiglottis, velum, base of tongue, upper esophageal sphincter.
- Position 4: the optic window is at the level of the upper esophageal sphincter. Anterograde view: upper-middle esophagus with food transportation and subsequent peristaltic wave. Retroflexed view: upper esophagus with upper esophageal sphincter including UES-function.
- Position 5: the optic window is positioned over the ampulla or 5 cm above the lower esophageal sphincter. Anterograde view: the whole circumference of the lower esophageal sphincter. Retroflexed view: lower and middle esophagus.
- Position 6: the optic window is positioned below the lower esophageal sphincter. Anterograde view: view on the stomach content or the corpus region. Retroflexed view: while the retroflexed endoscope passes the cardia, one can test the resistance of the lower esophageal sphincter. In the stomach the transport time of the bolus from the swallowing movement of the larynx to the arrival of the bolus in the stomach can be measured. After rotation, view on the stomach fundus.

Functional endoscopic swallowing examination relates to the pharynx and thus only uses the positions 0–3. During this examination, the patient has a foreign body sensation in the nose. Decongesting nose drops with local anesthetics may minimize this sensation. Cotton soaked with oxybuprocain is inserted in the nose and, after about 5 minutes, causes superficial anesthesia. Touching the lateral walls of the nasopharyngeal space, the epiglottis, or the interarytenoid region during endoscopy should not be performed outside the swallowing act, if possible, because the gag reflex might be triggered. The intake of food and beverages in the mouth is associated with physiological hypoesthesia, which allows passage into the esophagus. The patients can follow the procedure of endoscopy on a screen. In this way, the examiner has the possibility of demonstrating the endoscopic results and immediately answering any questions arising during the examination. In this way, trust between the physician and his patient is increased. 

#### Nose

The procedure of passing the nasal cavity corresponds to functional endoscopic swallowing examination (see 4.4.2.2). 

#### Nasopharynx

When the tip of the endoscope reaches the dorsal end of the vomer, the endoscope is positioned near the posterior pharyngeal wall exactly in the midline between the tori tubarii (position 0 – border between hard and soft palate). The patient should breathe calmly through his nose. In this way, the velum relaxes and opens the view over the oropharynx to the larynx and hypopharynx. Incautious touching of the lateral nasopharyngeal wall or of the ostia may lead to swallowing and gagging. Patients with rhonchopathy, especially at higher ages, and adipositas have a thickened and prolonged velum. At the same time, sensibility in this area is reduced. 

#### Oropharynx

When the tip of the endoscope is brought close to the dorsal pharyngeal wall, it is inclined in the direction of the oropharynx with the control lever and advanced until the larynx becomes visible. The pharynx and the larynx are evaluated in plan view from the palatal arches (position 1: level of the palatal arches, Figure 19 (Fig. 19) and Figure 20 (Fig. 20)) with regard to breathing, coughing, eating, drinking, and speaking.

The passage from the palate to the larynx is wide and allows a good assessment of the anatomical structures. In this context, it is important to know that intake of food and liquid leads to physiological hypoesthesia. This starts, for example, when the lips come into contact with a straw for drinking. This process continues until the lips release the straw and finally the pharynx relaxes. Touching the epiglottis with the endoscope during this process does not lead to gagging or vomiting (position 2: level of the upper epiglottic edge, Figure 21 (Fig. 21)). 

#### Hypopharynx and upper esophageal sphincter

Before the first passage of the upper esophageal sphincter, it is necessary to test whether the patient is able to drink continuously. The patient should be able to drink at least two swallows of clear water without breathing. In order to pass the upper esophageal sphincter, the tip of the endoscope should be placed near the posterior hypopharyngeal wall at the level of the epiglottis (position 3: near the postcricoid region). The physician holding the endoscope encourages the patient to drink continuously. During the swallowing act, the endoscope is inserted through the upper esophageal sphincter and its function is documented. Afterwards, the pathway into the esophagus is free and the endoscope is continuously advanced. If the patient makes a sudden unexpected movement before swallowing, touching the epiglottis with the endoscope may lead to triggering the gag reflex. This makes further endoscopic evaluation difficult or even impossible. When removing the endoscope from the esophagus, the patient should be invited to drink before the upper esophageal sphincter is passed again, as this helps to avoid the gag reflex when drawing back through the upper esophageal sphincter. Alternatively, the endoscope can be removed very slowly and without irritation through the upper esophageal sphincter. 

#### Larynx

When the tip of the endoscope reaches the pharynx, the patient is asked to stop drinking. Now the tip is advanced with the lever. This view allows evaluation of the endolarynx from a nearby position and the vocal folds in particular can be examined during breathing and phonation as well as the trachea (position 3), also with stroboscopy. Penetration and aspiration can be excellently revealed. 

#### Esophagus

The endoscope is advanced into the esophagus during continuous swallowing of clear water so that the patient does not notice the movements of the endoscope (Figure 22 (Fig. 22)). When the gastro-esophageal transition zone becomes visible during swallowing of clear water, the ability to close the lower esophageal sphincter can be tested and evaluated by means of the sniffle test. 

Sniffling means a short and rapid nasal inspiration associated with parallel contraction of the diaphragm, the upper esophageal sphincter, and the rhino-sphincter. When the distal esophagus is open, the level of the diaphragm can be determined by its contraction in the sitting patient.

Afterwards, inversion of the endoscope in the lower esophagus ampulla can be performed at about 5 cm above the lower esophageal sphincter. The radius of the endoscope during 200° flexion amounts to 2.5–3.5 cm. By using thinner endoscopes with a smaller radius, irritation of the esophagus is reduced. After inversion, the endoscope is removed in retroflexed view to the upper esophageal sphincter (position 4: upper esophageal sphincter, Figure 23 (Fig. 23)).

If the esophagus is collapsed, for example in the context of hiatus hernia, the lumen can be kept open and assessed during continuous water drinking. A distinction must be made between hypertonicity, hypotonicity and normal muscular tonus of the upper esophageal sphincter. In cases of hypertonicity, a prolapse of the postcricoid cavernous mucosa occurs. In cases of hypotonicity, the circular muscles that are caudal at the cricopharyngeal muscle do not close. Their ability to close can be tested by means of the sniffle test. If both parts of the sphincter are impaired in terms of closure, the view to the posterior caudal wall of the larynx is free. The larynx sticks like a plug at the open esophageal entrance. Patients complain about permanent eructation.

In the next step, the function of the esophagus is controlled in retroflexed view during swallowing of solid and liquid food. Here, the endoscope is again slowly advanced in a distal direction to the lower esophageal sphincter (position 5, Figure 24 (Fig. 24)). At the same time, bolus transportation and the peristaltic wave of the esophagus can be observed in a retroflexed and anterograde view. 

#### The lower esophageal sphincter

For passage of the endoscope through the lower esophageal sphincter in inverse view (retroflexed view), the examining person asks the patient to stop drinking. When the inverted endoscope passes the sphincter, the examiner gets an impression of the tonus and the closure of the lower esophageal sphincter. In addition to anamnesis and local findings, the resistance of the lower esophageal sphincter is crucial for the assessment, and for inducing further diagnostics like, for example, high-resolution manometry. 

#### Stomach

The function of the lower sphincter (esophagus and transition zone to the stomach) is observed in retroflexed view in sitting patients, even with a full stomach (position 5).

Often the sliding movement of a hiatus hernia becomes visible. While liquid or solid food are taken in, bolus transportation from the esophagus to the stomach can be examined together with the function of the lower esophageal sphincter. 

#### The way back

After examining the stomach and the lower esophageal sphincter, the inversion can be changed to the anterograde position in the ampulla and the endoscope can be drawn back slowly. When removing the tip of the flexible endoscope from the esophagus, the patient should drink clear water. The laryngeal mucosa of the epiglottis should not be touched outside of swallowing while the instrument is being removed. 

#### Pharynx, oral cavity, and nose in retroflexed view

With the aid of 2 or 3 mouthfuls of water, an experienced examiner can pass the upper esophageal sphincter with the inverted endoscope. The postcricoid region is reached without any problems and without triggering the gag reflex. The tip of the endoscope is positioned at the transition of the oropharynx to the hypopharynx (position 3) and in retroflexed view, if necessary, can be moved upward and downward. During this maneuver, the patient has to drink sufficiently in order to support the natural hypoesthesia and suppress the gag reflex in the pharynx.

From the postcricoid region, the function of the palatal arches, the base of tongue, and the opening or closure of the nasopharyngeal space can be observed. Bolus accumulations and subsequently also the passage of the bolus as triggering mechanisms for the swallowing act can be seen. Furthermore, hyperplastic tonsils as well as the function of the tongue in oral and oropharyngeal dysphagia in interplay with the soft palate and the palatal arches become visible.

### 4.8 Further diagnostic procedures

Despite improved endoscopic techniques, motor function of the mouth, triggering of reflexes, opening of the esophageal sphincter, and esophageal motility cannot be satisfactorily observed. In some structural changes (e.g. diverticulum, disorders of the cervical spine), associated functional impairments cannot be evaluated, nor can the quantity of aspirated substances. In such suspicious cases, radiological examination with presentation of the complete swallowing act is essential.

Procedures with a rapid-image sequence are necessary in order to assess even short discrete aspiration episodes:

- High-speed cinematography: 50–200 images per second; relatively high radiation exposure
- Video-fluoroscopy (“Video-fluoroscopic Swallowing Study”, VFSS): 25–30 images per second; still a common technique; further development as digital fluoroscopy (“Digital Fluoroscopic Swallowing Study”, DFSS) with newer image assessment
- Pulsed radioscopy: dose-reduced; high image quality; compatibility with PACS systems (picture archiving and communication system); adaptation of the imaging rate possible, e.g. when passing the esophagus

The evaluation is performed, in a way comparable to endoscopic swallowing examinations, with different consistencies and quantities (contrast enhanced) and includes the effectiveness of clearing and swallowing techniques.

pH measurement is essential when reflux disorders are suspected. While aspiration of gastric juices may lead to life-threatening pulmonary complications, acid stomach contents in the esophagus may also lead to an increased disturbance of the opening of the upper esophageal sphincter.

Manometry of the pharynx and esophagus clarifies the deficits of food transportation (impaired propulsion) and disorders of the sphincter function as observed in patients with myositis. The significance can be increased in combination with radiological examinations (“radio-manometry”).

Bronchoscopy is performed as a diagnostic tool when pulmonary complications caused by aspiration are suspected in order to identify signs of inflammation, aspirated substances in the trachea and the bronchial tree, and also to gain material for bacteriological testing. Therapeutically, aspirated foreign body particles, saliva, secretion, pus, and food are removed by bronchial lavage. 

For further questions, EMG, ultrasound, MRI, and scintigraphy may be indicated.

## 5 Therapeutic consequences

### 5.1 Conservative therapy of dysphagia

Targeted therapy should start as early as possible. In the acute phase, the aim is to protect against aspiration. Generally, adaptive and compensatory measures are applied initially, followed by restituting procedures. Therapeutic frequency depends on the time of the event that has triggered the swallowing disorder. In the acute phase, swallowing therapy should be performed that takes the basic disease of the patient into consideration and includes longer or shorter therapy sequences of one or several times per day (at least five times per week) according to the patient’s capacity.

In the post-acute phase, a single therapy of 45 minutes at least once per day is recommended along with self-exercises. In subsequent outpatient therapy, 1–2 times per week the exercises should be performed for 45 minutes in order to continue with the progress of the therapy, maintain the competence achieved, and allow transfer into everyday life. The therapy ends when the therapeutic objective or a certain level is achieved, or when motivation for therapy is missing.

Generally, 3 approaches are distinguished: functional dysphagia therapy (FDT), facio-oral tract therapy (F.O.T.T.^®)^, and proprioceptive neuromuscular facilitation (PNF).

#### 5.1.1 Functional therapy of dysphagia

The term functional dysphagia therapy was coined by Gudrun Bartholome [47]. It describes a function- and problem-oriented procedure which is based on restitutive (causal), compensatory, and adaptive methods.

##### 5.1.1.1 Restitutive (causal) methods

With these methods, an attempt is made to restitute the swallowing function as far as possible by stimulation along with activating and repetitive exercises without food intake. The different elements are: 

###### Thermal stimulation

Thermal stimulation is based on the assumption that the anterior palatal arches are sensitive areas that react to temperatures. The therapy is expected to facilitate triggering of the swallowing reflex. It is indicated for reduced oral and pharyngeal sensitivity.

A thermal probe is cooled down with ice. The palatal arches are touched or the patient crushes particles of ice cubes to let them flow around the base of the anterior palatal arches. Then the patient is encouraged to swallow.

The intended effect consists of rapid triggering of the swallowing reflex and improvement of pharyngeal sensitivity. The effectiveness has been empirically evaluated and confirmed, although it remains the subject of controversial discussion [107], [156]. 

###### Masako maneuver

The indication for the performance of this maneuver [223] is a weak base of the tongue and too low pressure at the beginning of the pharyngeal phase. Patients are instructed as follows. The tongue has to be held between the teeth and the patient is asked to swallow. The effect consists of a protrusion of the posterior pharyngeal wall that approaches to the base of tongue and improves the build-up of pressure. 

###### Shaker maneuver

In cases of dysfunction of the upper esophageal sphincter, the following maneuver may be indicated [224]: The patient has to lie on his back, and his head should be lifted 3× for 1 minute (the shoulders remain relaxed, a pause of one minute between the single exercises), then his head is lifted briefly 30 times. This exercise should be repeated 3 times per day, 5 days per week and for 6 consecutive weeks. The effect consists of a wider and longer opening of the upper esophageal sphincter, an increase in bolus pressure, and improvement of the elevation of the larynx.

##### 5.1.1.2 Compensatory methods

Swallowing exercises and posture changes with food should lead to different techniques of swallowing to compensate deficits in the best way possible even if the physiological movements cannot or can only partly be restituted. 

In the case of hyperkinesia, the patient has to learn compensatory strategies to achieve laryngeal closure. This can be achieved, for example, by dividing the liquid in the oral cavity into very small gulps, changing the posture of his head, intensively chewing solid food, collecting smaller boluses in the valleculae, and waiting for the beginning of breathing because of the prolonged bolus peristalsis time (BPT). 

###### Mendelsohn’s maneuver

The effect of this maneuver is restitutive and compensatory [225]. It is indicated for dysfunction of the upper esophageal sphincter, reduced laryngeal elevation, and reduced propulsion of the tongue.

The patient is instructed to lift his larynx during swallowing for 2–3 seconds. The back of the tongue should be pressed against the palate. Then he should relax and repeat swallowing.

By prolonging the swallowing act, the upper esophageal sphincter becomes longer, which improves pharyngeal contraction. Retentions are reduced and laryngeal elevation is prolonged. 

###### Supraglottic swallowing

If reflex triggering is delayed and laryngeal closure is incomplete, supraglottic swallowing is trained [156], [226]. The patient is instructed to hold his breath, swallow, and harrumph/cough immediately afterwards without taking breath. Then swallowing is repeated. Supraglottic swallowing mobilizes and removes retentions and penetrations, which are swallowed afterwards. 

###### Super-supraglottic swallowing

This maneuver is performed as described for supraglottic swallowing. In addition to holding his breath, the patient is asked to press. In this way, closure of the glottis and the larynx is stimulated. The deep airways are better protected. 

###### Posture changes

Changes of posture modify gravity conditions by inclining the head and the bolus pathway by turning the head in order to regulate bolus transportation. Because of individual anatomical situations, the effectiveness should be evaluated endoscopically/radiologically.

- Anterior inclination of the head: chin tuck
  In cases of reduced oral bolus control with leaking, delayed swallowing reflex, or reduced retraction of the base of tongue, the inclination of the head should support oral bolus control and protect the larynx by increased epiglottic inclination [227].
- Inclination of the head to the stronger side
  In cases of unilateral and pharyngeal paresis, the bolus should be guided safely over the stronger side.
- Turning of the head to the affected side
  In cases of unilateral pharyngeal and laryngeal paresis, the paretic vocal fold is expected to shorten and thus improving glottic closure [228].
- Turning of the head to the stronger side
  In cases of paresis of the vocal folds, the paretic vocal fold is extended in order to improve glottic closure.

Both changes of posture have the same effect of improving glottic closure. Endoscopic/radiological control is important to evaluate the effectiveness.

##### 5.1.1.3 Adaptive procedures

Adaptive procedures include, among others, dietetic measures such as thickening of food or certain food consistencies and provision of supportive devices such as cleavage goblets or special cutlery.

To facilitate food intake and reduce the risk of aspiration, the following measures are useful:

- Dietetic changes with adaptation of viscosity, temperature, taste with consideration of pulmonary toxicity
- Appropriate positioning of the bolus on the posterior tongue and the healthy side of the tongue
- Special dishes and cutlery

Furthermore, attention must be paid to:

- Optimal posture
- In the case of feeding: introducing the spoon from the anterior
- Enough time
- If needed, manual support for closure of the mouth and jaw
- If needed, initiating swallowing by striking movements
- Small bites
- Intensive chewing
- If needed, repeated swallowing
- Taking the next portion when the mouth is empty
- Avoiding mixed consistencies
- Reducing residues by compensation
- Avoiding speaking during food intake
- If needed, pausing
- If penetration is suspected, doing a phonation test in order to learn compensation

The overall effectiveness of functional dysphagia therapy is estimated as being high. More than 50% of probe-dependent patients after apoplexy achieve completely oral nutrition through swallowing therapy [102], [229]. Already in the acute phase of a stroke, starting intensive swallowing therapy is important. With “intensive” swallowing therapy in the acute phase (5× per week), significantly more patients can eat again normally after 6 months compared to patients who receive “standard” swallowing therapy (3× per week) [230].

#### 5.1.2 Facio-oral tract therapy (F.O.T.T.)^®^

F.O.T.T.^®^ is a treatment approach for diagnostics and therapy of neurogenic swallowing disorders, mimic and oral movements, swallowing, breathing, voice and speaking and was developed by Kay Coombes on the basis of the Bobath concept [231]. It is a holistic treatment concept based on interdisciplinary team work.

The general objective is the restitution of the disturbed function (restituting procedure) as well as reduction of aspiration pneumonias, contractures, hypersensitivity, and avoidance of manifest abnormal movement patterns. The central elements are whole-body tension regulation, elaboration of appropriate basic positions, and coordination of functions and movements. Besides oral hygiene, facilitation of swallowing saliva and management of tracheal cannulas are also included. The elaboration of a stable dynamic posture background and the application of tactile kinesthetic, visual, auditive, olfactory, and gustatory stimuli are basic principles. They contribute to approaching and learning the correct functions, establishing transfer capacities, and reducing or even avoiding aspiration pneumonias as well as the manifestation of abnormal movement patterns, hypersensitivities, and contractures. So far, no randomized controlled studies have been published on the clinical effectiveness. According to Frank et al., however, a comparison to conventional swallowing therapy revealed a significantly shorter duration of tracheal cannulas in patients who had be treated based on F.O.T.T.^®^ [232].

#### 5.1.3 Proprioceptive neuromuscular facilitation (PNF)

PNF was developed by Hermann Kabat and Margaret Knott. As a whole, it represents a restitutive and functional procedure. Sensomotor functions are activated by applying special stimuli (thermal stimuli, pressure, extension, resistance). The objective is toning, reinforcement, or also tonus reduction and thus improvement of movement possibilities as well as improvement of stability, performance of active movements by correct manual contact and optimal resistance, stimulation of coordinated movements with correct stimulation, and improvement of endurance or reduction of fatigue symptoms. So far, studies on the effectiveness are missing.

### 5.2 Therapy for drug-induced dysphagia

Critical discussion is necessary as to whether multiple medication is really indicated. Especially in cases of degenerative changes in the brain, attention must be paid to dose reduction of drugs in elderly patients. Often, a regular drug intake is automatically expected. However, patients must be instructed that the tablets are to be swallowed with sufficient liquid. Many tablets are difficult to swallow because of their size and surface. In such cases, other forms of administration should be provided, e.g. hard capsules. If OMIEI is suspected, esophago-gastroscopy is recommended (even in children presenting the corresponding symptoms). Depending on the findings, PPI therapy is a reasonable option. Further recommendations for daily life regarding drug intake are summarized in Table 22 (Tab. 22) [67].

### 5.3 Therapy for hypersalivation and xerostomia

#### 5.3.1 Hypersalivation

To begin with, orofacial regulation therapy or functional dysphagia therapy is indicated for control of salivation. Secondly, drug therapy is induced (see Table 23 (Tab. 23)).

Gerber et al. [233] and Noonan et al. [234] combined extirpation of the submandibular glands and a bilateral ligation of Stenon’s ducts for reduction of saliva production in order to prevent aspiration pneumonia in neurologically impaired children. This procedure must be critically discussed. A natural function is irreversibly abandoned in order to control a danger without treating the functional disorder itself. Saliva cleanses the oral cavity, controls the bacterial flora, prevents caries, keeps the mucosa smooth, supports wound healing, reduces pains, absorbs nutritional components (vitamin B12), is prophylactic against reflux, reaches the stomach before and with the food, catches it in the stomach, and covers it together with gastric acid in a sticky foam [6].

The guideline on hypersalivation provides orienting information [235].

#### 5.3.2 Xerostomia

Clinical examination (dry lips and mucosa, atrophic muscles, reddened carved tongue, no saliva on the floor of the mouth, thickened exprimat, caries, infection) and diagnostics by means of ultrasound (sialography, sialometry, sialendoscopy, MRI ^99m^TC as required; if necessary biopsy from the mucosa of the lips) indicate the symptoms of xerostomia. Clarification of the origin is not always possible when rheumatoid or drug-induced etiology have been excluded. Xerostomia can only be symptomatically treated.

- Sufficient liquid, sialologa, oral rinsing (glycerin, black tea), saliva substitutes
- Drug therapy: bromhexine, anethol (mukzinol), parasymphaticomimetic drugs: pilocarpin (salagen), cevimelin hydrochloride (evoxax), cave: side effects
- Prophylaxis for the development of post-radiogenic xerostomia: amifostin (ethyol; binding of alkylating substances and free radicals)

### 5.4 Surgical therapy of dysphagia

Regarding cases of dysphagia with chronic aspiration and even recurrent aspiration pneumonias, there is a controversy between hunger, thirst, and coughing [236]. The severity of the risk depends on the quantity of aspiration. Its assessment can be performed, even at the bedside, without sedation by applying transnasal functional endoscopy and/or long-term endoscopy, if needed up to several hours [237].

For protection of the airway, tracheostomy is the acute life-saving intervention; for securing nutrition it is the naso-gastric tube or percutaneous endoscopic gastrostomy (PEG).

The following disorders are a reason for surgical intervention:

1. Acute diseases of the motor system for reparative surgery
2. Neurodegenerative diseases as long as they are slowly progredient and there is thus a realistic chance of a lifetime with less complaints after surgery
3. Diseases associated with sensitivity disorders or loss
4. Dysphagia as a consequence of therapeutic measures for tumor disease

The most important rule for surgery of dysphagia is to preserve existing functions and residual functions of swallowing compared to the natural function. High diagnostic precision is required regarding the high number of muscles and brain nerves involved. The decision for surgical measures is made on an interdisciplinary scale in cases of neurological basic disease, and needs to be discussed intensively with the patient and adapted individually.

Organ preservation in tumor-related disorders including associated crucial risks must remain reasonable. Thus this kind of surgery becomes an individualized part of medicine in order to maintain or achieve a certain quality of life. The learning process based on documentation of long-term courses by functional examinations is an integral part of evidence-based treatment.

In the following, the surgical therapy steps with dysphagia-relevant techniques are summarized.

#### 5.4.1 Surgical therapy of specific organ structures

##### 5.4.1.1 Cleft lip and palate and velopharyngeal insufficiency and/or incompetence

Breast feeding of children with cleft lip and palate is generally possible based on specific instructions. Drinking takes more time. Only in cases of continuous cleft lip and palate should an orthodontist adapt a palatal plate within the first days of life in order to improve separation of the nasopharynx from the oral cavity.

The timing of all further surgical measures (e.g. closure of the upper lip, palatoplasty [238], creation of a sufficiently long and functional velum [239], augmentation of the posterior oropharyngeal wall with fat [238], [240]) is an interdisciplinary task with maxillofacial surgeons.

##### 5.4.1.2 Hyperplastic of the lingual tonsils and epiglottic cysts

As a consequence of reflux, the lingual tonsils may become hyperplastic. Endoscopy in a retrograde view of position 4 (below the upper esophageal sphincter) reveals that the epiglottis often immerges into the upper esophagus. Particles from the upper esophagus are thrown back from the pharyngeal epiglottis into the pharynx at the end of the swallowing act, comparable to a tennis racket, and “wiped off” from the base of tongue. If the valleculae remain hidden under the lingual tonsil when the tongue is stuck out, these findings are called hyperplasia. Only if it leads to dysphagia complaints such as globus sensation, the desire to clear one’s throat, and swallowing disorder, should the lingual tonsil be removed via laser surgery under strict protection of the muscles [241]. Modern procedures like laser surgery, TORS, and radiofrequency surgery are available for this purpose. Epiglottic cysts can be removed by marsupialization or complete excision in the context of microlaryngoscopy.

If a tumor is suspected, a biopsy is required. Lingual goiter must be excluded. A check of the function of the upper esophageal sphincter, if necessary also pH measurement in the pharynx, may be helpful in reaching a decision regarding an intervention.

##### 5.4.1.3 Eagle syndrome

**Figure 25:**
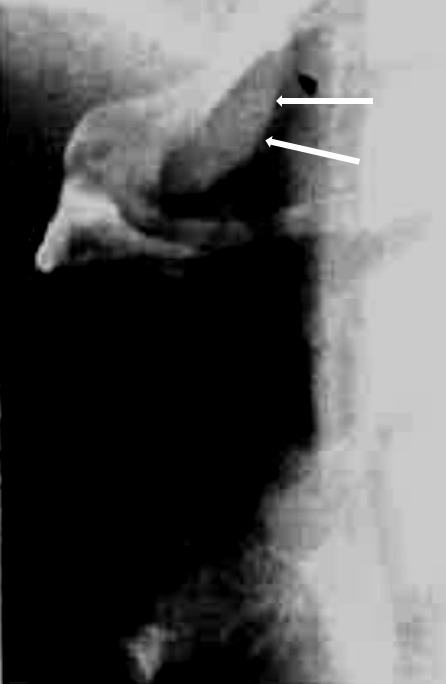
Missing laryngeal elevation during the swallowing act in Eagle syndrome. The arrows indicate the epiglottis.

Eagle syndrome is a rare disease caused by elongation of the styloid process or calcification or ossification of the stylohyoid ligament [242]. These conditions lead to an impaired elevation of the hyoid and the larynx (see Figure 25 (Fig. 25)).

The patients complain about unclear pains in the throat, swallowing disorders, foreign body or globus sensation. The symptoms may be due to pressure on the brain nerves V, IX, X as well as the common carotid artery. Surgical therapy consists of transoral or transcervical resection of the styloid or ossified ligament, if needed with resection of the lesser cornu [243].

##### 5.4.1.4 Cervicogenic dysphagia

Cervicogenic dysphagia is mostly due to functional disorders [46]. Thus, endoscopy of the aerodigestive tract as well as manual and radiological examination of the cervical spine may lead to the diagnosis of functional disorders of the cervical spine, which is then solved by mobilization or also manipulation of the cervical spine in the segment (generally C3/4) as reflex therapy procedure. This treatment can be combined with local lidocaine injection at the painful insertion of the infrahyoid muscles at the hyoid or with acupuncture. This therapy should be completed with subsequent physiotherapy. The partial or even complete removal of the symptoms by manual therapeutic treatment is clinical evidence of an existing functional disorder of the cervical spine.

In patients with organically caused cervicogenic dysphagia, modification of the food consistency may in many cases improve the complaints. Usually, Forestier’s disease remains asymptomatic. So symptomatic cervical osteophytes are a rare disease that affects mostly males. Apart from special emergency cases, conservative treatment should be preferred. The therapy is performed symptom-related while analgetics, anti-inflammatory drugs, muscle relaxants, proton pump inhibitors, and physical therapy are applied. Physiotherapy and specific swallowing therapy in particular can lead to successful improvement or prevention of dysphagia [244]. If conservative measures fail, removal of the osteophytes eliminates or palliates the symptoms when they are the reason for weight loss [50]. If the symptoms do not improve after the described therapies, application of a percutaneous endoscopic gastrostomy must be discussed after complete diagnostics.

##### 5.4.1.5 Reinforcement of the muscle-free triangle of the thyrohyoid membrane

Protrusions tend to develop in the muscle-free triangle of the thyrohyoid membrane where the superior laryngeal nerve enters the larynx with the vessels (see Figure 19c (Fig. 19) and Figure 21b (Fig. 21)). This triangle is located cranially near the hyoid bone, anteriorly and caudally at the thyrohyoid muscle and the omohyoid muscle, and caudally and posteriorly at the inferior pharyngeal constrictor muscle. Retention of parts of the bolus or of liquid in the protrusion may lead to postdeglutitive aspiration when laryngeal closure is disturbed. If not, the patient may clear the pharynx by supraglottic or supra-supraglottic swallowing.

Prior to surgical treatment of a large protrusion with aspiration, it is essential to exclude vagus paresis or paralysis. With spontaneous healing of the nerve, the protrusion probably also disappears spontaneously. Dysfunction of the vagus nerve is confirmed by video-endoscopy, video-fluoroscopy, high resolution manometry, or electromyography of the cricothyroid muscle, which is innervated by the superior laryngeal nerve. If no vagus paresis is found, surgical treatment is performed under local anesthesia [34]. The skin incision is placed at the level of the protrusion along the skin tension lines. While protecting the superior laryngeal neurovascular bundle, the thyrohyoid membrane is exposed. The protrusion becomes visible during the Valsalva maneuver. The weak wall can be reinforced by inserting a freely transplanted, adapted septum or concha cartilage of the patient and/or through transposition of the omohyoid muscle:

1. The insertion of the omohyoid muscle at the hyoid bone is transected and displaced in a lateral direction on the greater cornu to cover the protrusion. Additionally, the muscle is sutured to the thyroid cartilage, the pharyngeal constrictor muscle, and the thyrohyoid muscle [245].
2. Alternatively, the patient’s cartilage of the septum or the concha can be placed over the protrusion and fixed as described above. Both techniques may be combined.

For immobilization, a nasogastric tube is applied for one week. In order to reduce laryngeal movements, the patient is asked not to speak or swallow and, if necessary, to spit out saliva.

##### 5.4.1.6 Positioning of the laryngo-hyoid unit

An indication for surgery depends on the function of the thyrohyoid muscles, the function of the anterior suprahyoid muscles, the sensitivity of the triggering zones for the swallowing act as well as on the condition of the mucosa. Through displacement of the larynx and hyoid from the esophagus in a ventral direction to be protected by the base of tongue, aspiration may be reduced.

Different laryngeal suspension techniques have been described [246] in order to minimize the aspiration problem after tumor surgery. They used suture techniques to displace the thyroid cartilage in an anterior direction and to approach it to the mandible. Hillel and Goode suspended the larynx laterally with sutures to solve the problem of aspiration after resection of the base of tongue [247]. Herrmann described a suspension technique to the chin by using fascia lata after laryngo-hyoidopexy and myotomy of the upper esophageal sphincter in severe neurological swallowing disorders [34], [245]. This technique observes the correct position of the laryngohyoid unit in the pharynx, and considers the function of the suprahyoid muscles and the elevation of the epiglottis in order to protect the laryngeal entrance.

##### 5.4.1.7 Laryngo-hyoidopexy

At the beginning of the intervention, PEG is applied in order to postoperatively immobilize the surgical site and secure nutrition. The existing tracheostoma is circumcised and a cranially pedicled flap is prepared. If no tracheostoma is present, this is the first surgical step. The thyroid gland is identified, the isthmus is transected, and the trachea is exposed. The trachea and pharynx are disconnected from the thyroid and mobilized. The recurrent nerves are protected.

**Figure 26:**
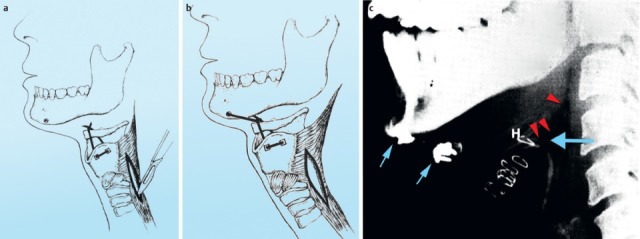
a – extension of the transcervical myotomy of the cricopharyngeal muscle (about 4 cm) with protection of the inferior laryngeal nerve with laryngo-hyoidopexy. The myotomy should be extended in a cranial and caudal direction, all muscle fibers have to be transsected in the area of the myotomy; b – additionally to myotomy, a suspension suture is placed through the mandible in order to draw the laryngohyoid complex under the base of the tongue for protection; c – 43-year-old patient after surgery of an ependymoma of the 4th ventricle. After myotomy, laryngo-hyoido-mentopexy was performed. The small blue arrows show osteosynthesis plates of the hyoido-mentopexy. The blue arrow shows the laryngeal vestibule protected by the base of the tongue. The red arrow heads show soft part opacification of the base of tongue. H = hyoid.

Afterwards the following steps are performed:

1. Myotomy of the cricopharyngeal muscle (see chapter on “myotomy of the upper esophageal sphincter”)
2. Transsection of the infrahyoid muscles. In order to remove the functionally impaired larynx from the center of the swallowing pathway and to displace it to be protected by the base of tongue, the infrahyoid muscles are transected apart from the thyrohyoid muscle [34]. The unit of larynx and hyoid can now be placed in the desired position.
3. Laryngo-hyoidopexy and suspension of the laryngo-hyoid unit to the chin. With two strong sutures (non-absorbable), the thyroid cartilage is fixed at the hyoid in such a way that the hyoid is positioned behind the anterior edge of the thyroid cartilage [248]. The sutures are performed with polytetrafluoroethylene (Gore-Tex; permanent) and Vicryl 0. In order to avoid transection of the sutures at the thyroid cartilage, the suture is performed over Gore-Tex sheets (see Figure 26a (Fig. 26)).
  After laryngo-hyoido-pexy, the threads are not shortened so that they are available for suspension. For this purpose, a submental skin incision is performed, the tissue between hyoid and chin is tunneled on the muscles, and two drill holes are placed before the exit of the mental nerve. For suspension in the direction of the chin, the use of polytetrafluoroethylene (Gore-Tex; permanent) and two Ethibond sutures is recommended. Alternatively, the tendon of the long plantar muscle located beside the Achilles tendon (about 30 cm long) or one of the long tendon of the palmar muscle (about 12 cm long), or a strong strip of fascia lata as autologous material can also be used. Via a small skin incision (2 cm) beside the ankle, the tendon is exposed and stripped. Free transplantation connects the thyroid cartilage to the hyoid. It is conducted to the chin, threaded, and sutured with a continuous tendon suture technique – adapted to the material (Figure 26 b, c (Fig. 26)) [245].
4. In order to avoid overcorrection, i.e. closure of the laryngeal entrance, the sutures for suspension are placed under endoscopic control.
5. Tracheostoma: since the laryngo-hyoid unit is displaced in a cranial and ventral direction, the tracheostoma, which is now higher than it was before, must be renewed at the end.

PEG nutrition and a blocked cannula are generally maintained for about 10 days.

The long-term results of this technique (n=17; mean follow-up of 4.4 years) are given with 53% (n=17) as a success, i.e. exclusively oral nutrition, with 18% as a partial success, i.e. long-term nutrition, orally with modified consistency, and 30% as a failure, i.e. no or only very reduced oral nutrition [249].

##### 5.4.1.8 Protection of the laryngeal entrance (lowering of the epiglottis)

The epiglottis keeps its slightly inclined position due to the thyroepiglottic ligament, the pre-epiglottic fatty tissue, the glosso-epiglottic fold, and both pharyngo-epiglottic folds.

**Figure 27:**
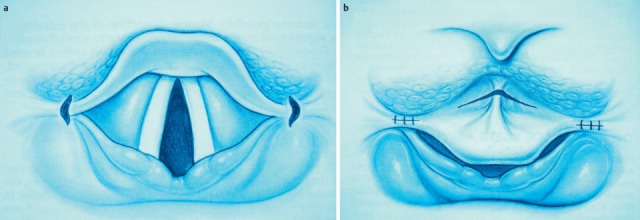
a – for epiglottic lowering, the plicae can be elongated or transected; b – after transection and suturing of the plicae pharyngoepiglotticae, an additional stepwise disconnection of the plica glossoepiglottica is possible.

Endoscopically, the epiglottis can be lowered in a dorsal direction to protect the laryngeal entrance by applying a technique described by Laurian [250] or, in a modified manner, by bilaterally transecting the plicae pharyngoepiglotticae and disconnecting the plica glossoepiglottica from the tongue (Figure 27 (Fig. 27)) [35].

##### 5.4.1.9 Insufficiency of glottic closure and dysphagia

**Figure 28:**
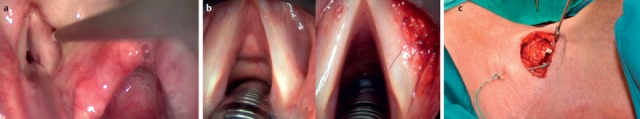
a – injection augmentation of the right vocal fold; b – implantation augmentation of the right vocal fold; c – left-sided thyroplasty type I with Friedrich prosthesis.

**Figure 29:**
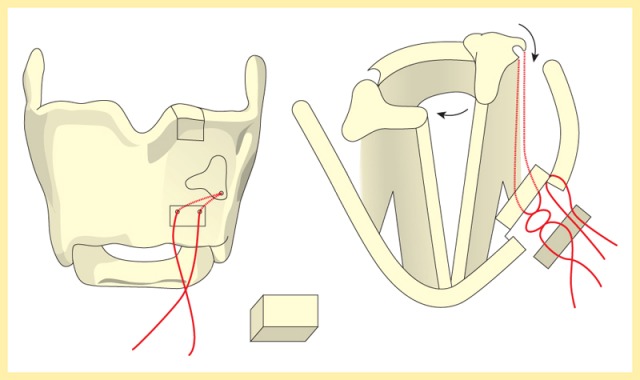
Thyroplasty type I with arytenoid adduction with cartilage

Insufficiencies of glottic closure at the membranous and/or cartilaginous part of the glottis caused by unilateral paresis and defect are permanent or transitory, partial or total [251]. They may lead to penetration and aspiration. To improve swallowing, different techniques are applied (modified according to Giraldez-Rodriguez [252], Figure 28 (Fig. 28), Figure 29 (Fig. 29)):

- Training of swallowing
- Injection and implantation augmentation
- Thyroplasty with/without arytenoid adduction
- Partial unilateral pharyngeal resection
- Myotomy of the cricopharyngeal muscle
- Tracheotomy

###### Injection and implantation augmentation

Injection and implantation augmentations can be performed transorally and transcervically. They are mainly applied in the treatment of low to moderate glottic insufficiencies (Figure 28 a, b (Fig. 28)). Those methods are still most frequently used in total as well as partial vocal fold paresis, but they are also useful for the treatment of vocal fold atrophies, e.g. in cases of presbylarynx, and of scarring of the vocal folds. More and more often, they successfully accompany interventions of the laryngeal skeleton. 

The aim is the medialization of the vocal folds by augmentation of the insufficient vocal fold, e.g. in paresis, defects, and scarring of the vocal folds. In this context, injection or implantation is performed laterally into the lateral part of the vocalis muscle or deeper between the thyroid cartilage and the vocalis muscle. Fat, fascia, cartilage, hyaluronic acid, hydroxyapatite, and silicone are normally used as material for augmentation [251]. 

###### Thyroplasty

Isshiki made medialization thyroplasty internationally known as type I thyroplasty [253]. It is predominantly applied in paresis and atrophies of the vocal folds, but also in dysphagia. The complex structure of the parts of the vocal folds relevant for the quality of the voice are not destroyed.

After previous creation of a defined thyroid cartilage window, a displacement of the lateral vocal fold in the direction of the midline is achieved by inserting and, if needed, fixing of autologous or allogeneic material. These materials are for example autologous cartilage (septum or thyroid cartilage), Gore-Tex, titanium clips, or silicone blocs [251] (Figure 28c (Fig. 28), Figure 29 (Fig. 29)). 

###### Arytenoid adduction

In 1978, Isshiki et al. were the first to describe arytenoid adduction as a surgical technique for correction of glottic insufficiency [253]. It is mostly combined with thyroplasty (Figure 29 (Fig. 29)). In the context of this intervention, first the posterior edge of the thyroid cartilage of the affected side is identified and, after creation of a cartilaginous window according to Maragos, the muscular process of the arytenoid cartilage is exposed [254]. A Vicryl thread (4×0) is then pulled through the muscular process and fixed ventrally at the inferior edge of the thyroplasty cartilage window. By rotating the arytenoid cartilage, improved glottic closure in the posterior part of the vocal folds is achieved, thus optimizing the voice and swallowing function.

##### 5.4.1.10 Myotomy of the upper esophageal sphincter

Myotomy is indicated when the upper esophageal sphincter does not or not sufficiently relax during swallowing [255], [256]. In cases of neurological diseases, the decision and the schedule should be made in agreement with an interdisciplinary team. Prior to the intervention, the function of the lower esophageal sphincter must be checked and documented. If chronic reflux problems are present, the indication for surgery must be critically discussed. If necessary, treatment of the lower esophageal sphincter must be integrated into the therapeutic concept. A test with botulinum toxins before intervention may be helpful. In some cases, botulinum toxins may even suffice as a one-off method. The location and extent of the myotomy depend on the location and extent of the muscular disorder identified during video-fluoroscopy in the lateral optical path.

Before the intervention, a Sengstaken-Blakemore tube is inserted into the upper esophagus and the hypopharynx. It is filled with 40–50 ml of water for female patients and with 50–60 ml of water for male patients. The incision is performed at the anterior edge of the sternocleidomastoid muscle and in a skin line at the level of the cricoid cartilage. The prevertebral space is reached between the neurovascular cord of the carotid artery, jugular vein and vagus nerve, and the hypopharynx. The extrinsic branch of the super laryngeal nerve must be preserved.

In order not to jeopardize the neural supply of the pharynx and esophagus, we prefer dorso-medial myotomy. The hypopharynx is turned to the surgeon with the inserted Sengstaken-Blakemore tube. Myotomy of the constrictor muscles then follows (Figure 26a, b (Fig. 26)).

Over the venous plexus and the mucosa under the muscles, the myotomy is extended to the upper esophagus and thyropharyngeal muscle. The extent of the myotomy is defined based on the radiological findings. Finest muscle fibers around the pharynx have to be transected in order to avoid failure. These fibers can be easily identified on the cuff of the Sengstaken-Blakemore tube. Small lesions of the mucosa are closed with atraumatic sutures. In such cases, a feeding tube is applied for several days. The possibility that the muscle stubs merge again is excluded by suturing them on the constrictor or longus colli muscle of the same side [245]. The disadvantage of lateral myotomies is that the neural supply of the constrictor muscle is partly destroyed on the side of the myotomy. 

###### Partial unilateral pharynx resection

In rare cases of vagus paralysis, the extension of the pharynx may be so excessive that a partial resection of the pharynx of the paralyzed side is indicated. The resection margins are marked with dye injections during video-endoscopic examination prior to the intervention.

The surgical procedure of partial resection of the pharynx corresponds to myotomy. The Sengstaken-Blakemore tube fills the paralyzed pharynx [245].

Muscular atrophy as a sequel of the paralysis may be the reason for an extremely thin pharyngeal wall. Together with the dye injections, the wall thickness determines the resection margins. If nasal aspiration is present, the paralyzed part of the nasopharynx must be closed. The suture of the pharyngeal defect is identical to the primary closure after unilateral resection of a pharyngeal tumor. Generally, these patients already have PEG. Two weeks after surgical treatment, swallowing training commences.

In those cases where surgery for diverticulum or partial unilateral pharynx resection has been performed, myotomy up to the esophagus should be performed at the same time.

##### 5.4.1.11 Elevation plasty of the interarytenoid region

**Figure 30:**
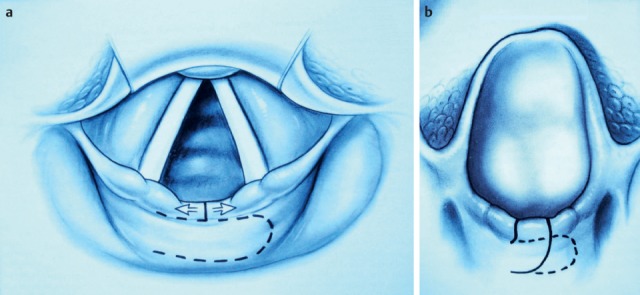
a – a low interarytenoid region may cause passage of saliva into the larynx, small transposition flaps are a therapeutic option; b – large transposition flap from the postcricoid region in order to establish the protection wall at the interarytenoid region.

In cases of slight interarytenoid aspiration where no or only slow progression of the disease is observed, an endoscopic, plastic-surgical intervention may reduce the existing problem. Saliva or liquids flow over the lowest point, i.e. the interarytenoid region, into the larynx. A postcricoid transposition flap that is also applied in small laryngeal gaps (grade 1) can lead to raising the interarytenoid region (Figure 30 (Fig. 30)).

Age-related swallowing disorders or incipient neurodegenerative diseases (Parkinson’s disease) more often reveal this type of dysphagia. Patients complain about a desire to clear their throats and coughing, both mainly when eating or drinking. In cases of sensitivity disorders, the complaints even occur at rest. The indication is made based on interdisciplinary cooperation, after functional endoscopy and video-fluoroscopy. Cricopharyngeal dysfunction or a tumor disease must be excluded.

After tracheostomy, the surgical intervention is performed. The distending laryngoscope is applied and the interarytenoid region is exposed. For treatment of aspiration, a mucosal flap of the postcricoid region is circumcised [34], [245] (Figure 30a (Fig. 30)). Only the mucosal incision is performed with the CO_2_ laser after identification of the arytenoid cartilage. The mucosa of the interarytenoid region is prepared to the side. The flap is freely prepared in a postcricoid direction. It is generously dimensioned as a transposition flap and turned into the prepared bed of the interarytenoid region, fixed with sutures, and sealed at the wound edges with tissue glue (Figure 30b (Fig. 30)).

##### 5.4.1.12 Transection of velar formations

**Figure 31:**
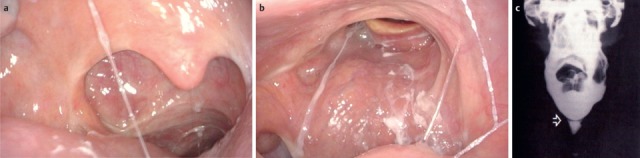
a – 87-year-old patient with significant oropharyngeal dysphagia after extended partial resection of the right oropharynx and right-sided neck dissection and radiochemotherapy (transoral endoscopy); b – very viscous saliva, scarred right-sided velum; the scars extend over the posterior oropharyngeal wall to the contralateral side, additionally there is a transportation disorder of the tongue (transnasal endoscopy); c – tissue formations (arrow) in the pharynx or esophagus cause globus sensation and/or dysphagia complaints (radiography, different patient).

Velar or tissue formations occur as a sequel of inflammations in the hypopharynx, upper esophageal entrance, and upper esophagus. These scarring changes can be transected with the laser and dissolved (Figure 31 (Fig. 31)).

#### 5.4.2 Special surgery for severe aspiration

Silent aspiration often occurs in combination with motor disorders and is characterized by reduced sensitivity. Like all motor dysfunctions, the extent of the disorders can range from very slight to severe forms of sensitivity loss. The progredient course of the disease should be included in the therapeutic concept. In order to gain time in such emergency cases and to solve the problem of silent aspiration that mostly arises in the context of severe neuromuscular or muscular disorders, the airways have to be protected against aspiration.

The following findings may be revealed during functional endoscopy:

- Overflow into the larynx without coughing
- Foamy saliva is diffusely spread in the oro- and hypopharynx; no inflammation
- Reduced bolus control
- Food residues in the pharynx
- Thickened viscous sticky saliva with simultaneously increased reflux

Anamnestic data provide important hints:

- TIA or stroke
- Tumors and/or sequelae of surgery in the posterior cranial fossa or the bulb area
- Neurodegenerative diseases
- Head injury
- Sequelae of surgery – scars, defects, etc.
- Late consequences of radiochemotherapy, etc.
- Fractures, strictures, and stenoses, etc.

The tracheostomy secures breathing; nutrition is secured via PEG. Silent aspiration must be stopped. Communication should be possible.

The following questions should be clarified:

- Is breathing regular?
- Is voice production possible?
- What about the muscular function of the oral cavity, the pharynx, larynx, and the trachea?
- Which innervation(s) are disturbed?
- What about the general condition of the patient?
- What is the prognosis?

Depending on the answers to these questions, the decision for surgical intervention is made on an interdisciplinary basis. Then, the head and neck surgeon defines the best treatment for the patient and tries to adapt it to the patient’s individual situation.

If the disease allows phonation on the glottic level, the corresponding techniques should be preferred [245]. If the structures relevant for articulation are damaged in such a way that the patient can no longer speak, surgical procedures in this regard are not indicated.

In certain cases, the reversibility of this surgery is an important factor. Based on results with reversible techniques, the former technique of total laryngectomy with surgical voice rehabilitation is indicated only in very rare cases.

##### 5.4.2.1 Laryngo-tracheal separation

Laryngo-tracheal separation is a surgical treatment of silent and/or severe aspiration without preservation of the individual's voice in order to achieve complete separation of the airway and the swallowing tract [257]. Ethically, it is a very difficult decision for every surgeon because normal laryngeal or tracheal tissue is surgically altered and the procedure has a high complication rate of 58% and an inefficiency of 27% [258]. Correspondingly, Hara et al. [259] reported on successful laryngo-tracheal separation in two thirds of their neurologically impaired pediatric patients. Laryngo-tracheal separation is a reversible procedure.

##### 5.4.2.2 Supraglottic closure of the larynx

Supraglottic separation can be performed by means of transoral endoscopy. The epiglottis is mobilized ventrally and laterally and the cartilage is incised and reduced for reduction of the tension. Afterwards, partial de-epithelization of the aryepiglottic folds is performed. The epiglottic flap is turned in a dorsal direction and sutured over the laryngeal entrance with the aryepiglottic folds. After closure, phonation is no longer possible. However, the procedure is reversible.

##### 5.4.2.3 Plastic closure of the airway between larynx and trachea with preservation of the individual's voice and of the continuity of the membranous wall of the trachea

The aim of reversible tracheal closure by means of laryngo-hyoidopexy (LHP), suspension to the chin and subsequent restitution of the individual's voice, is that the patient is able to speak after the intervention with his own voice without aspiration [256]. LHP with suspension is necessary to control the quantity of aspiration below the vocal folds and above the closure, as otherwise the quality of the voice may be impaired.

After transection of the thyroid isthmus, the trachea is exposed via a median incision from the thyroid cartilage into the jugular fossa. The anterior tracheal wall is split at the level of the 1st to fourth tracheal ring in the median line or opened in the form of a swing door. A small tube is inserted at the level of the fourth tracheal ring for ventilation.

**Figure 32:**
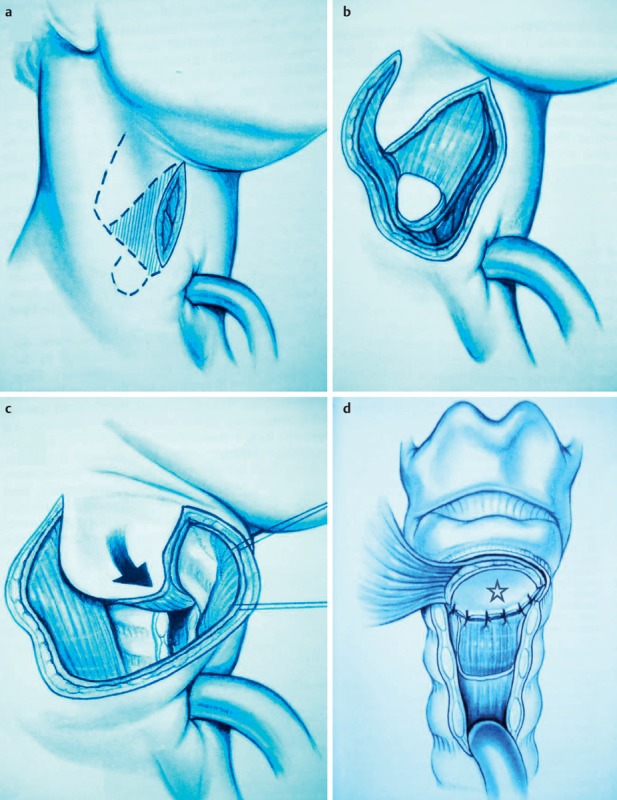
a – incision for the skin-platysma-fascia graft, hatched areas = parts of the flap are de-epithelized in order to place them under the skin, via the paramedian incision, the trachea and cricoid cartilage are exposed; b – the graft is circumcised, the platysma with its fascia on the inferior surface is prepared from the cervical muscles; c – after suturing the cranially pedicled mucosal flap from the posterior tracheal wall as covering on the side of the trachea, the graft is also used for defect covering of the donor site of the mucosal flap; d – donor site of the cranially pedicled mucosal flap of the posterior tracheal wall (star) is visible, partial resection of a part of the 1st and/or 2nd tracheal ring in order to avoid pressure on the pedicle.

A cranially pedicled mucosal flap of the membranous wall is circumcised, shifted upward, and sutured in such a way that it closes the trachea at the level of the first or second tracheal ring with the mucosa without tension. Caudal covering of the closure is performed with a island skin-platysma-fascia-flap, fascia graft (Figure 32 (Fig. 32)).

After planning of the flap, the hatched area of the pedicle is de-epithelized so that it may heal in under the skin. The pedicle should be sufficiently large in order to secure good supply to the flap. The graft is dimensioned so that it covers the inferior surface of the cranially pedicled mucosal flap and the donor defect at the posterior tracheal wall without tension. Both flaps are fixed together with fibrin glue. In order to avoid pressure on the pedicle, the anterior part of the second tracheal ring is resected. A slight tamponade stabilizes the position of the graft. It is recommended that a bigger tracheostoma be created and the skin be sutured with the skin-platysma fascia graft with slight tension at the superior circumference of the tracheostoma. The same applies for the suture of the other parts of the tracheostoma between skin and tracheal mucosa. Generally, the insertion of a cannula after extubation is not necessary.

**Figure 33:**
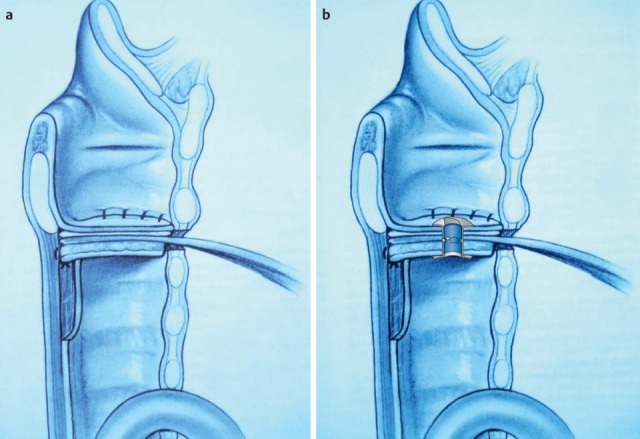
a – the donor site of the cranially pedicled mucosal flap is covered by the skin-platysma-fascia graft; b – two months after healing, a shunt valve is inserted into the tracheal closure after puncture; speaking is possible with the patient's own, individual voice.

Two months after intervention, secondary restauration of the voice is performed by means of a puncture through the tracheostoma closure with insertion of a shunt valve (voice prosthesis) (Figure 33 (Fig. 33)).

Speech therapy may start a week after insertion of the shunt valve.

Three patients have already been treated successfully in this way. One of these patients suffered from a slowly growing malignant tumor. The patients speak/spoke with their own voices. The maximal follow-up time after this technique amounts to 22 years. The female patient went blind in 1991 because of hemangio-neuroblastoma and, after tumor-surgery, lost the function of the brain nerves IX, X, XI, XII. The consequence was, among other things, a myoclonus of the velum, pharynx, and larynx as well as complete sensitivity loss of pharynx, larynx, and trachea. Meanwhile, three other hemangio-neuroblastomas that developed in the area of the medulla oblongata have been removed surgically. As sequelae, further sensitivity losses occurred in the area of the hands and neck so that, today, the patient has to close the tracheostoma with the back of her hand. She can no longer find the tracheostoma with her finger. Despite this problem, she graduated from university and has been married for several years.

Like the other two patients as well, during the already-mentioned long follow-up time, the only complication were granulations at the shunt. The shunt-valve has to be exchanged about every 7–10 months after this technique.

Other techniques have been described that help to avoid the ultima ratio of laryngectomy and that allow phonation via a voice prosthesis in the pharynx [260].

##### 5.4.2.4 Laryngectomy

Dysphagia and laryngectomy may interrelate in different ways. Laryngo-tracheal separation or laryngectomy are applied therapeutically for irreversible, neurological, or oncological diseases with severe aspiration. Laryngectomy is the most radical method of preventing aspiration. Laryngectomy with partial resection of the hypopharynx leads to swallowing disorders (Figure 11 (Fig. 11)).

Severe dysphagia is often the result of therapy of advanced laryngeal and hypopharyngeal carcinomas. In this context, patients have the poorest result in their swallowing function after radiochemotherapy and/or laryngectomy [261]. Stenosis in the neopharynx can be prevented by primary application of a radialis-forearm flap. Bougienage of the stenosis may improve the patient’s situation. Sweeny et al. [262] evaluated stenoses of the neopharynx or the pharyngo-esophageal transition after laryngectomy. Stenoses were found to occur in 19% of laryngectomized patients. 82% of these stenoses became apparent within the first postoperative year. There was no difference between primary and salvage laryngectomy. Patients who underwent bougienage or dilatation only once had a better prognosis regarding dysphagia than laryngectomized patients after several dilatation procedures.

#### 5.4.3 Surgical therapy of swallowing disorders after radiochemotherapy

By means of modified fluoroscopic barium swallowing examination and endoscopy in patients with head and neck carcinomas after primary radiochemotherapy or surgical therapy combined with radiochemotherapy, in 11% of the cases Nguyen [39] could demonstrate stenoses in the pharynx or esophagus that led to persisting dysphagia after therapy. These stenoses were treated with dilatation. In 50% of the patients, dilatation performed once was sufficient for dysphagia treatment. In 21%, dilatation was needed at least 4 times.

Hutcheson et al. [263] analyzed 23 recurrence-free patients with laryngo-pharyngeal dysfunction who had to be laryngectomized because of tumor therapy (Table 24 (Tab. 24)). These accounted for 6% of all total laryngectomies in an evaluation interval of 6 years. All patients with laryngo-pharyngeal dysfunction had to undergo primary radiotherapy or radiochemotherapy because of a carcinoma of the head and neck.

#### 5.4.4 Tracheotomy

Numerous patients in intensive care units require an access to the deep airways in order to secure breathing and breathing control. Indications are prolonged intubation, support of breathing, pulmonary management of secretion, obstruction of the upper airways with stridor, dyspnea, intercostal retractions, OSAS, bilateral paresis of the vocal folds, missing possibilities of intubation, extensive interventions of the head and neck region, trauma management, and protection of the airways in neurological diseases [264]. As a short-term measure, the airway is closed by a blocked tube in sedation.

An early tracheostomy, about 72 hours after intubation, is not associated with disadvantages [265]. Generally it is indicated when ventilation will probably be needed for more than 12 days [266].

Dysphagia with aspiration may be a reason for creating a tracheostoma in non-intubated patients. On the other hand, tracheostomy may lead to dysphagia as a complication. Generally, it is performed either as percutaneous dilatation or surgical tracheostomy [267], while in intensive care units, dilatation tracheotomy is most frequently applied and is equally safe [268], [269]. According to Bause [270], contraindications against puncture tracheotomy are an acute respiratory insufficiency (emergency situation), non-correctable combined coagulation disorders, and high-grade circulatory instability.

Indications for conventional tracheotomy are:

- Patients below the age of 18
- Impossibility of tracheal puncture
- Instable fracture of the cervical spine
- Tracheal tumors
- All supratracheal airway obstacles
- Fresh tracheal suture
- Necessary size of the cannula of >10 mm (inner diameter)
- Necessity of side separate ventilation
- Fresh bronchial suture
- Pre-existing tracheomalacia
- Definitive tracheostoma
- Mobile, non-ventilated patients

For open surgical tracheostomy, transsection or displacement of the thyroid isthmus in a caudal direction and incision between the second and third tracheal ring are recommended [267], [271]. A Björk flap is not necessary. In cases of expected long-term tracheostomies, a careful skin-mucosa suture is desirable for a stable tracheostoma in order to prevent granulations and stenoses and to secure ventilation.

Surgical procedure: A skin incision measuring about 4 cm is placed. The linea alba is presented; the infrahyoid muscles and the thyroid isthmus are displaced in a lateral direction or transsected. The trachea is most frequently opened between the second and third tracheal ring. The anterior part of the second tracheal ring is subcutaneously sutured with the superior edge of the skin incision and the third one is subcutaneously sutured with the inferior edge of the skin incision. Intensive and long-term ventilated patients may receive a permanent stoma in the same way in cases of a lower larynx between the first and second tracheal rings. Otherwise the tracheostoma widens because of a pressure-related upward shift of the cannula. This makes it difficult to seal the blocking cuff of the cannula in ventilated patients. The last-mentioned technique has the following advantages in relation to swallowing disorders: safe opening by adaptation of the stoma edges at the skin incision and smaller tracheostoma. A variation of the tracheal opening is the vertical incision over several tracheal rings [264]. This procedure is recommended only in emergency cases because of the better overview it affords.

Changing the cannula is possible from the second postoperative day onwards. A speech valve positioned on the tracheal cannula promotes speech and swallowing therapy [272].

A blocked tracheal cannula reduces the main risk of dysphagia, i.e. aspiration. However, it cannot prevent saliva from flowing into the trachea. A transnasal feeding tube increases saliva production. Since the regular saliva production of about 0.6–1.5 l per day is too much for the airway – even if only part of it is aspirated – the patient must be carefully monitored. In cases of aspiration, the saliva accumulates above the cuff and can be suctioned via the subglottic device of the blocked tracheal cannula. Professional care of tracheostoma patients is necessary in order to prevent inflammation of the tracheal wounds which might be caused by saliva and colonization of germs.

#### 5.4.5 Percutaneous endoscopic gastrostomy (PEG)

PEG is an established endoscopic surgical procedure for patients suffering from dysphagia who are not able, for a determined time or even permanently, to care for the necessary daily intake of liquid or calories. In this context, nutrition via PED tube secures the energy supply. Indications for PEG are obstructive or neurogenic dysphagia with reversible and irreversible swallowing disorders as well as aspiration [273]. Depending on their abilities, patients may still pursue oral food intake. Currently, the method of pulling a thread according to the Seldinger technique and direct puncture are established procedures, whereby the method of pulling through a thread is considered as a safe and simple method that is most frequently applied. In prospective clinical studies, it was possible to confirm that nutrition via PED is well accepted and tolerated. Mays et al. [274] developed predictors for the application of PEG tubes in order to secure enteral nutrition in the context of therapies. Influencing factors are preoperative irradiation, supraglottic partial resection of the larynx, tracheostomy, clinical status of the cervical lymph nodes (N0 vs. N2, N1 vs. N2), preoperative weight loss, dysphagia, type of reconstruction, and the tumor stage. Mays prefers the early application of PEG so that complications in the context of postoperative wound healing can be avoided in high-risk patients, and the prognosis and quality of life can be improved.

## Notes

### Competing interests

The authors declare that they have no competing interests.

## Figures and Tables

**Table 1:**
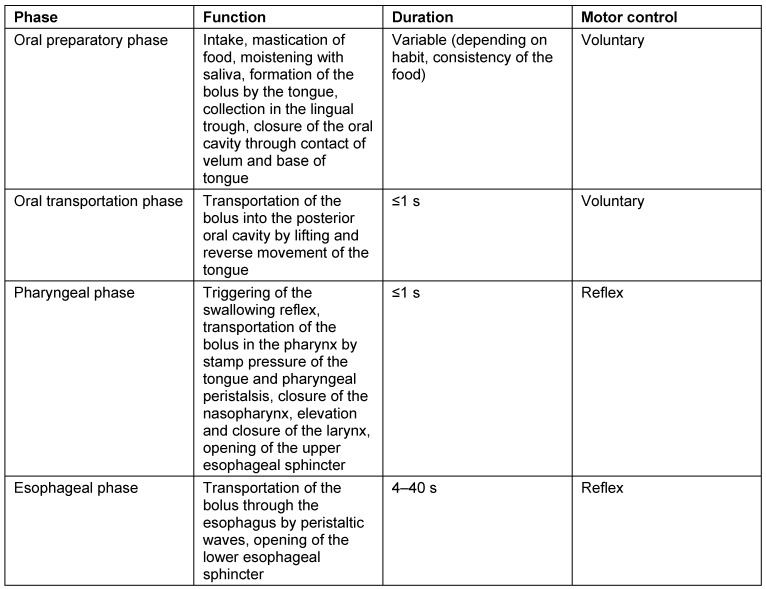
Phases of swallowing (according to [37])

**Table 2:**
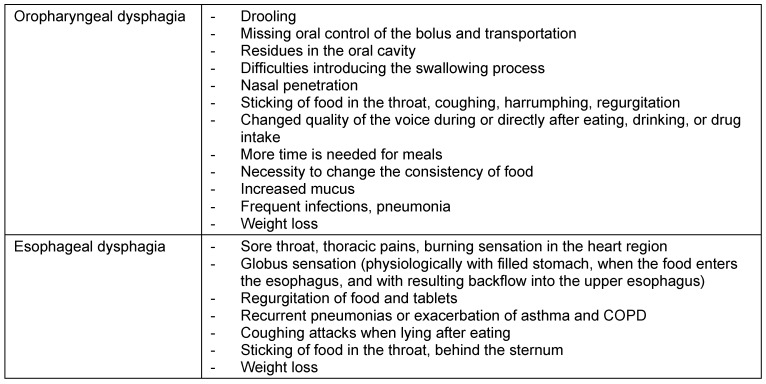
Symptoms of oropharyngeal and esophageal dysphagia (modified according to [36])

**Table 3:**
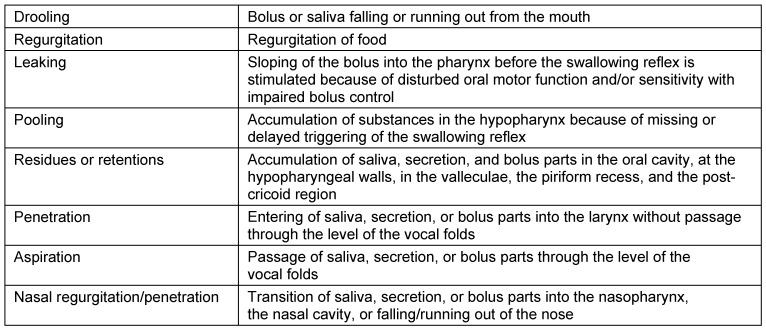
Overview of the nomenclature regarding symptoms of swallowing disorders

**Table 4:**
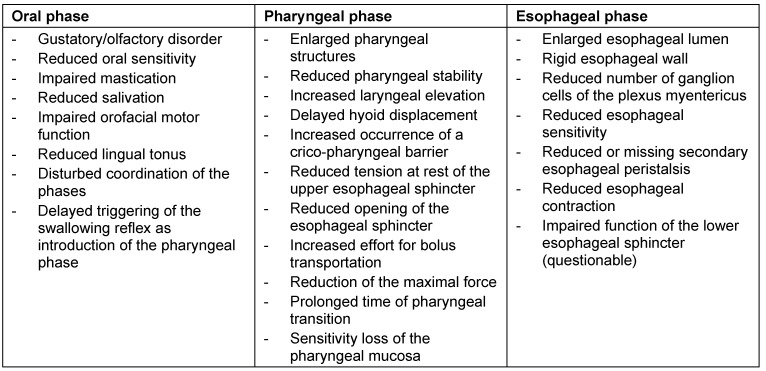
Age-related changes in the single swallowing phases leading to presbydysphagia (modified according to [17])

**Table 5:**
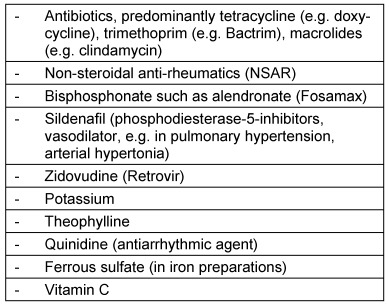
Drugs possibly inducing OMIEI (modified according to [1, 275])

**Table 6:**
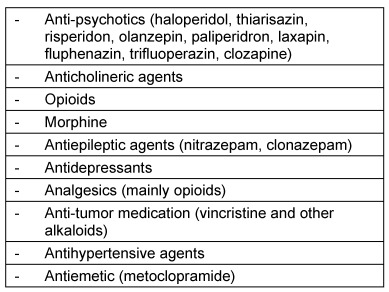
Centrally effective drugs possibly inducing or increasing dysphagia (according to [1, 36])

**Table 7:**
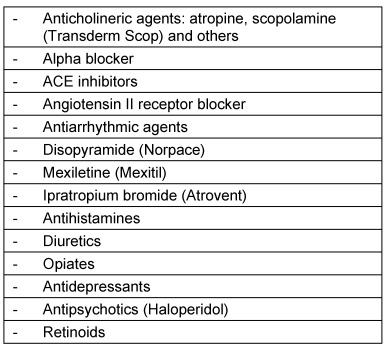
Drugs possibly inducing xerostomia (modified according to [36, 275])

**Table 8:**
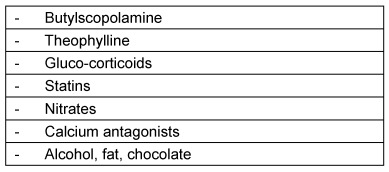
Drugs and substances possibly inducing motility disorder of the esophagus or tonus reduction of the lower esophageal sphincter (myopathy with dysphagia) (modified according to [275])

**Table 9:**
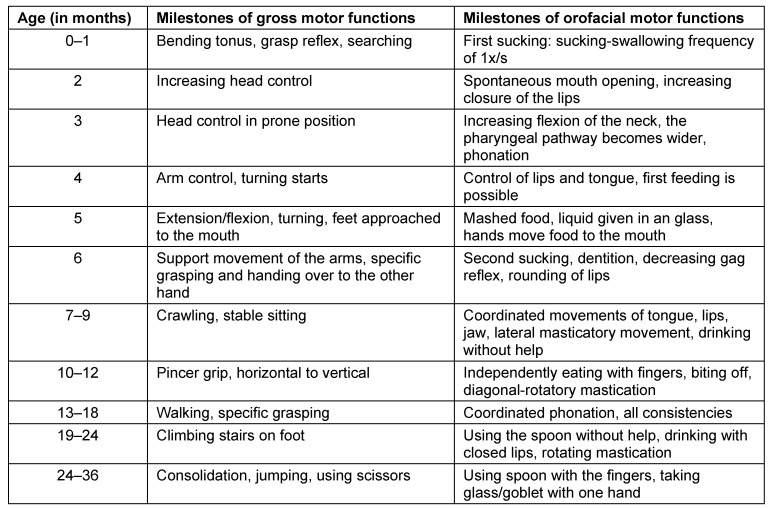
Synopsis of gross motor and orofacial motor development

**Table 10:**
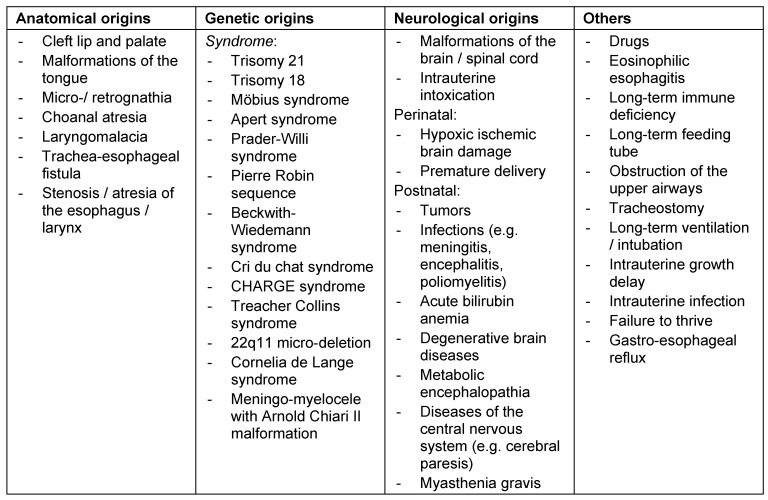
Different origins of pediatric swallowing disorders (modified according to Kühn [80] and Biber [276])

**Table 11:**
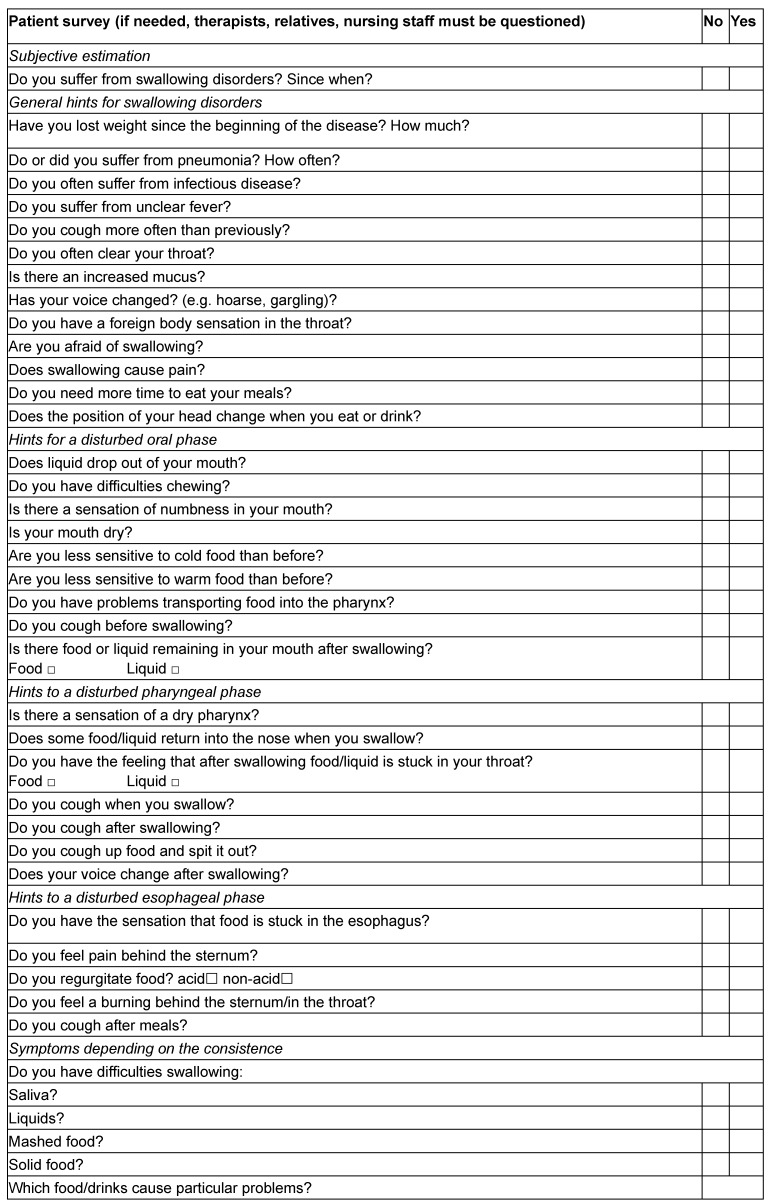
Questionnaire for anamnesis of swallowing disorders

**Table 12:**
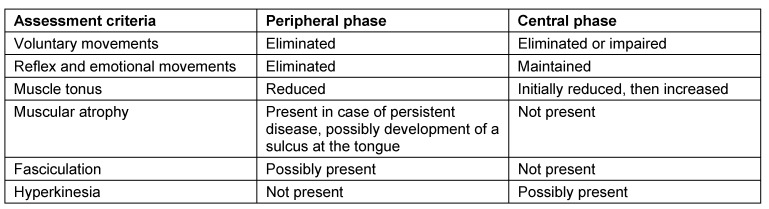
Differentiation of peripheral and central mobility disorders of the swallowing/speech organs

**Table 13:**
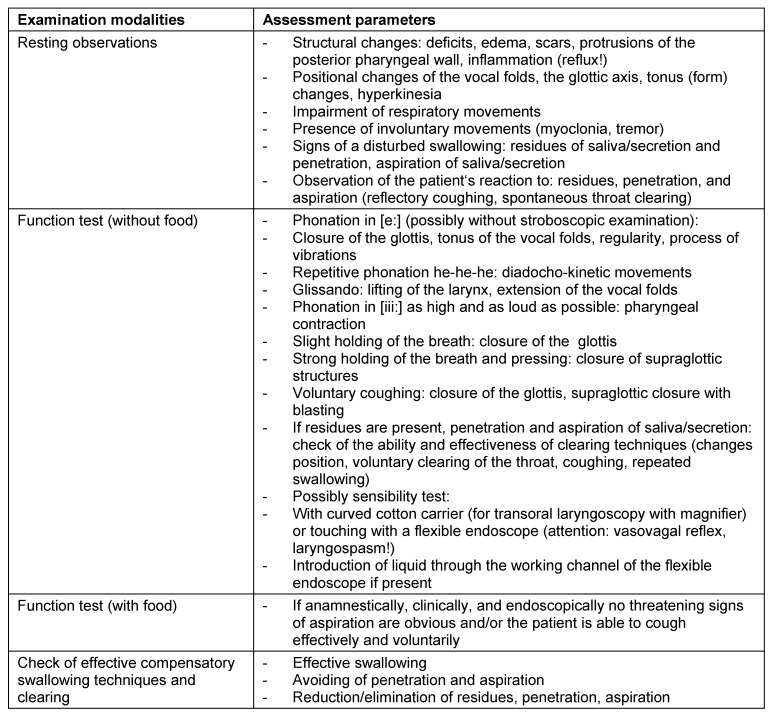
Assessment parameters of pharyngo-laryngoscopic examination in dysphagia

**Table 14:**
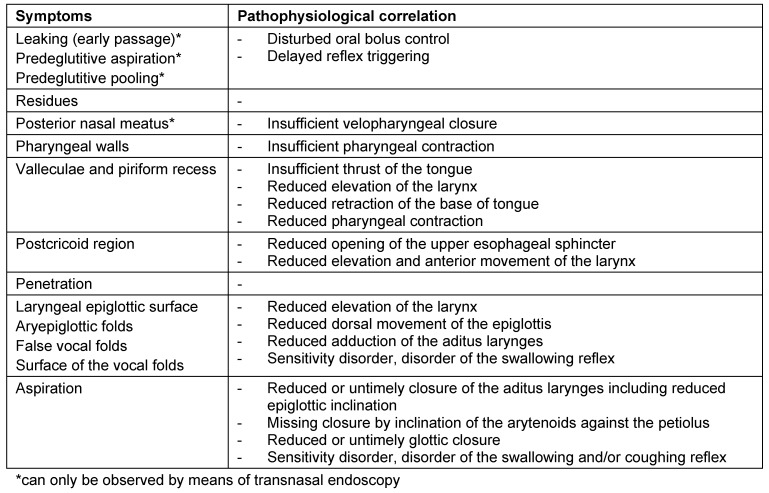
Observable symptoms through pharyngo-laryngoscopic examination and their pathophysiological correlation in swallowing disorders [37]

**Table 15:**
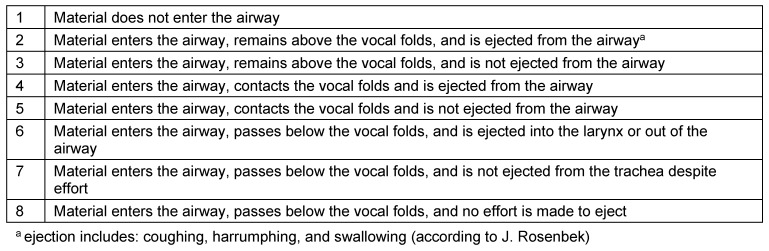
Penetration-aspiration scale according to Rosenbek [211]

**Table 16:**
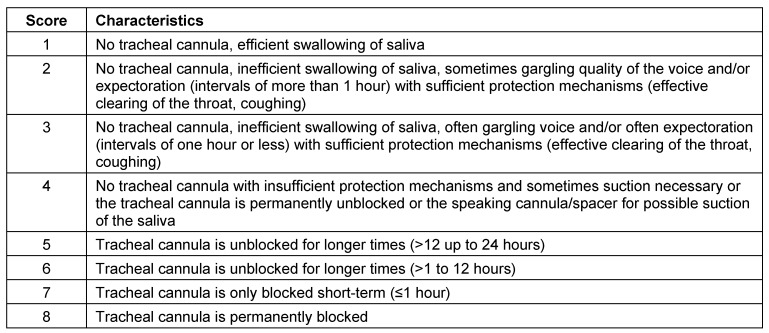
Bogenhausener Dysphagia Score, BODS-1 (swallowing of saliva)

**Table 17:**
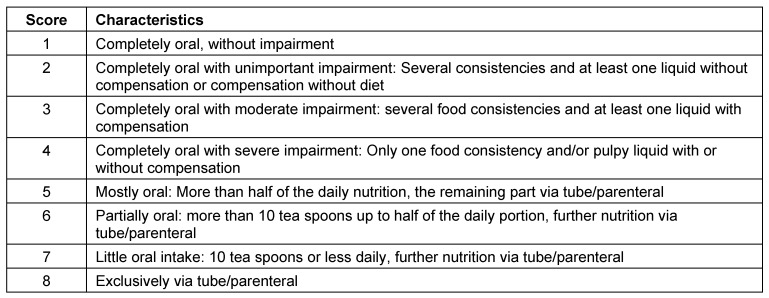
Bogenhausener Dysphagia Score, BODS-2 (food intake)

**Table 18:**
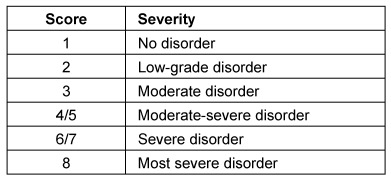
Single evaluation of the severity of the disorder according to BODS-1 (swallowing of saliva) or BODS-2 (food intake)

**Table 19:**
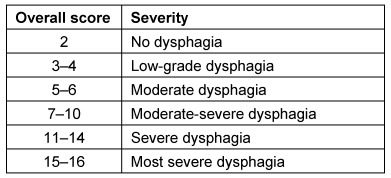
Overall evaluation of the severity of dysphagia according to BODS-1 (swallowing of saliva) and BODS-2 (food intake)

**Table 20:**
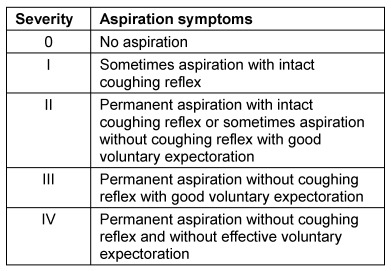
Classification of severity of aspiration according to laryngoscopic findings

**Table 21:**
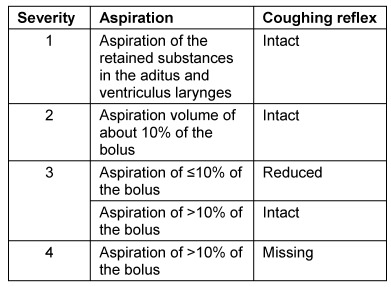
Severity of aspiration according to radiological findings

**Table 22:**
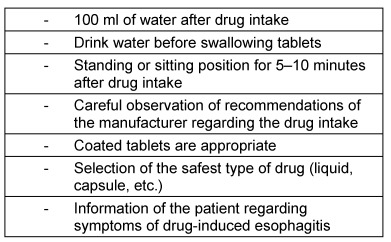
Recommendations for the prevention of drug-induced esophagitis

**Table 23:**
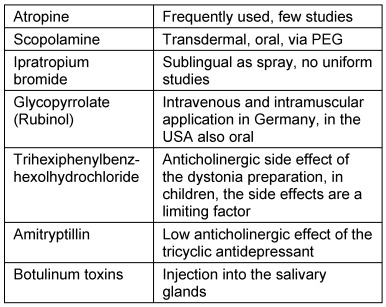
Drug therapy for hypersalivation

**Table 24:**
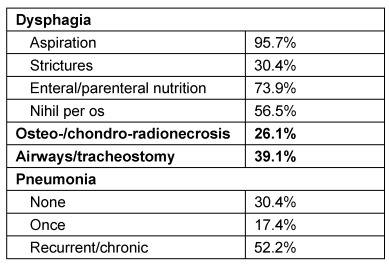
Indications for total laryngectomy in patients with laryngo-pharyngeal dysfunction according to Hutcheson [263] – complications and functional disorders
